# Molecular cloning of novel transcripts of human kallikrein-related peptidases 5, 6, 7, 8 and 9 (*KLK5* – *KLK9*), using Next-generation sequencing

**DOI:** 10.1038/s41598-017-16269-6

**Published:** 2017-12-11

**Authors:** Panagiotis G. Adamopoulos, Christos K. Kontos, Andreas Scorilas

**Affiliations:** Department of Biochemistry and Molecular Biology, National and Kapodistrian University of Athens, Athens, 15701 Greece

## Abstract

Alternative splicing of cancer-related genes is a common cellular mechanism accounting for cancer cell transcriptome complexity and affecting cell cycle control, proliferation, apoptosis, angiogenesis, invasion, and metastasis. In this study, we describe the discovery and molecular cloning of thirty novel transcripts of the human *KLK5*, *KLK6*, *KLK7*, *KLK8* and *KLK9* genes, using 3′ rapid amplification of cDNA ends (3′ RACE) and NGS technology, as well as their expression analysis in many established cell lines, originating from several distinct cancerous and normal tissues. Extensive bioinformatic analysis revealed novel splice variants of these five members of the *KLK* family, comprising entirely new exons, previously unknown boundaries of the already annotated exons (extensions and truncations) as well as alternative splicing events between these exons. Nested RT-PCR in a panel of human cell lines originating from seventeen cancerous and two normal tissues with the use of variant-specific pairs of primers was carried out for expression analysis of these novel splice variants, and Sanger sequencing of the respective amplicons confirmed our NGS results. Given that some splice variants of *KLK* family members possess clinical value, novel alternatively spliced transcripts appear as new candidate biomarkers for diagnostic and/or prognostic purposes and as targets for therapeutic strategies.

## Introduction

Cancer has become one of the major causes of death worldwide. Despite the fact that research has offered considerable advances in our understanding of carcinogenesis, tumor invasion, and metastasis, cancer still remains the most serious public health issue. Over the last decades, several genes have been considered as carcinogenesis mediators, thus being under the spotlight in a plethora of clinical and therapeutic studies. For instance, oncogenes and tumor suppressor genes have been found to be primarily involved in carcinogenesis, due to their ability to promote or suppress the formation of tumors, respectively^1,2^. In addition, deregulation of apoptosis-regulating genes, such as members of the *BCL2* gene family^3^, can cause defects in the cell cycle mechanism leading to increased cell proliferation and carcinogenesis, while genes encoding for matrix-degrading proteases, adhesion proteins, and motility factors can also play a significant role in tumor cell invasion and metastasis^4^.

The continuous evolution of human genome research techniques has offered new insights regarding the mechanisms and potential treatment of several malignancies. One of the most recent, powerful, and promising tools for genome analysis that will undoubtedly accelerate our understanding of many fundamental biological processes is Next-Generation Sequencing (NGS). NGS has the potential to reveal a variety of disease-related genetic alterations that previous techniques could not detect. The impact of NGS on the analysis of the human cancer cell genome is enormous. High-throughput sequencing analyses of cancer cell genomes have already revealed somatic mutations in tumors that account for constitutive activation of signaling pathways, usually mediated by activated growth factor receptors. For instance, 40% of melanoma cases are characterized by activating mutations affecting the structure of the BRAF protein, resulting in constitutive signaling through the MAPK pathway^5^.

High-throughput RNA sequencing (RNA-seq), one of the newest applications of NGS, enables the determination, characterization, and quantification of the expressed RNA molecules in the sample(s) of interest, and can hence lead to important biological discoveries regarding the human transcriptome^6,7^. Despite the need for bioinformatic tools for NGS data analysis, RNA-seq possesses several major advantages over classical gene expression analysis, including its high sensitivity and accuracy, broad dynamic range, nucleotide-level resolution, and – most importantly – its ability to analyze pre-mRNA alternative splicing and to detect novel mRNA transcripts. NGS along with molecular cloning techniques such as rapid amplification of cDNA ends (RACE) can provide useful information about alternative transcription start sites and polyadenylation sites that are usually exploited and which increase transcriptome complexity^8–10^. In fact, many human genes use alternative first exons, each one being preceded by its own promoter. Alternative promoters can direct tissue-specific gene expression and influence alternative splicing at the genomic level^11,12^. It is estimated that 58% of the transcribed mammalian genes have multiple promoters and generate alternative transcripts differing in their 5′ untranslated region (5′ UTR)^13^. Furthermore, about half of human genes use alternative cleavage and polyadenylation to generate mRNA transcripts that differ in the length of their 3′ untranslated region (3′ UTR) while producing the same protein. 3′ UTRs can regulate gene expression, translation efficiency, protein localization, as well as stability of the respective transcripts^14,15^.

The ability of NGS to uncover novel alternative splicing events is very important, as specific alternative splice variants are associated with particular diseases. For instance, alternative splicing of the vascular endothelial growth factor A (*VEGFA*) gene leads to the production of multiple protein isoforms, which display either pro- or anti-angiogenic activities. Under normal cellular conditions, the expression levels of these isoforms are correctly and tightly regulated. However, in cancer cells the expression of these isoforms is highly deregulated, suggesting that cancer cells can manipulate regulatory mechanisms to express particular isoforms that can functionally promote cancer cell growth^16^. Sequencing analyses have revealed that 92–94% of human genes can undergo alternative splicing, therefore producing multiple transcripts^17^, with an average of six transcript variants per gene. Alternative splicing is highly regulated according to cell type, developmental stage, and disease conditions^18–20^. However, several reports have demonstrated the expression of aberrant and abnormal splice variants in cancer cells or tissues. This phenomenon is attributed to the great instability of the human genome in cancer cells, leading to frequent sequence substitutions as well as aberrant alternative splicing events, which produce erroneous and dysfunctional proteins^21^. In addition, it should be mentioned that almost 50% of human genetic diseases are related to mutations in splice sites and regulatory elements, such as enhancers and silencers, resulting in alternative exon constitution^22–24^. For this reason, alternative transcripts produced by aberrant splicing events may represent promising cancer biomarkers.

**Figure 1:**
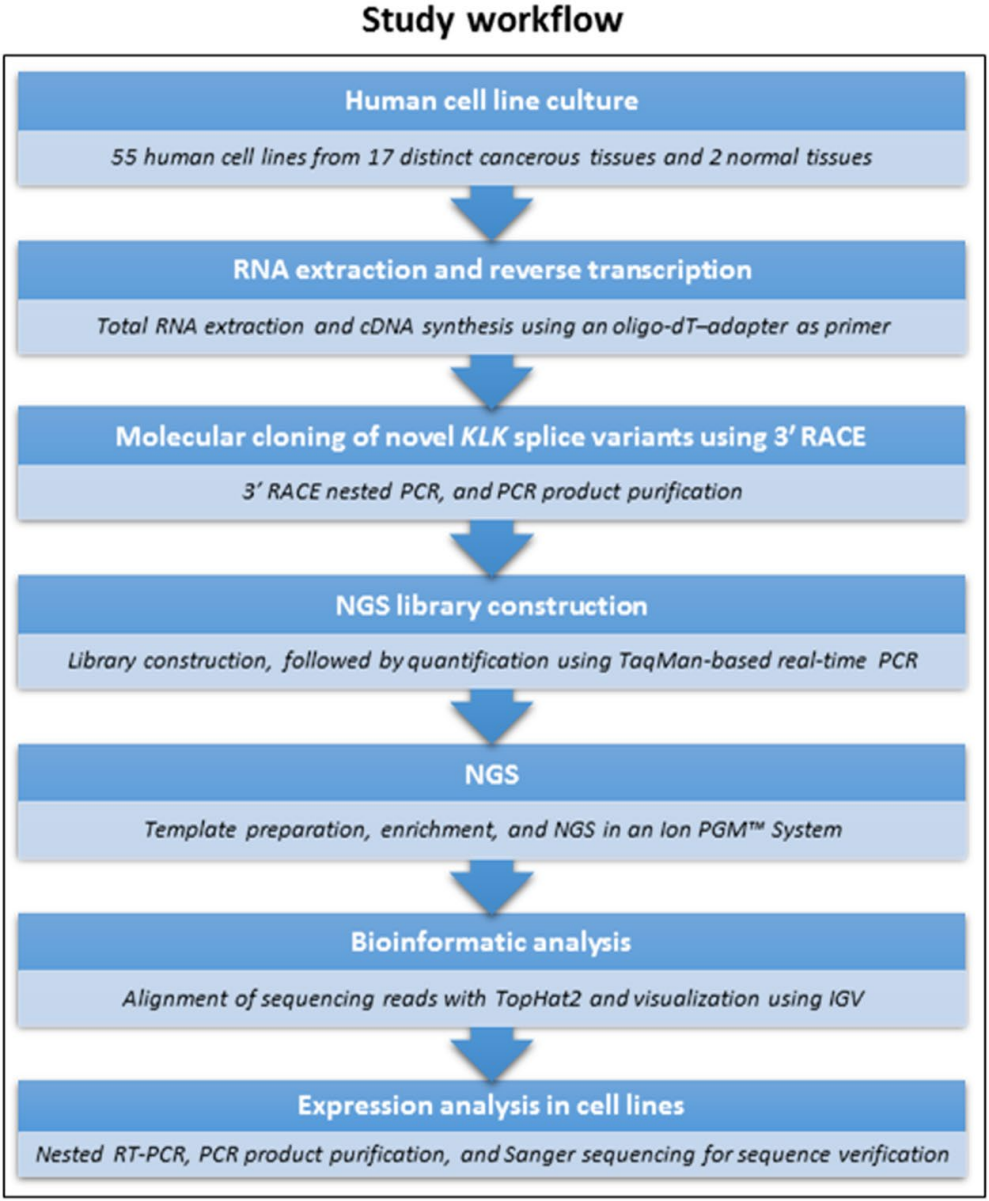
Flow chart presenting step-by-step the experimental workflow that was followed in the current study.

The gene family of tissue kallikrein and kallikrein-related peptidases (*KLK*s), consisting of 15 members (*KLK1* – *KLK15*), encompasses the largest contiguous cluster of serine proteases in humans^25^. The *KLK* genes share many common features and exhibit high similarity as they consist of five coding exons of similar size, with conserved intron phases^26,27^. Alternative splicing events that generate multiple *KLK* transcripts include exon skipping, mutually exclusive exons (special case of exon skipping), alternative 5′ and 3′ splice site usage, intron retention, exon extensions or truncations, and use of alternative polyadenylation sites. In this study, we describe the discovery and molecular cloning of novel *KLK* transcripts with the use of 3′ RACE nested PCR and NGS technology, and their expression analysis in a series of established cell lines, originating from several cancerous and/or normal human tissues (Fig. 1).

## Results

### Bioinformatic analysis of the NGS data uncovers new alternative splicing events in the *KLK5* – *KLK9* genes, following 3′ RACE nested PCR

**Figure 2:**
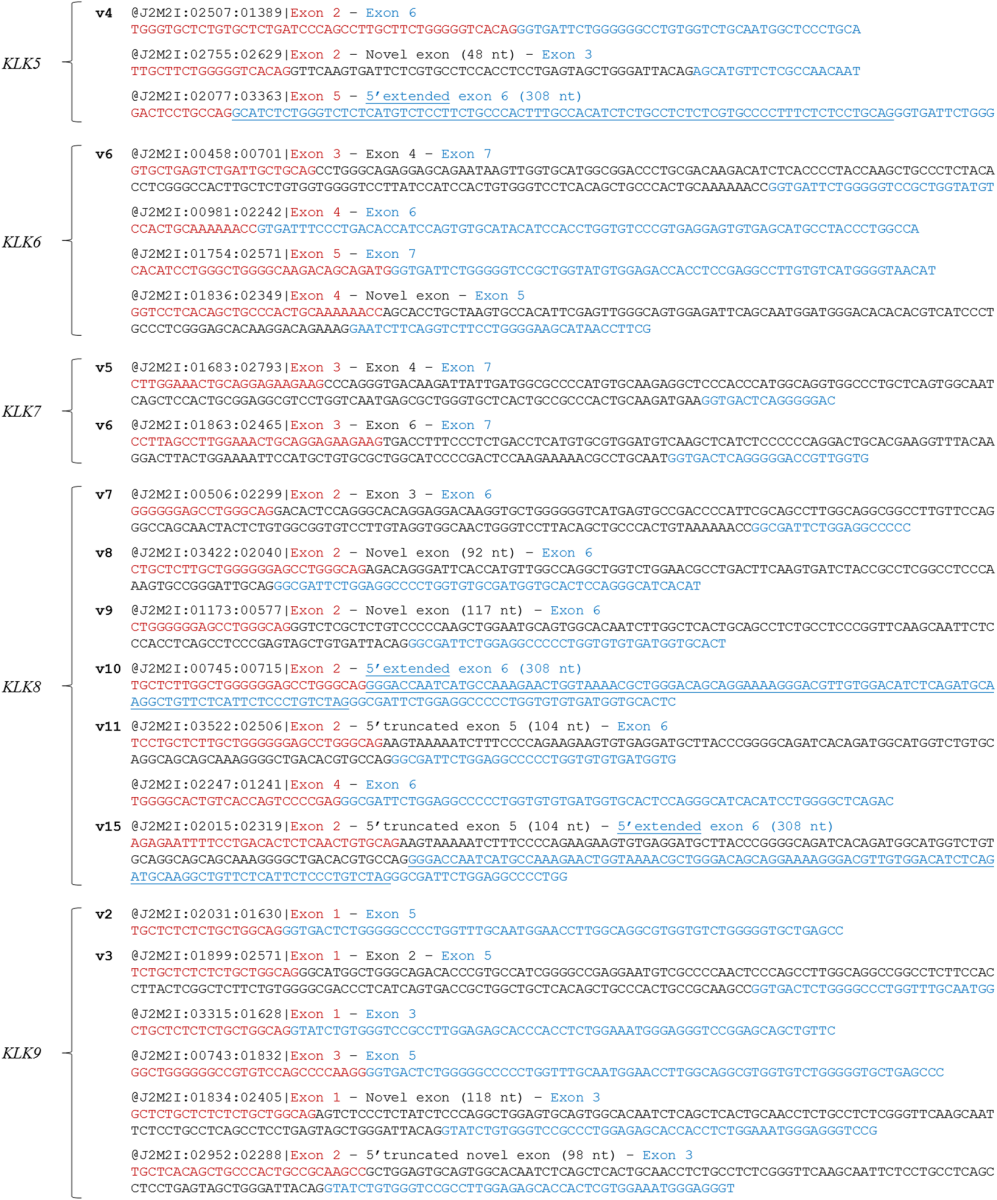
Representative sequencing reads for all novel alternative splicing events that were identified for *KLK5* – *KLK9*. The nucleotides of each exon are shown in different color (red, black, or blue) and exon extensions are underlined.

After having performed NGS on the Ion PGM™ System, a FASTQ file containing the sequencing reads derived from 3′ RACE nested PCR in a pool of cDNAs from 55 human cell lines was obtained. Then, the FASTQ file was uploaded to the open source, web-based GALAXY platform, where the TopHat2 algorithm was used for the alignment of sequencing reads against the human reference genome (GRCh38), as described in Methods. NGS provided about 4.5 million sequencing reads, 88% of which were successfully aligned to GRCh38. Alignment quality including mean coverage depth is shown in Supplementary Fig. 1. Bioinformatic analysis and visualization using the Integrative Genomics Viewer (IGV) uncovered a large number of novel splice junctions between annotated exons of *KLK5* – *KLK9* genes, new exon boundaries (extended or truncated), as well as several novel exons of these five genes (see Supplementary Fig. 2). Representative sequencing reads highlighting these new alternative splicing events are shown in Fig. 2.

### Novel *KLK5* transcripts

**Figure 3:**
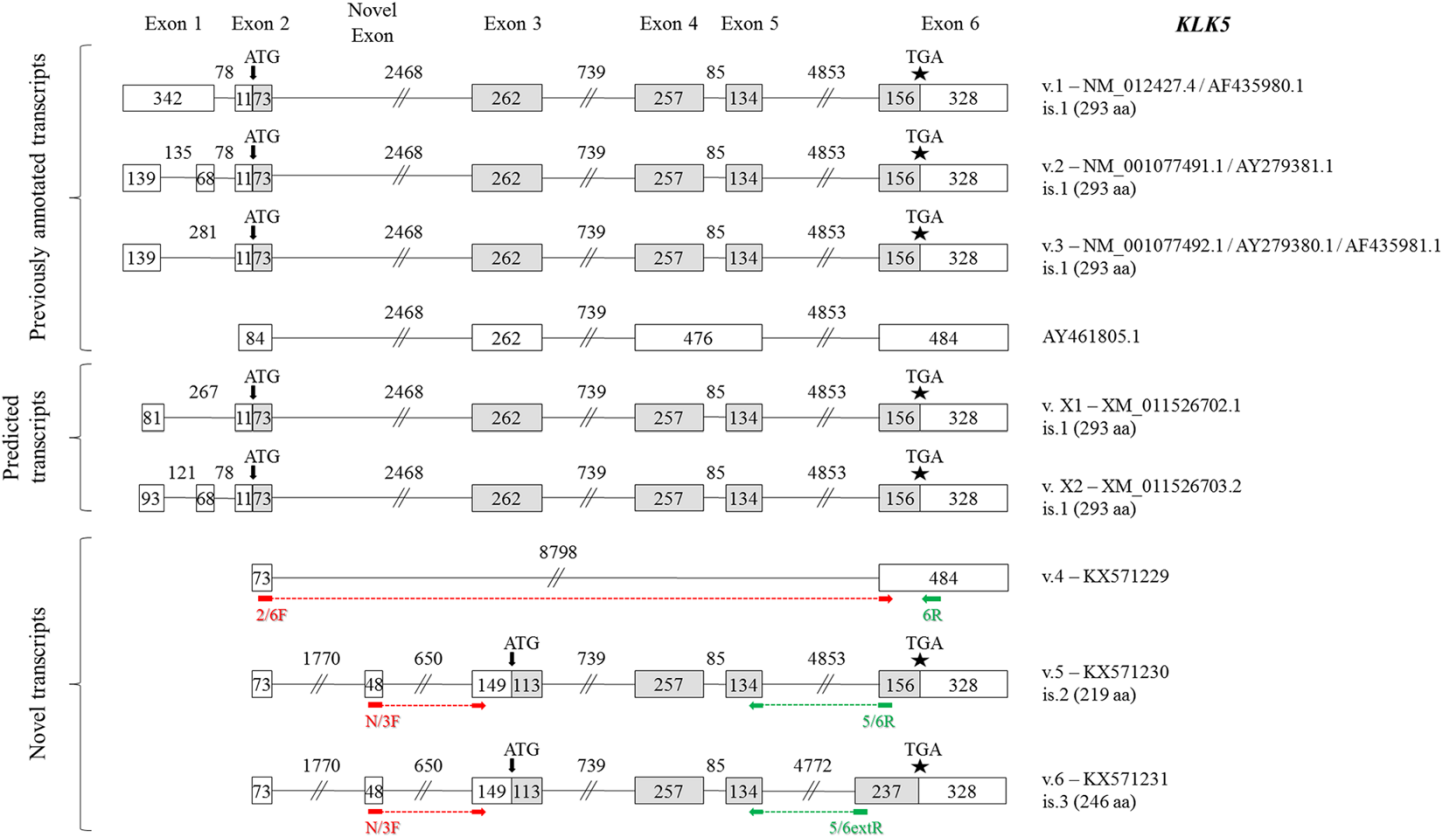
Detailed structure of the *KLK5* splice variants and their respective protein isoforms. Exons are depicted as boxes and introns as lines; gray and white boxes represent coding and non-coding exons, respectively. Numbers inside boxes and above lines indicate the length of each exon or intron in nucleotides. Arrows (↓) show the position of the ATG codon, asterisks (*) indicate the position of the stop codon, and question marks (?) represent an undetermined 5′ UTR. For each new transcript (*KLK5* v.4 – v.6), the splice variant number, the GenBank^®^ accession number (KX571229 – KX571231, respectively) and the protein isoform number (only for splice variants that are predicted to be coding transcripts) are shown next to each transcript. The target regions of the forward (red color) and reverse (green color) variant-specific primers used for expression analysis in nested RT-PCR are also demonstrated.

Three experimentally validated transcripts of the human *KLK5* gene, namely *KLK5* v.1, v.2 and v.3 (accession numbers: NM_012427.4, NM_001077491.1, and NM_001077492.1, respectively) are currently known (Fig. 3). All three annotated transcripts share a common open reading frame (ORF) and produce the same protein. The coding sequence of all three *KLK5* transcripts is composed of five exons. Besides these coding transcripts, a non-coding splice variant (accession number: AY461805.1) with an intron retention between exons 4 and 5 has been previously identified^28^. This alternative splice variant comprises a premature translation termination codon (PTC) and thus represents a nonsense-mediated mRNA decay (NMD) candidate (Fig. 3).

Bioinformatic analysis confirmed all known splice junctions of the previously annotated *KLK5* transcripts with high coverage, supporting the existence of all annotated transcripts in the pool of human cell lines used in the current study. Moreover, novel alternative splicing events were detected in a few sequencing reads of the obtained FASTQ file, supporting the existence of alternative transcripts with a low frequency. First, we identified a novel splice junction between exons 2 and 6, which revealed the existence of a novel *KLK5* transcript (*KLK5* v.4, with accession number: KX571229). This alternative splice variant lacks an ORF, as it contains a PTC and is therefore an NMD candidate. Moreover, due to its short length, the entire sequence of this splice variant appeared in single reads of the FASTQ file (Fig. 2).

We also detected a novel exon of 48 nucleotides (nt), located between the annotated exons 2 and 3 of the *KLK5* gene (Fig. 3). This exon is exclusively spliced to exons 2 and 3, as no splice junctions between this exon and other ones was found. The sequence of this exon is 5′-GTTCAAGTGATTCTCGTGCCTCCACCTCCTGAGTAGCTGGGATTACAG-3′. RT-PCR using a forward primer that was specific for the splice junction between this novel exon and annotated exon 3 (N/3F) along with another specific reverse primer (5/6R) generated an amplicon of 679 bp, corresponding to *KLK5* v.5 (accession number: KX571230). This variant is predicted to encode an N-truncated KLK5 protein isoform, consisting of 219 amino acid (aa) residues.

In addition, a 5′ extension of exon 6 was identified, being 81 nt in length and having the following sequence: 5′-GCATCTCTGGGTCTCTCATGTCTCCTTCTGCCCACTTTGCCACATCTCTGCCTCTCTCATGCCCCCCTTTCTCTCCTGCAG-3′. In all reads, the extended exon 6 was found to be spliced to exon 5. RT-PCR with the junction-specific primers N/3F and 5/6extR (Fig. 3) uncovered a new transcript comprising both the aforementioned novel exon and the 5′ extension of annotated exon 6. This transcript, named *KLK5* v.6 (accession number: KX571231) is predicted to encode a new polypeptide with the same C-terminus, compared to the other KLK5 isoforms. The sequence of *KLK5* v.5 and the one of v.6 were also validated with Sanger sequencing (see Supplementary Info).

### Novel *KLK6* transcripts

**Figure 4:**
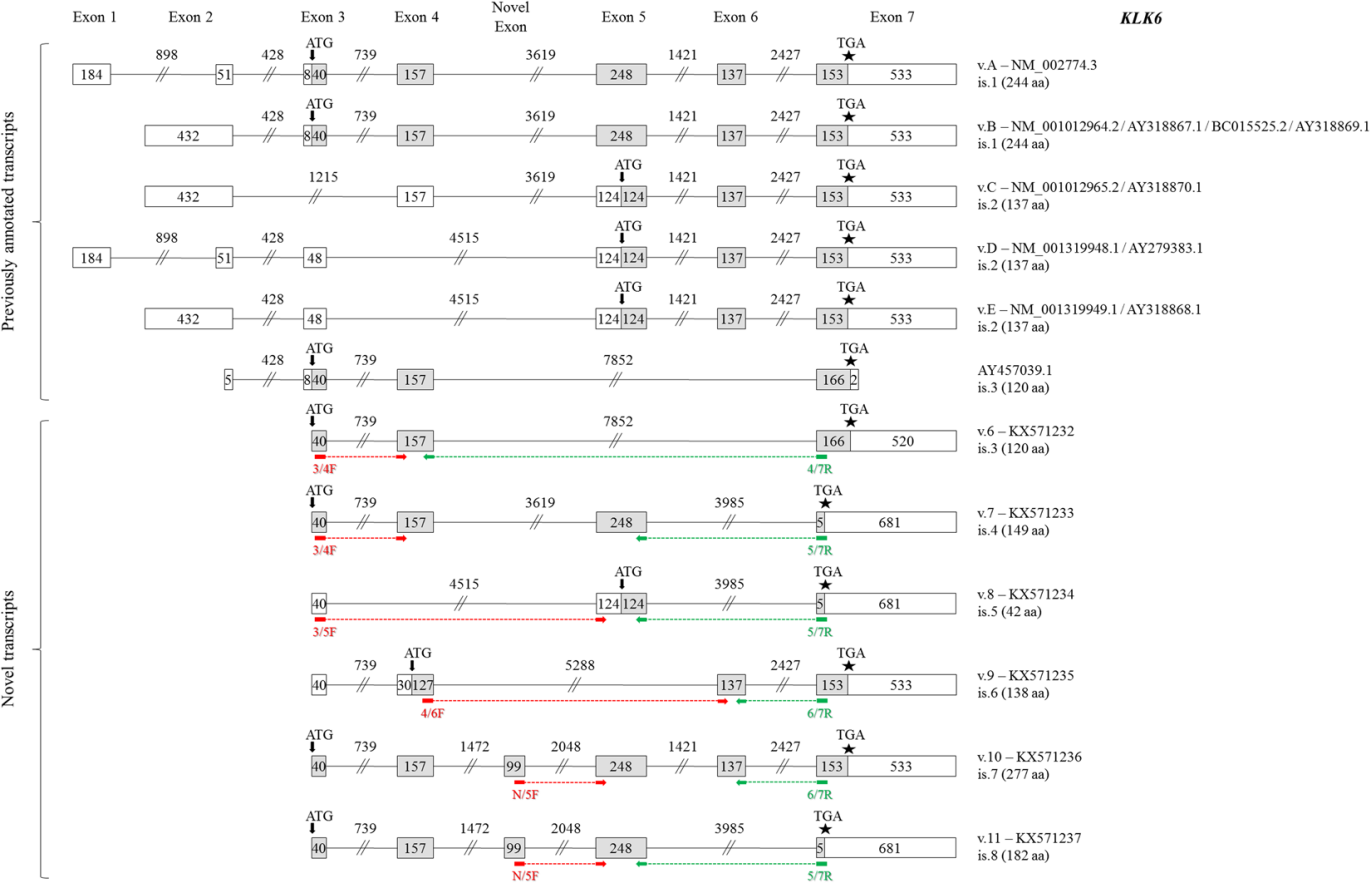
Detailed structure of the *KLK6* splice variants and their respective protein isoforms. Exons are depicted as boxes and introns as lines; gray and white boxes represent coding and non-coding exons, respectively. Numbers inside boxes and above lines indicate the length of each exon or intron in nucleotides. Arrows (↓) show the position of the ATG codon, asterisks (*) indicate the position of the stop codon, and question marks (?) represent an undetermined 5′ UTR. For each new transcript (*KLK6* v.6 – v.11), the splice variant number, the GenBank^®^ accession number (KX571232 – KX571237, respectively) and the protein isoform number (only for splice variants that are predicted to be coding transcripts) are shown next to each transcript. The target regions of the forward (red color) and reverse (green color) variant-specific primers used for expression analysis in nested RT-PCR are also demonstrated.

Five protein-coding transcripts of the *KLK6* gene have been annotated so far (Fig. 4) and are designated as *KLK6* variants A, B, C, D, and E (accession numbers: NM_002774.3, NM_001012964.2, NM_001012965.2, NM_001319948.1, and NM_001319949.1, respectively). Splice variants A and B encode the classical, full-length protein and differ only in their 5′ UTR, whereas splice variants C, D, and E encode an N-truncated polypeptide lacking the signal peptide of the classical KLK6 preproprotein. Additionally, a sixth transcript (accession number: AY457039.1) lacking two internal exons (5 and 6) and utilizing a different translation stop codon has been published^28^. The resulting 120-aa preproprotein has a different C-terminus, compared to the aforementioned protein isoforms (Fig. 4). This protein isoform is also predicted to be encoded by the newly discovered *KLK6* v.6 (accession number: KX571232). The entire coding sequence of this splice variant was directly revealed by single reads of the FASTQ file (Fig. 2), as the coding region is only 363 nt long.

Besides this transcript, our approach led to the discovery of another five *KLK6* splice variants (Fig. 4), the sequences of which were validated using Sanger sequencing (see Supplementary Info). The transcript v.7 (accession number: KX571233) lacks exon 6 and exploits a distinct translation stop codon lying 148 nt upstream of the already annotate one, thus producing a C-truncated protein isoform. The transcript v.8 (accession number: KX571234) is similar to the already annotated variants D and E, but lacks exon 6 compared to these variants; yet, it has an ORF of 129 nt. The transcript v.9 (accession number: KX571235) lacks exon 5, exploits a different translation initiation (ATG) codon, but has the same 3′ UTR as the aforementioned five protein-coding splice variants that are currently known. The respective protein isoform has the same C-terminus as the classical preproprotein, but possesses a different N-terminus and hence lacks the signal peptide.

In addition to the new splice junctions just described (Fig. 4), we discovered a novel exon of 99 nt, located between the annotated exons 4 and 5 of the *KLK6* gene. This exon is exclusively spliced to exons 4 and 5, as no splice junctions between this exon and other ones was detected. The sequence of this newly discovered exon is 5′-AGCACCTGCTAAGTGCCACATTCGAGTT GGGCAGTGGAGATTCAGCAATGGATGGGACACACACGTCATCCCTGCCCTCGGGAGCACAAGGACAGAAAG-3′. RT-PCR using a forward primer that was specific for the splice junction between this novel exon and annotated exon 5 (N/5F) along with another specific reverse primer (6/7R) generated an amplicon of 413 bp, corresponding to *KLK6* v.10 (accession number: KX571236). This variant is predicted to encode a protein isoform of 277 aa, thus being larger than the classical one, as it comprises an additional internal peptide. Similarly, RT-PCR using the same forward primer (N/5F) as previously along with another junction-specific reverse primer (5/7R) generated an amplicon of 276 bp, corresponding to *KLK6* v.11 (accession number: KX571237). This transcript is predicted to encode a polypeptide with a C-terminus similar to the one of protein isoforms 4 and 5, which are encoded by v.7 and v.8, respectively (Fig. 4).

### Novel *KLK7* transcripts

**Figure 5:**
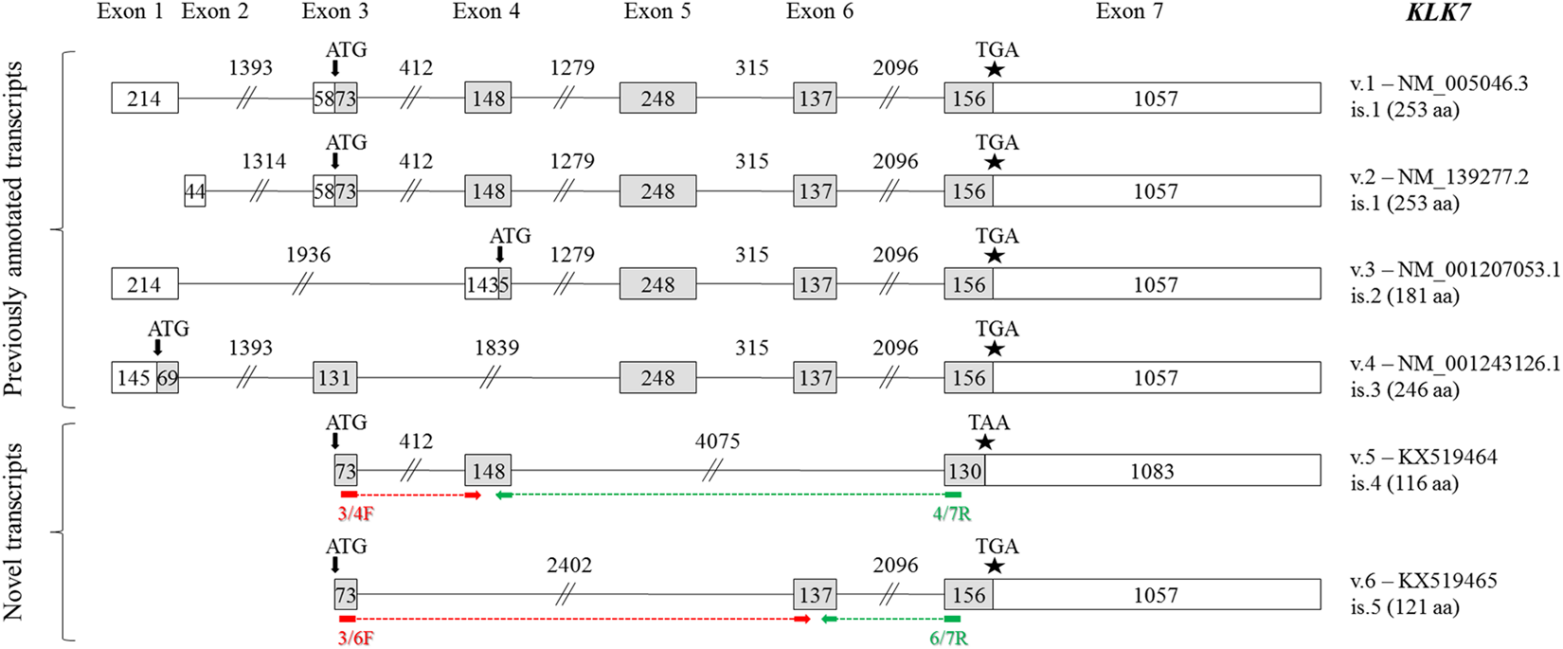
Detailed structure of the *KLK7* splice variants and their respective protein isoforms. Exons are depicted as boxes and introns as lines; gray and white boxes represent coding and non-coding exons, respectively. Numbers inside boxes and above lines indicate the length of each exon or intron in nucleotides. Arrows (↓) show the position of the ATG codon, asterisks (*) indicate the position of the stop codon, and question marks (?) represent an undetermined 5′ UTR. For each new transcript (*KLK7* v.5 and v.6), the splice variant number, the GenBank^®^ accession number (KX519464 and KX519465, respectively) and the protein isoform number (only for splice variants that are predicted to be coding transcripts) are shown next to each transcript. The target regions of the forward (red color) and reverse (green color) variant-specific primers used for expression analysis in nested RT-PCR are also demonstrated.

Four transcripts of the *KLK7* gene have previously been characterized (Fig. 5). These transcripts, namely *KLK7* v.1, v.2, v.3, and v.4 (accession numbers: NM_005046.3, NM_139277.2, NM_001207053.1, and NM_001243126.1, respectively) are protein-coding and share the same translation stop codon and 3′ UTR. The respective KLK7 protein isoforms differ in their C-terminus, whereas *KLK7* v.1 and v.2 use the same ORF.

Besides experimentally validating all these previously annotated transcripts with high NGS coverage, we also discovered two novel alternative splice variants, *KLK7* v.5 and v.6 (Fig. 5), represented by only few sequencing reads in the FASTQ file (Fig. 2). *KLK7* v.5 (accession number: KX519464), which lacks exons 5 and 6, is predicted to utilize the same ATG codon as v.1 and v.2, but its ORF is shifted afterwards; thus, it has a different translation stop codon, lying 26 nt upstream of the already annotate one. As a result, the respective predicted KLK7 protein isoform (is.4) is truncated at its C-terminus. On the other hand, *KLK6* v.5 (accession number: KX519465), which lacks exons 4 and 5, is predicted to utilize the same ATG and stop codon as v.1 and v.2, but the resulting protein isoform is shorter again (121 aa), missing a large internal peptide segment, as compared to KLK7 isoforms 1 and 2 (Fig. 5).

### Novel *KLK8* transcripts

**Figure 6:**
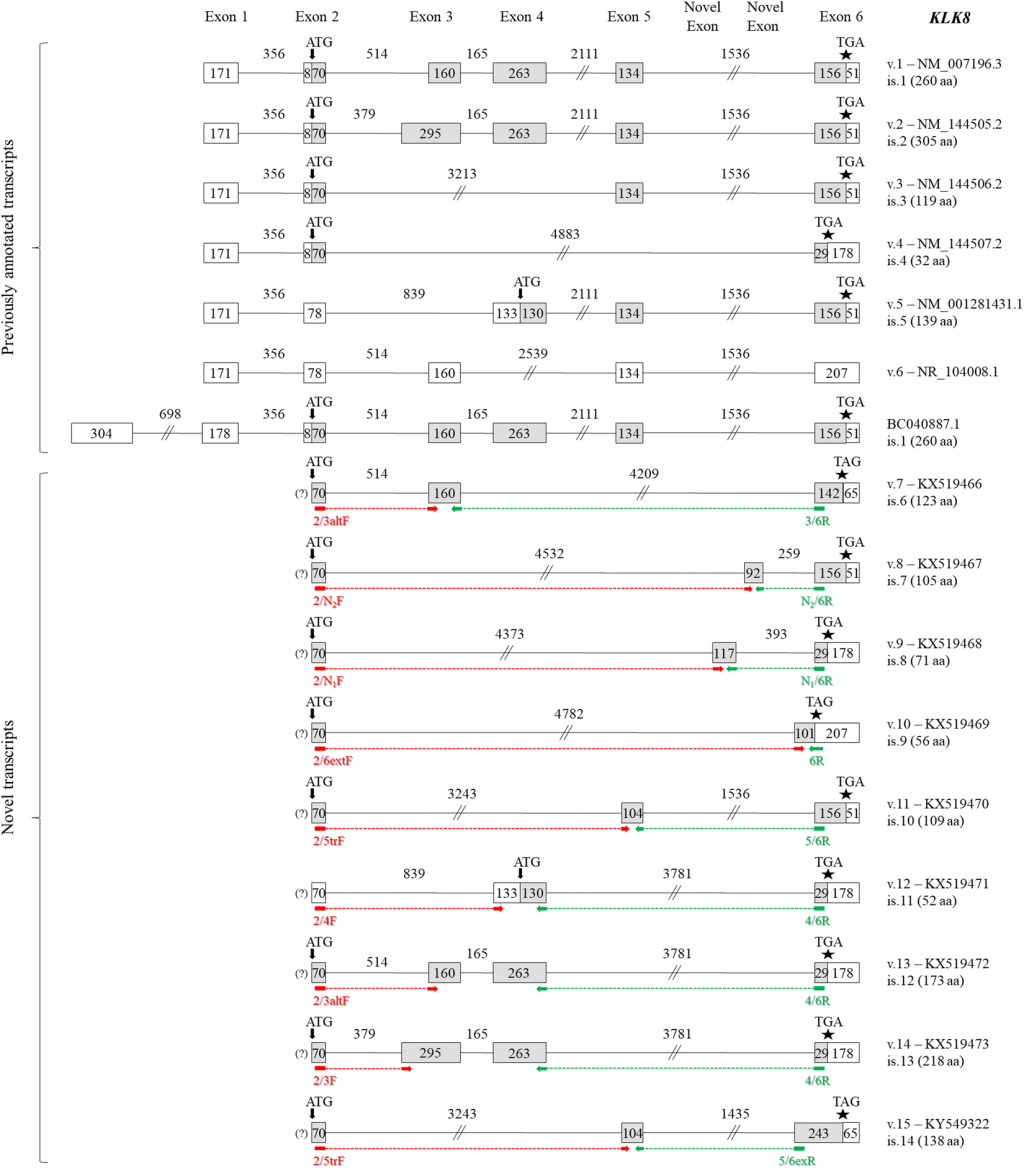
Detailed structure of the *KLK8* splice variants and their respective protein isoforms. Exons are depicted as boxes and introns as lines; gray and white boxes represent coding and non-coding exons, respectively. Numbers inside boxes and above lines indicate the length of each exon or intron in nucleotides. Arrows (↓) show the position of the ATG codon, asterisks (*) indicate the position of the stop codon, and question marks (?) represent an undetermined 5′ UTR. For each new transcript (*KLK8* v.7 – v.15), the splice variant number, the GenBank^®^ accession number (KX519466 – KX519473, respectively) and the protein isoform number (only for splice variants that are predicted to be coding transcripts) are shown next to each transcript. The target regions of the forward (red color) and reverse (green color) variant-specific primers used for expression analysis in nested RT-PCR are also demonstrated.

The *KLK8* main transcripts (v.1 and v.2) consist of five coding exons and four intervening introns (Fig. 6). In addition, exon 3 uses two different 5′ splice sites, and consequently the length of this exon is either 295 nt or 160 nt. Alternative splicing events in the *KLK8* pre-mRNA generate five protein-coding mRNAs (accession numbers: NM_007196.3, NM_144505.2, NM_144506.2, NM_144507.2, and NM_001281431.1, respectively) and one non-coding RNA (NR_104008.1). In our study, bioinformatic analysis and visualization of the NGS data using IGV (see Supplementary Fig. 2) uncovered new splice sites of the already known exons – resulting in a 5′-extended exon 6 and a 5′-trunctated exon 5 – as well as new splicing events between the already known splice sites of the coding exons. Moreover, we identified two novel exons between exons 5 and 6.

The entire coding sequence of six splice variants (*KLK8* v.7 – v.11, and v.15) was directly revealed by single reads of the FASTQ file (Fig. 2). In more detail, *KLK8* v.7 (accession number: KX519466) lacks exons 4 and 5, is predicted to utilize the same ATG codon as v.1 – v.4, but its ORF is shifted after its 160-bp exon 3; thus, it has a different translation stop codon, lying 14 nt upstream of the already annotate one. Therefore, the resulting preproprotein (is.6) of 123 aa has a different C-terminus, compared to all previously annotated KLK8 protein isoforms (Fig. 6). Moreover, *KLK8* v.8, v.9, and v.10 (accession numbers: KX519467, KX519468, and KX519469, respectively) do not comprise exons 3, 4, and 5, but the first two of them contain one of the two novel exons. The first one of these exons (117 nt), located at a distance of 393 nt from the 5′ splice site of exon 6, has the following sequence: 5′-GGTCTCGCTCTGTCCCCCAAGCTGGAATGCAGTGGCACAATCTTGGCTCACTGCA GCCTCTGCCTCCCAGTTCAAGCAATTCTCCCACCTCAGCCTCCCGAGTAGCTGTGATTACAG-3′. The second novel exon (92 nt), also located upstream of exon 6 at a distance of 259 nt, has the following sequence: 5′-AGACAGGGATTCACCATGTTGGCCAGGCTGGTCTGG AACGCCTGACTTCAAGTGATCTACCGCCTCGGCCTCCCAAAGTGCCGGGATTGCAG-3′. *KLK8* v.8 contains the aforementioned novel exon of 92 nt and is predicted to encode a polypeptide (is.7) of 105 aa including the signal peptide, while *KLK8* v.9 contains the aforementioned novel exon of 117 nt and is predicted to encode a short polypeptide (is.8) of 71 aa, having the same C-terminus as the previously annotated is.4, which is encoded by v.4. On the other hand, exon 2 of *KLK8* v.10 is directly spliced to a new 5′ splice site of exon 6, which is located 101 nt upstream of the main one. The predicted preproprotein (is.9) resulting from the translation of this transcript consists of 56 aa and possesses a distinct C-terminus, compared to all other KLK8 protein isoforms. With regard to *KLK8* v.11 (accession number: KX519470), this splice variant contains a 5′-trunctated exon 5 (104 nt), which is spliced to exons 2 and 6, similarly to *KLK8* v.3. The protein isoform that is predicted to be encoded by v.11, is.10, lacks an internal peptide of 10 aa, compared to is.3.

On the other hand, the next three novel splice variants (*KLK8* v.12 – v.14) lack exon 5 and share the same translation stop codon, which is located 127 nt upstream of the main one (Fig. 6). The sequences of these transcripts were also validated using Sanger sequencing (see Supplementary Info). In particular, *KLK8* v.12 (accession number: KX519471) utilizes a different ATG codon and most likely encodes a protein isoform (is.11) having the same C-terminus as KLK8 is.4 and is.8, still possessing a different N-terminus and hence lacking the signal peptide, similarly to KLK8 is.5. The structure of *KLK8* v.13 and v.14 (accession numbers: KX519472 and KX519473, respectively) resembles that one of v.1 and v.2, with the sole exception of the absence of exon 5 in the former two transcripts. Their translation is expected to produce two KLK8 isoforms having the same C-terminus as the aforementioned is.11, encoded by *KLK8* v.12, yet shorter by 87 aa than KLK8 is.1 and is.2, respectively (Fig. 6). Finally, *KLK8* v.15 (accession number: KX519472) resembles to *KLK8* v.11 as it contains the same 5′-trunctated exon 5 (104 nt) and exploits the same ORF; this exon is spliced to exon 2 and a 5′-extended exon 6, similar to the last exon of *KLK8* v.10. The KLK8 protein isoform that is predicted to be encoded by v.15, is.14, possesses an additional internal peptide of 29 aa, compared to is.10.

### Novel *KLK9* transcripts

**Figure 7:**
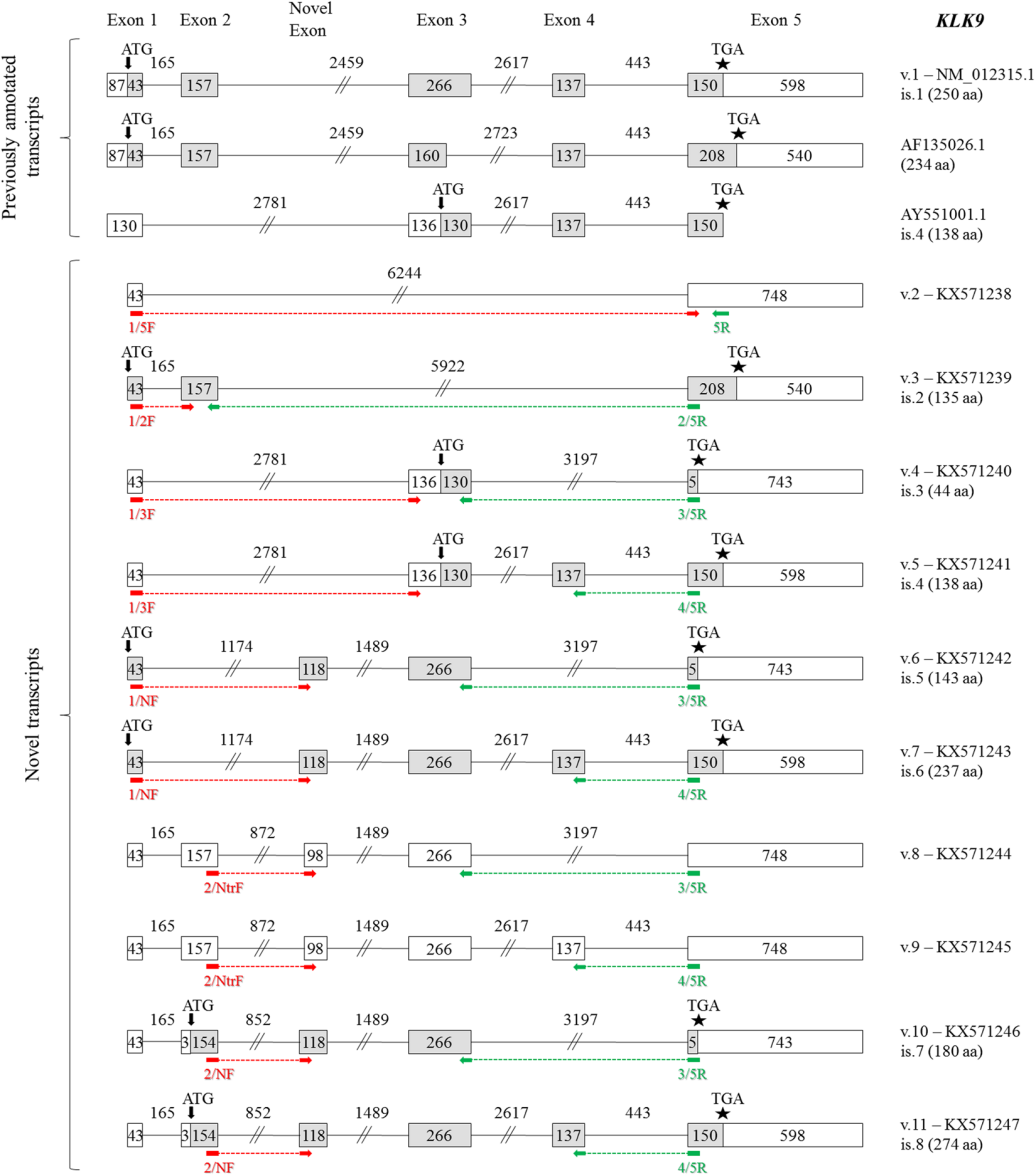
Detailed structure of the *KLK9* splice variants and their respective protein isoforms. Exons are depicted as boxes and introns as lines; gray and white boxes represent coding and non-coding exons, respectively. Numbers inside boxes and above lines indicate the length of each exon or intron in nucleotides. Arrows (↓) show the position of the ATG codon, asterisks (*) indicate the position of the stop codon, and question marks (?) represent an undetermined 5′ UTR. For each new transcript (*KLK9* v.2 – v.11), the splice variant number, the GenBank^®^ accession number (KX571238 – KX571247, respectively) and the protein isoform number (only for splice variants that are predicted to be coding transcripts) are shown next to each transcript. The target regions of the forward (red color) and reverse (green color) variant-specific primers used for expression analysis in nested RT-PCR are also demonstrated.

The classical transcript of the *KLK9* gene (accession number: NM_012315.1) is composed of five coding exons (Fig. 7). Two other *KLK9* protein-coding splice variants have been deposited in GenBank^®^; the first one (accession number: AF135026.1) contains an alternative exon 3 that is 3′-truncated by 106 nt, resulting in a different translation stop codon, whereas the second one (accession number: AY551001.1) lacks exon 2 and uses a different translation start codon. The respective protein isoform (is.4) has the same C-terminus as the classical preproprotein, but possesses a different N-terminus and hence lacks the signal peptide.

Our study revealed another ten splice variants of the *KLK9* gene, most of which can be considered as protein-coding transcripts, while three out of ten distinct transcripts – namely *KLK9* v.2, v.8 and v.9 (accession numbers: KX571238, KX571244, and KX571245, respectively) – contain a PTC and are therefore NMD candidates (Fig. 7). The *KLK9* v.2 transcript contains only exons 1 and 5; its unique splice junction was directly revealed by single reads of the FASTQ file (Fig. 2). Similarly, single reads contained the entice coding sequence of the *KLK9* v.3 transcript (accession number: KX571239), a coding splice variant lacking exons 3 and 4 (Fig. 2). The respective predicted preprotein lacks a large internal peptide segment, compared to the KLK9 isoform of 234 aa, encoded by the previously identified transcript with accession number: AF135026.1 (Fig. 7). On the other hand, *KLK9* v.4 and v.5 use a different ATG codon, located in exon 3. These two predicted KLK9 isoforms (is.3 and is.4) lack the signal peptide of the classical KLK9 preproprotein. Moreover, particularly *KLK9* v.4 utilizes a distinct translation stop codon and is predicted to encode a 3′-truncated KLK9 isoform of 44 aa.

Besides the aforementioned new splice sites of the already known exons of the *KLK9* gene, bioinformatic analysis and visualization of the NGS data using IGV (see Supplementary Fig. 2) uncovered two overlapping novel exons preceding exon 3 by 1489 nt from its 5′ splice site. The first one of these exons (118 nt) has the following sequence: 5′-AGTCTCCCTCTATCTCCCAG GCTGGAGTGCAGTGGCACAATCTCAGCTCACTGCAACCTCTGCCTCTCGGGTTCAAGCAATTCTCCTGCCTCAGCCTCCTGAGTAGCTGGGATTACAG-3′, while the other one has a 5′ truncation of 20 nt, resulting in the following sequence: 5′-GCTGGAGTGCAGTGGCACA ATCTCAGCTCACTGCAACCTCTGCCTCTCGGGTTCAAGCAATTCTCCTGCCTCAGCCTCCTGAGTAGCTGGGATTACAG-3′. According to our data, the novel exon of 98 nt is exclusively spliced to exons 2 and 3; this exon is included in *KLK9* v.8 and v.9 (Fig. 7). On the contrary, the exon of 118 nt can be preceded either by exon 1 or exon 2. Thus, *KLK9* v.6 and v.7 (accession numbers: KX571242 and KX571243, respectively), both of which lack exon 2, are likely to encode preproteins with the same signal peptide as the classical KLK9 isoform (is.1), in contrast with *KLK9* v.10 and v.11 (accession numbers: KX571246 and KX571247, respectively), which make use of an alternative ATG codon, thus producing two respective protein isoforms with a distinct N-terminus, not containing any signal peptide. Lastly, *KLK9* v.4 – v.11 sequences were validated by Sanger sequencing (see Supplementary Info).

### Expression analysis of the novel *KLK5 – KLK9* transcripts in established cell lines

**Figure 8:**
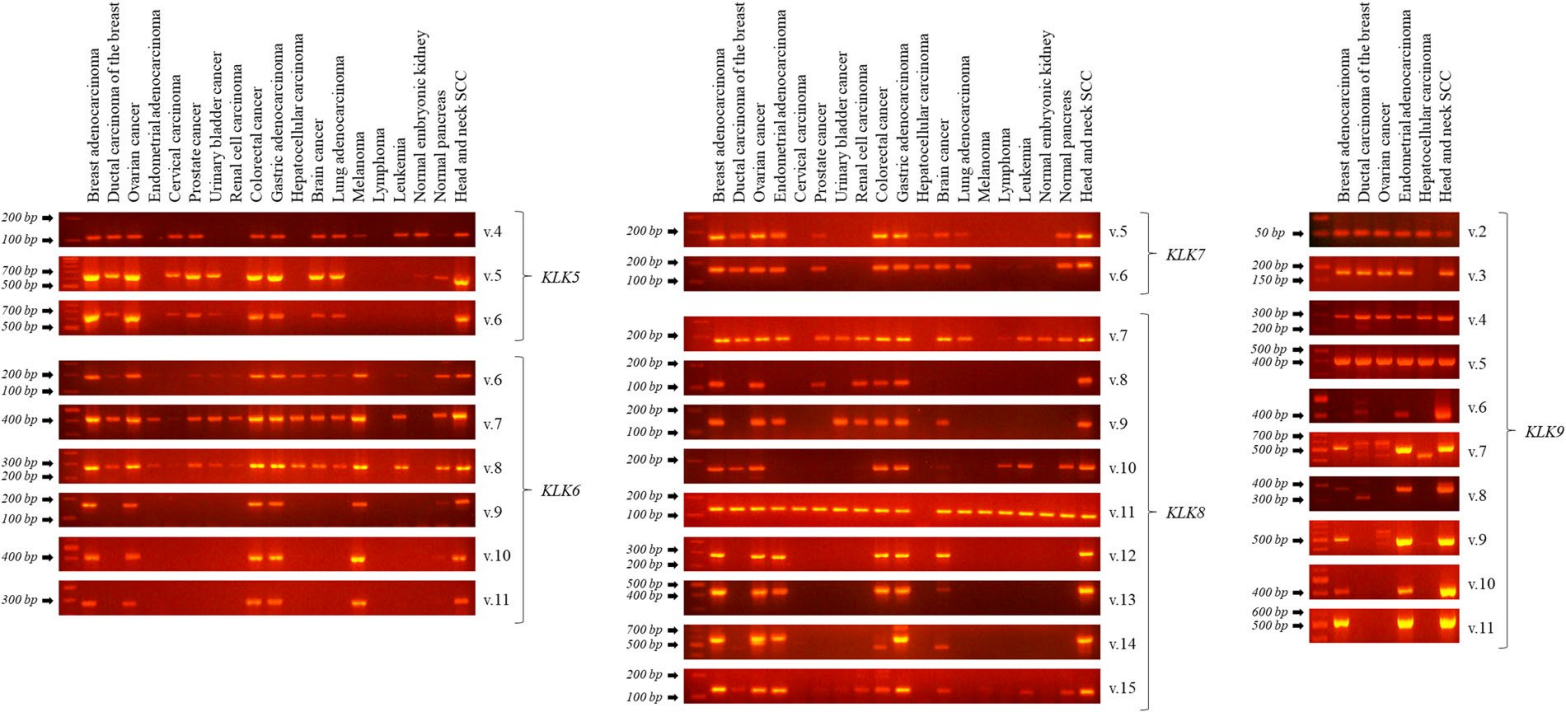
Expression analysis of each novel splice variant in a panel of cDNAs corresponding to distinct cancerous and normal human tissues. cDNAs derived from established cell lines were mixed to generate cDNA pools, each one representing a different human tissue. Regarding *KLK9*, only tissues with detectable expression of at least one novel splice variant are shown. The original gel pictures are shown in Supplementary Info.

Following the identification of all thirty novel transcripts, the expression profiling of each splice variant in a panel of cDNAs corresponding to distinct cancerous human tissues (breast adenocarcinoma, ductal carcinoma of the breast, ovarian cancer, endometrial adenocarcinoma, cervical carcinoma, prostate cancer, urinary bladder cancer, renal cell carcinoma, colorectal cancer, gastric adenocarcinoma, hepatocellular carcinoma, brain cancer, lung adenocarcinoma, melanoma, lymphoma, leukemia, and head and neck squamous cell carcinoma) as well as normal embryonic kidney and normal pancreas was carried out via nested RT-PCR using variant-specific pairs of primers (Fig. 8). Nested RT-PCR amplicons corresponding to transcripts not covered by single NGS reads were validated with Sanger sequencing (see Supplementary Info).

## Discussion

In eukaryotic organisms, the primary transcripts of the protein-coding genes, namely pre-mRNAs, encode both intronic and exonic sequences. All pre-mRNAs are then processed by the spliceosome and their intronic sequences are spliced out in a highly orchestrated fashion to generate the respective mRNAs^29^. Consequently, it can be easily assumed that RNA splicing is a multistep biological procedure involving complex and finely tuned interactions between RNA and DNA, RNA and RNA, or RNA and proteins^30^. Although alternative splicing is a process that is characterized by strict regulation, specific regulatory malfunctions may lead to alterations in splicing patterns causing unfavorable effects in the cellular homeostasis. In detail, several splicing alterations have been found to be associated with cancer, as particular alternatively spliced transcripts can affect the transformation, motility, and metastasis of tumor cells^31–33^.

Alternative splicing events occur on the primary transcripts of the overwhelming majority of human genes. In particular, alternative splicing of cancer-related genes may affect cell cycle control, proliferation, apoptosis, angiogenesis, as well as invasion and metastasis^33^. Despite the fact that in most cases the relationship between specific splicing events and the etiology of cancer is not yet fully understood, novel alternatively spliced transcripts appear as new candidate biomarkers for diagnostic and/or prognostic purposes and as targets for therapeutic strategies^34–40^. The gene family of *KLK*s constitutes a prominent example, as all its members have been shown to be subjected to alternative splicing, generating alternative transcripts some of which have a significant interest, from a clinical perspective. KLKs constitute a family of trypsin- or chymotrypsin-like serine peptidases, the expression of which is hormonally regulated in several tissues^41^.

In the current study, 30 novel alternative transcripts of the human *KLK5*, *KLK6*, *KLK7*, *KLK8*, and *KLK9* genes were discovered and their sequences were deposited in GenBank^®^. Although all these newly discovered transcripts are 5′-partial, as the respective sequences start at the target region of the forward gene-specific primer that was used in the 3′ RACE PCR and hence their 5′ UTR remains undetermined, most of them have an ORF and are predicted to encode novel protein isoforms. Only a few novel transcripts of these five members of the *KLK* gene family may represent NMD candidates. NMD is elicited by a PTC residing 5′ to a limit of approximately 50 nt upstream of the last exon junction, whereas mRNAs with a translation termination codon 3′ to this limit are usually stable^42–44^. However, whether these novel transcripts that are predicted to encode new isoforms of kallikrein-related peptidases are translated into functional proteins remains to be explored.

Most *KLKs* are implicated in a wide range of physiological processes^45^; however, there are also isoforms with no proteolytic activity, as they are missing the catalytic histidine, aspartic acid and/or serine residues that make up the catalytic triad^28,46^. The canonical function(s) of such KLK isoforms is yet unknown. Furthermore, the exact mechanism(s) by which KLKs exert their functions, their levels of activity and their putative endogenous targets *in vivo* have recently emerged. It has been clearly demonstrated that active KLKs secreted by tumors and inflamed tissues show hormone-like properties and that their proteolytic activity is strictly controlled by proteinase inhibitors that are present in cancer-derived fluids^47^. Moreover, several reports suggest a potential function of KLKs in the processing of hormones. For instance, many KLK family members, including KLK5, KLK6, KLK7, and KLK8, are expressed in the pituitary gland, co-localize with the human growth hormone, and are likely to participate in the proteolytic processing of this hormone to its functional fragments^48^. Therefore, it would be rather interesting to perform expression analysis of these novel *KLK5*, *KLK6*, *KLK7*, and *KLK8* transcripts in the pituitary gland.

Many studies have uncovered so far the potential of *KLK*s as promising cancer biomarkers^49,50^. The mRNA expression status of the *KLK5*, *KLK6*, *KLK7*, *KLK8* and *KLK9* has been extensively studied in cancer. For example, *KLK6* mRNA overexpression is associated with lymph node invasion and advanced clinical stage of gastric cancer patients, thus predicting poor prognosis for these patients^51–53^. Similarly, *KLK6* mRNA overexpression in colorectal cancer is associated with serosal invasion, liver metastasis, and advanced Dukes’ stage, again being an unfavorable predictor of overall survival^54,55^. Moreover, particular transcripts have been shown to possess clinical value in some malignancies, such as the short *KLK5* and long *KLK7* transcripts in epithelial-derived serous carcinomas^56^, or the full-length *KLK6* and *KLK11* transcripts in colorectal cancer^55,57^. The alternatively spliced *KLK4* gene constitutes another example, as its classical transcript has been shown to predict short-term relapse both in colorectal adenocarcinoma^58^ and laryngeal squamous cell carcinoma^59^. Therefore, last but not least, it should be mentioned that the newly discovered transcripts presented in this study may constitute *per se* valuable disease biomarkers, especially regarding particular human malignancies. Undoubtedly, their expression patterns in both cancerous and normal tissue specimens merit further investigation.

Due to their multiple roles in cellular homeostasis and physiology, their involvement in key signaling pathways, the wide range of tissues in which they are expressed or secreted, but mostly due to their high potential as biomarkers that could be used for diagnostic and prognostic purposes in several human malignancies, KLKs have attracted much of the attention of the scientific community during the last decades. For all these reasons, deciphering the complexity of the transcripts originating from the kallikrein locus is very important, and NGS technology will certainly be a key player for this accomplishment in the near future.

## Methods

### Human cell line culture

The panel of 55 human cell lines used in this study included: MCF-7, SK-BR-3, BT-20, MDA-MB-231, MDA-MB-468 (breast adenocarcinoma), BT-474, T-47D, ZR-75-1 (ductal carcinoma of the breast), OVCAR-3, SK-OV-3, ES-2, MDAH-2774 (ovarian cancer), Ishikawa, SK-UT-1B (endometrial adenocarcinoma), HeLa, SiHa (cervical carcinoma), PC-3, DU 145, LNCaP (prostate cancer) T24, RT4 (urinary bladder cancer), ACHN, 786-O, Caki-1 (renal cell carcinoma), Caco-2, DLD-1, HT-29, HCT 116, SW 620, COLO 205, RKO (colorectal cancer), AGS (gastric adenocarcinoma), Hep G2, HuH-7 (hepatocellular carcinoma), U-87 MG, U-251 MG, D54, H4, SH-SY5Y (brain cancer), A549 (lung adenocarcinoma), FM3, MDA-MB-435S (melanoma), Raji, Daudi, U-937 (lymphoma), K-562, HL-60, Jurkat, REC-1, SU-DHL-1, GRANTA-519 (leukemia), HEK293 (normal embryonic kidney), 1.2B4 (normal pancreas), and BB49-SCCHN, CAL-33 (head and neck squamous cell carcinoma). All the above cell lines were propagated based on the American Type Culture Collection instructions.

### Total RNA extraction and reverse transcription

Total RNA was extracted from each cell line using the TRIzol Reagent (Ambion™, Thermo Fisher Scientific Inc., Waltham, MA, USA), diluted in THE RNA Storage Solution (Ambion™), and stored at −80 °C until further use. The assessment of purity and concentration of each RNA sample was performed spectrophotometrically at 260 and 280 nm. Then, first-strand cDNA synthesis was performed based on the manufacturer’s guidelines, in reaction volumes of 20 μL, using 5 μg of total RNA from each cell line, SuperScript II Reverse Transcriptase (Invitrogen™, Thermo Fisher Scientific Inc.) and an oligo-dT–adapter as primer (5′-GCGAGCACAGAATTAATACGACTCACTATAGGTTTTTTTTTTTTVN-3′, where V = G, A, C, and N = G, A, T, C).

### Molecular cloning of novel splice variants using 3′ RACE

After cDNA synthesis, 3′ RACE was performed for the molecular cloning of *KLK5*, *KLK6*, *KLK7*, *KLK8*, and *KLK9* transcripts. Briefly, a forward specific primer [F_(ATG)_] for each target gene was designed to target the region of the annotated translation start codon. Primer sequences are presented on Supplementary Table 1. Each forward gene-specific primer was used along with a universal reverse primer (R_(Outer)_) that was designed to anneal on the oligo-dT–adapter primer sequence. 3′ RACE was carried out in a total volume of 50 μL containing MgCl_2_-free KAPA Taq Buffer C (Kapa Biosystems Inc., Woburn, MA, USA), 1.5 mM MgCl_2_, 0.2 mM dNTPs, 50 pmol of each primer, and 2 units of KAPA Taq DNA Polymerase (Kapa Biosystems Inc.), in a Veriti 96-Well Fast Thermal Cycler (Applied Biosystems™, Thermo Fisher Scientific Inc.), under the following cycling conditions: a denaturation step at 95 °C for 3 min, followed by 35 cycles of 95 °C for 30 sec, 60 °C for 30 sec, 72 °C for 2 min, and a final extension step at 72 °C for 5 min. 3′ RACE products were appropriately diluted in nuclease-free water and used as templates for 3′ RACE nested PCR, which was carried out using an internal gene-specific forward primer [F_(Nested)_] and a second universal reverse primer (R_(Inner)_). The purpose of 3′ RACE nested PCR was to increase sensitivity and specificity for the targeted genes, as well as the 3′ RACE yield.

Next, 3′ RACE nested PCR products were cleaned-up using the NucleoSpin Gel and PCR Clean-up kit (Macherey-Nagel GmbH & Co. KG, Duren, Germany), following the manufacturer’s guidelines. After the clean-up workflow, concentration and purity of purified PCR products were assessed spectrophotometrically at 260 and 280 nm, before being stored at −20 °C until further use.

### NGS library construction and quantification

A volume of 10 μL of each purified 3′ RACE nested PCR product was mixed in a final PCR product sample. Then, starting from 100 ng of purified PCR product mix, NGS library preparation was carried out with the use of the Ion Xpress™ Plus Fragment Library Kit (Ion Torrent™, Thermo Fisher Scientific Inc.). At the first step of the workflow, Ion Shear™ Plus Reagents (Ion Torrent™) were used for the enzymatic fragmentation of the PCR product mix, followed by purification of the fragmented DNA. Then, adapter ligation, nick-repair and purification of the ligated DNA were performed. The last step of the library construction was the size selection of the unamplified library (200-base-read library) and was accomplished using an E-Gel^®^ SizeSelect™ 2% Agarose Gel (Invitrogen™). The quantification of the size-selected library was performed using the Ion Library TaqMan™ Quantitation Kit (Ion Torrent™) in a ABI 7500 Fast Real-Time PCR System (Applied Biosystems™).

### Template preparation, enrichment, and NGS

The Ion PGM™ Template OT2 200 Kit (Ion Torrent™) was used for the preparation of the NGS template in an Ion OneTouch™ 2 System (Ion Torrent™), according to the manufacturer’s instructions. The quality of the unenriched template-positive Ion Sphere™ Particles (ISPs) was estimated using the Ion Sphere™ Quality Control Kit in a Qubit^®^ 2.0 Fluorometer (Invitrogen™). At the next step, the enrichment of the unenriched templated ISPs was performed using again the Ion PGM™ Template OT2 200 Kit (Ion Torrent™) in an Ion OneTouch ES™ instrument (Ion Torrent™). Finally, NGS based on the semi-conductor sequencing technology was carried out using the Ion PGM™ Sequencing 200 Kit v2 (Ion Torrent™) in an Ion PGM™ System (Ion Torrent™), according to the manufacturer’s instructions.

### Bioinformatic analysis

The Torrent Suite™ Software (Ion Torrent™) was used for basecalling and alignment of raw data to the human genome (hg38). As a result, the generated FASTQ file was uploaded to the publicly available online GALAXY suite of software tools for NGS data analysis (https://galaxyproject.org), which provides a significant number of bioinformatic tools as well as specialized algorithms for the analysis of NGS data. More specifically, TopHat2, a popular aligner for RNA-seq experiments producing sensitive and accurate alignments, was used to align the obtained sequencing reads and to identify novel splice sites with direct mapping to known transcripts^60^. In our analysis, TopHat2 produced several output files, including the “accepted hits” (BAM) file that contains a list of the reads aligned to the reference genome and the “splice junctions” (BED) file that comprises the detected splice junctions. Both BAM and BED files were used for visualization, processing, and analysis of the NGS data. IGV was used for interactive visual exploration of the results contained in the BAM and BED files^61^, uncovering alternative splicing events in read alignments (see Supplementary Fig. 2). IGV enables high performance visualization and thorough examination of regions in the reference genome where the sequencing reads are mapped, and at the same time provides the read coverage of every genomic region.

### Expression analysis of the novel splice variants using nested RT-PCR

In order to perform expression analysis of the novel splice variants, cDNAs derived from cell lines originating from the same tissue were mixed. Thus, we obtained seventeen cDNA pools, namely from breast adenocarcinoma, ductal carcinoma of the breast, ovarian cancer, endometrial adenocarcinoma, cervical carcinoma, prostate cancer, urinary bladder cancer, renal cell carcinoma, colorectal cancer, gastric adenocarcinoma, hepatocellular carcinoma, brain cancer, lung adenocarcinoma, melanoma, lymphoma, leukemia, and head and neck squamous cell carcinoma, whereas two normal tissues (normal embryonic kidney and normal pancreas) were represented by a single cell line cDNA. Next, a specific pair of PCR primers for all transcripts of each gene (*KLK5*, *KLK6*, *KLK7*, *KLK8*, and *KLK9*) was designed using Primer-BLAST (Supplementary Tables 2 and 3) and was used in an RT-PCR exploiting as template each of the nineteen cDNA samples.

RT-PCR was carried out in a total volume of 50 μL containing MgCl_2_-free KAPA Taq Buffer C (Kapa Biosystems Inc.), 1.5 mM MgCl_2_, 0.2 mM dNTPs, 50 pmol of each primer, and 2 units of KAPA Taq DNA Polymerase (Kapa Biosystems Inc.), in a Veriti 96-Well Fast Thermal Cycler (Applied Biosystems™), under the following cycling conditions: a denaturation step at 95 °C for 3 min, followed by 25 cycles of 95 °C for 30 sec, 60 °C for 30 sec, 72 °C for 2 min, and a final extension step at 72 °C for 5 min.

PCR products were appropriately diluted in nuclease-free water and used as templates for nested RT-PCR (30 thermal cycles; elongation step in each cycle: 72 °C for 1 min; same PCR mixture composition as for RT-PCR), yet using a specific pair of PCR primers for each distinct transcript, again designed using Primer-BLAST (Supplementary Tables 2 and 4). Equal volumes of nested RT-PCR products were electrophorized on 2.0–3.0% NuSieve GTG Agarose (Cambrex Bio Science Rockland Inc., Rockland, ME, USA) gels and visualized under UV light by ethidium bromide staining. Bands were appropriately excised and purified using a Gel and PCR Clean-up kit (Macherey-Nagel GmbH & Co. KG), before being subjected to Sanger sequencing in both directions, for amplicon sequence verification.

## Electronic supplementary material

Supplementary Information
