# Supplementary material for: Molecular cloning of novel transcripts of human kallikrein-related peptidases 5, 6, 7, 8 and 9 (KLK5 – KLK9), using Next-generation sequencing
Source: Sci Rep. 2017 Dec 11;7:17299. doi: 10.1038/s41598-017-16269-6 (PMC5725587; doi:10.1038/s41598-017-16269-6)

Molecular cloning of novel transcripts of human kallikrein-related peptidases 5, 6, 7, 8 and 9 (*KLK5 – KLK9*), using Next-generation sequencing

Panagiotis G. Adamopoulos, Christos K. Kontos, and Andreas Scorilas*

Department of Biochemistry and Molecular Biology, National and Kapodistrian University of Athens, Athens 15701, Greece

*ascorilas@biol.uoa.gr

<u>Supplementary Information</u>

Supplementary Tables

Supplementary Table 1. Primers used in 3′ RACE and 3′ RACE nested PCR for the molecular cloning of *KLK5, KLK6, KLK7, KLK8,* and *KLK9* transcripts. $F_{(ATG)}$ is a gene-specific forward primer targeting the region of the annotated translation start codon region, and was used along with a universal reverse primer ($R_{(Outer)}$) in 3′ RACE. $F_{(Nested)}$ is an internal gene-specific forward primer, used in combination with a second universal reverse primer ($R_{(Inner)}$) in 3′ RACE nested PCR. Melting temperature (T_m) was calculated by Primer-BLAST.

**Gene**	**Primers**				
	**Direction**	**Name**	**Sequence (5′→3′)**	**Length (nt)**	**T_m (ºC)**
KLK5	Forward	$F_{(ATG)}$	CATGGCTACAGCAAGACCCC	20	60.8
		$F_{(Nested)}$	GATGTGGGTGCTCTGTGCTC	20	61.0
KLK6		$F_{(ATG)}$	CCATGAAGAAGCTGATGGTGGT	22	60.6
		$F_{(Nested)}$	GGTGCTGAGTCTGATTGCTGC	21	61.9
KLK7		$F_{(ATG)}$	CATGGCAAGATCCCTTCTCCTG	22	60.8
		$F_{(Nested)}$	GCCCCTGCAGATCTTACTGCT	21	62.2
KLK8		$F_{(ATG)}$	CCTCACCATGGGACGCC	17	59.8
		$F_{(Nested)}$	ACCTCGTGCGGCCAAGA	17	61.3
KLK9		$F_{(ATG)}$	ATGAAGCTGGGACTCCTCTGT	21	60.6
		$F_{(Nested)}$	TGCTCTGCTCTCTCTGCTGG	20	61.6
Universal	Reverse	$R_{(Outer)}$	GCGAGCACAGAATTAATACGACT	23	59.2
		$R_{(Inner)}$	AGCACAGAATTAATACGACTCACTATAGG	29	60.7

.

Supplementary Table 2. Primers used in RT-PCR and nested RT-PCR for expression analysis of the novel splice variants of *KLK5*, *KLK6*, *KLK7*, *KLK8*, and *KLK9*. $F_{(ATG)}$ is a gene-specific forward primer (shown in *italics*) targeting the region of the annotated translation start codon region, and was used along with the respective gene-specific reverse primer (shown in *italics*) in RT-PCR. The other variant-specific primers were used in nested RT-PCR. The number(s) appearing in primer names denote the number of each exon, while "/" shows that the primer is designed to target a particular splice junction; "N" indicates a novel exon, "ext" means an extended exon, "tr" demonstrates a truncated exon, and "alt" stands for "alternative exon". Melting temperature (T_m) was calculated by Primer-BLAST.

Gene	Primers				
	**Direction**	**Name**	**Sequence (5′→3′)**	**Length (nt)**	**T_m (ºC)**
KLK5	Forward	*$F_{(ATG)}$*	*CATGGCTACAGCAAGACCCC*	*20*	*60.8*
		2/6F	GGGTCACAGGGTGATTCTGG	20	60.0
		N/3F	AGCTGGGATTACAGAGCATGTT	22	59.8
	Reverse	*6R**	*GTGAACTTGCAGAGGTTCGTGTA*	*23*	*61.0*
		6R	GTAGACACCCGGTCTGTTGG	20	60.0
		5/6R	CCCAGAATCACCCTGGCAG	19	60.1
		5/6extR	ACCCAGAGATGCCTGGCAG	19	61.7
KLK6	Forward	*$F_{(ATG)}$*	*CCATGAAGAAGCTGATGGTGGT*	*22*	*60.6*
		3/4F	TGATTGCTGCAGCCTGGG	18	60.4
		3/5F	TGCTGCAGGAATCTTCAGGT	20	59.3
		4/6F	CACTGCAAAAAACCGTGATTTCC	23	59.8
		N/5F	GCACAAGGACAGAAAGGAATCTTC	24	60.1
	Reverse	*7R*	*GGTGGTCTCCACATACCAGC*	*20*	*60.1*
		4/7R	CCCCAGAATCACCGGTTTTTTG	22	60.3
		5/7R	CCCAGAATCACCCATCTGCTG	21	60.8
		6/7R	CCCAGAATCACCCTGGCAG	19	60.1
KLK7	Forward	*$F_{(ATG)}$*	*CATGGCAAGATCCCTTCTCCTG*	*22*	*60.8*
		3/4F	AGGAGAAGAAGCCCAGGGT	19	59.9
		3/6F	TGCAGGAGAAGAAGTGACCTTTC	23	60.5

	Reverse	*7R*	*GTACCTCTGCACACCAACGGT*	*21*	*62.6*
		4/7R	CCCTGAGTCACCTTCATCTTGC	22	60.9
		6/7R	CCCTGAGTCACCATTGCAGG	20	60.7
KLK8	Forward	*F(ATG)*	*CCTCACCATGGGACGCC*	*17*	*59.8*
		2/3altF	AGCCTGGGCAGGACACTC	18	61.3
		$2/N_2F$	AGCCTGGGCAGAGACAGG	18	61.0
		$2/N_1F$	AGCCTGGGCAGGGTCTC	17	60.7
		2/6extF	CCTGGGCAGGGGACCAAT	18	61.4
		2/5trF	GGGAGCCTGGGCAGAAGTAA	20	61.6
		2/4F	GAGCCTGGGCAGGAAATACA	20	59.7
		2/3F	CCTGGGCAGCGTGTGGAA	18	62.6
	Reverse	*6R**	*GCCAGGTTTGTCGGACCTC*	*19*	*60.7*
		3/6R	TCCAGAATCGCCGGTTTTTTAC	22	59.5
		$N_2/6R$	TCCAGAATCGCCCTGCAAT	19	59.4
		$N_1/6R$	TCCAGAATCGCCCTGTAATCAC	22	60.2
		6R	GGATGTGATGCCCTGGAGTG	20	60.5
		5/6R	TCCAGAATCGCCCTGGCA	18	61.0
		4/6R	TCCAGAATCGCCCTCGGG	18	61.2
		5/6extR	CATGATTGGTCCCCTGGCA	19	59.7
KLK9	Forward	*F(ATG)*	*ATGAAGCTGGGACTCCTCTGT*	*21*	*60.6*
		1/5F	TCTGCTGGCAGGGTGACT	18	60.5
		1/2F	TCTGCTGGCAGGGCATG	17	59.7
		1/3F	CTGCTGGCAGGTATCTGTGG	20	60.5
		1/NF	TGCTGGCAGAGTCTCCCTC	19	61.0
		2/NtrF	AAGCCGCTGGAGTGCAGT	19	62.0
		2/NF	CTGCCGCAAGCCAGTCTC	18	61.0
	Reverse	*5R**	*GGCATACGCTGGTGTAGACTG*	*21*	*60.8*
		5R	GGTTCCATTGCAAACCAGGG	20	59.7
		2/5R	CAGAGTCACCGGCTTGCG	18	61.1
		3/5R	CCAGAGTCACCCCTTGGGG	19	61.6
		4/5R	AGAGTCACCCTGGCAGGA	18	59.5

Supplementary Table 3. Primer pairs used in RT-PCR for expression analysis of each novel splice variant in a series of established cell lines, originating from several cancerous and/or normal human tissues. The forward primer $F_{(ATG)}$ for each gene encompasses the region of the annotated translation start codon region and was used along with the respective gene-specific reverse primer in RT-PCR to amplify all alternatively spliced transcripts of the respective gene.

Gene	Splice variant	Primer name		Amplicon size (bp)
		Forward	Reverse	
KLK5	v.4	$F_{(ATG)}$	6R*	193
	v.5			894
	v.6			975
KLK6	v.6	$F_{(ATG)}$	7R	236
	v.7			484
	v.8			327
	v.9			373
	v.10			720
	v.11			583
KLK7	v.5	$F_{(ATG)}$	7R	257
	v.6			246
KLK8	v.7	$F_{(ATG)}$	6R*	330
	v.8			262
	v.9			287
	v.10			271
	v.11			274
	v.12			433
	v.13			593
	v.14			728
	v.15			375
KLK9	v.2	$F_{(ATG)}$	5R*	155
	v.3			421

	v.4			312
	v.5			558
	v.6			539
	v.7			676
	v.8			676
	v.9			813
	v.10			696
	v.11			833

Supplementary Table 4. Primer pairs used in nested RT-PCR for expression analysis of each novel splice variant in a series of established cell lines, originating from several cancerous and/or normal human tissues. These variant-specific pairs of primers were used in nested RT-PCR to amplify each specific splice variant.

Gene	Splice variant	Primer name		Amplicon size (bp)
		Forward	Reverse	
KLK5	v.4	2/6F	6R	108
	v.5	N/3F	5/6R	679
	v.6	N/3F	5/6extR	679
KLK6	v.6	3/4F	4/7R	182
	v.7	3/4F	5/7R	429
	v.8	3/5F	5/7R	268
	v.9	4/6F	6/7R	163
	v.10	N/5F	6/7R	413
	v.11	N/5F	5/7R	276
KLK7	v.5	3/4F	4/7R	171
	v.6	3/6F	6/7R	163
KLK8	v.7	2/3altF	3/6R	183
	v.8	2/N_2F	N_2/6R	115
	v.9	2/N_1F	N_1/6R	140
	v.10	2/6extF	6R	164
	v.11	2/5trF	5/6R	130
	v.12	2/4F	4/6R	287
	v.13	2/3altF	4/6R	446
	v.14	2/3F	4/6R	579
	v.15	2/5trF	5/6extR	131
KLK9	v.2	1/5F	5R	47
	v.3	1/2F	2/5R	178
	v.4	1/3F	3/5R	287

	v.5	1/3F	4/5R	422
	v.6	1/NF	3/5R	404
	v.7	1/NF	4/5R	539
	v.8	2/NtrF	3/5R	380
	v.9	2/NtrF	4/5R	515
	v.10	2/NF	3/5R	407
	v.11	2/NF	4/5R	542

Supplementary Figures

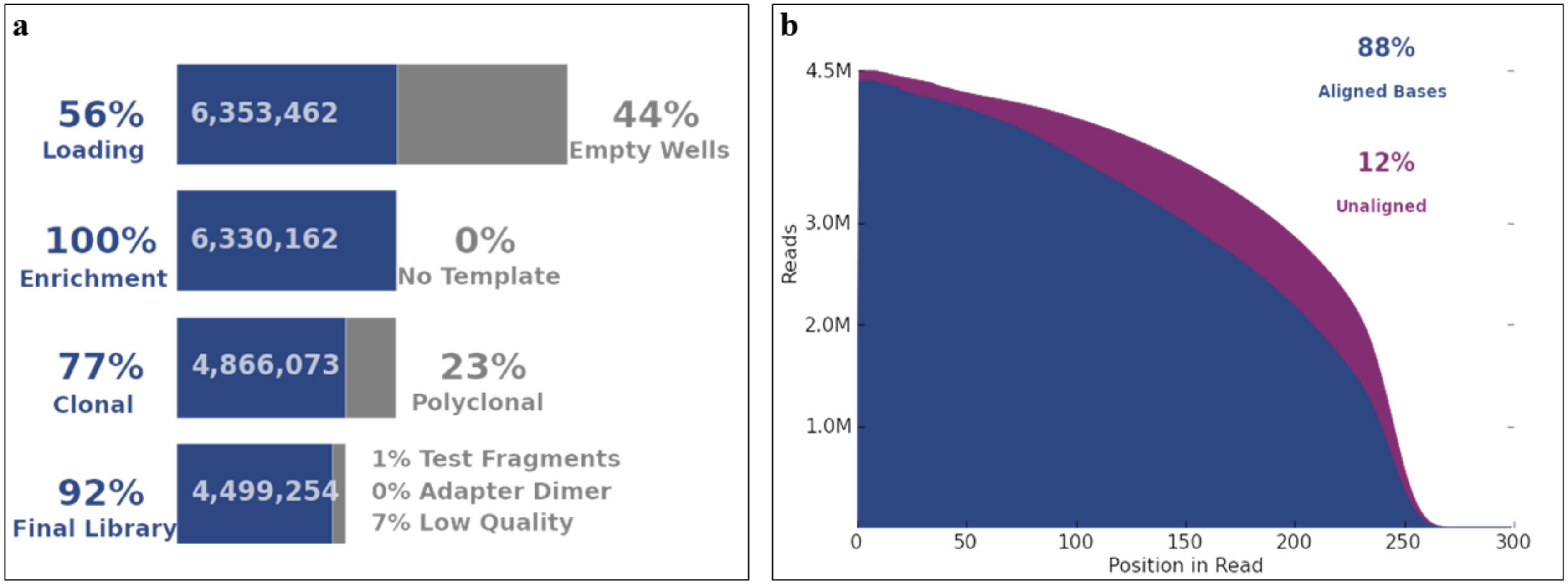

c

Alignment Quality	AQ17	AQ20	Perfect
Total Number of Bases [Mbp]	591 M	520 M	358 M
Mean Length [bp]	174	160	117
Longest Alignment [bp]	305	305	301
Mean Coverage Depth	0.2	0.2	0.1

Supplementary Figure 1. Metrics of the NGS run, carried out in an Ion PGM™ System. (a) Basic metrics of the run, including the percentages of the loaded wells on the Ion 318™ Chip, the enriched template, the clonal ISPs, and the final library that can be used. **(b)** Percentage of bases aligned to the reference sequence. **(c)** Alignment quality of the NGS run.

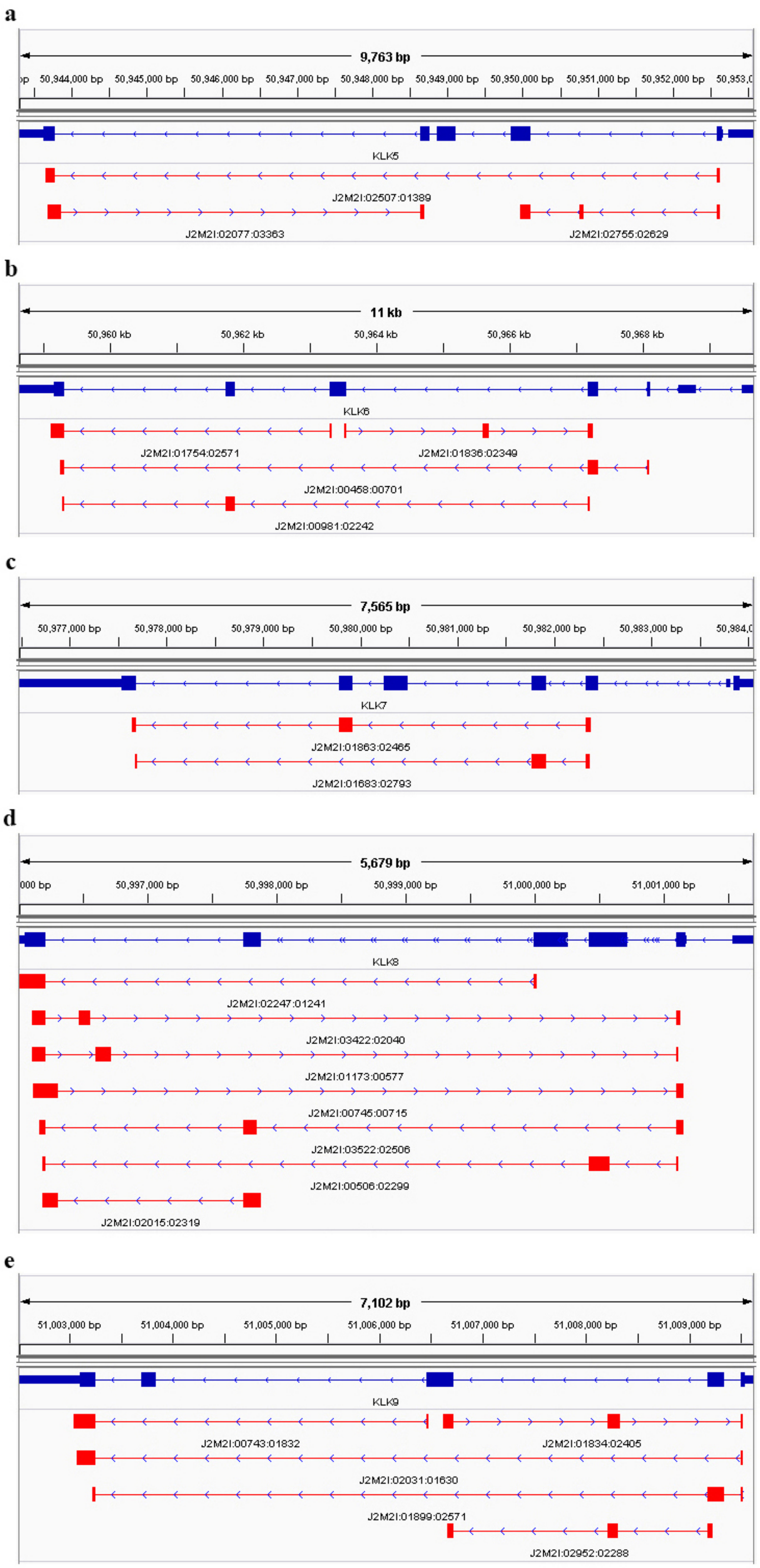

Supplementary Figure 2. Visualization of the generated BED file for each targeted gene using Integrative Genomics Viewer (IGV). The alignment of selected sequencing reads (the same as for Fig. 1) to the reference genomic sequence of **(a)** *KLK5*, **(b)** *KLK6*, **(c)** *KLK7*, **(d)** *KLK8*, and **(e)** *KLK9*, as well as the respective splice junctions are shown.

<u>cDNA FASTA sequences of all novel splice variants</u>

FASTA sequences of cDNA of all novel splice variants of *KLK5*, *KLK6*, *KLK7*, *KLK8*, and *KLK9*. Regions where the primers used in this study bind are highlighted. Light gray sequence at the end of each variant sequence denotes the complementary sequence of the oligo-dT–adapter used in reverse transcription; dark gray sequence at the beginning of each variant sequence marks the rest of the initial exon of each splice variant appearing in Fig. 3 – Fig. 7; all these grey sequences are not included in the submitted sequences of the thirty novel splice variants in GenBank®.

KLK5

```
>KLK5 v.4
GTGCAGCGGCCATGGCTACAGCAAGACCCCCCTGGATGTGGGTGCTCTGTGCTCTGATCACAGCCTTGCT
TCTGGGGGTCACAGGGTGATTCTGGGGGGCCTGTGGTCTGCAATGGCTCCCTGCAGGGACTCGTGTCCTG
GGGAGATTACCCTTGTGCCCGGCCCAACAGACCGGGTGTCTACACGAACCTCTGCAAGTTCACCAAGTGG
ATCCAGGAAACCATCCAGGCCAACTCCTGAGTCATCCCAGGACTCAGCACACCGGCATCCCCACCTGCTG
CAGGGACAGCCCTGACACTCCTTTCAGACCCTCATTCCTTCCCAGAGATGTTGAGAATGTTCATCTCTCC
AGCCCCTGACCCCATGTCTCCTGGACTCAGGGTCTGCTTCCCCCACATTGGGCTGACCGTGTCTCTCTAG
TTGAACCCTGGGAACAATTTCCAAAACTGTCCAGGGCGGGGGTTGCGTCTCAATCTCCCTGGGGCACTTT
CATCCTCAAGCTCAGGGCCCATCCCTTCTCTGCAGCTCTGACCCAAATTTAGTCCCAGAAATAAACTGAG
AAGTGGAAAAAAAAAAAACCTATAGTGAGTCGTATTAATTCTGTGCTCGC
```

Commented [CK1]: *KLK5* F(ATG)
Commented [CK2]: *KLK5* F(Nested)
Commented [CK3]: *KLK5* 2/6F
Commented [CK4]: Reverse complement of *KLK5* 6R
Commented [CK5]: Reverse complement of *KLK5* 6R*
Commented [CK6]: Reverse complement of R(Inner)
Commented [CK7]: Reverse complement of R(Outer)

```
>KLK5 v.5
GTGCAGCGGCCATGGCTACAGCAAGACCCCCCTGGATGTGGGTGCTCTGTGCTCTGATCACAGCCTTGCT
TCTGGGGGTCACAGGTTCAAGTGATTCTCGTGCCTCCACCTCCTGAGTAGCTGGGATTACAGAGCATGTT
CTCGCCAACAATGATGTTTCCTGTGACCACCCCTCTAACACCGTGCCCTCTGGGAGCAACCAGGACCTGG
GAGCTGGGGCCGGGGAAGACGCCCGGTCGGATGACAGCAGCAGCCGCATCATCAATGGATCCGACTGCGA
TATGCACACCCAGCCGTGGCAGGCCGCGCTGTTGCTAAGGCCCAACCAGCTCTACTGCGGGGCGGTGTTG
GTGCATCCACAGTGGCTGCTCACGGCCGCCCACTGCAGGAAGAAAGTTTTCAGAGTCCGTCTCGGCCACT
ACTCCCTGTCACCAGTTTATGAATCTGGGCAGCAGATGTTCCAGGGGGTCAAATCCATCCCCCACCCTGG
CTACTCCCACCCTGGCCACTCTAACGACCTCATGCTCATCAAACTGAACAGAAGAATTCGTCCCACTAAA
GATGTCAGACCCATCAACGTCTCCTCTCATTGTCCCTCTGCTGGGACAAAGTGCTTGGTGTCTGGCTGGG
GGACAACCAAGAGCCCCCAAGTGCACTTCCCTAAGGTCCTCCAGTGCTTGAATATCAGCGTGCTAAGTCA
GAAAAGGTGCGAGGATGCTTACCCGAGACAGATAGATGACACCATGTTCTGCGCCGGTGACAAAGCAGGT
AGAGACTCCTGCCAGGGTGATTCTGGGGGGCCTGTGGTCTGCAATGGCTCCCTGCAGGGACTCGTGTCCT
GGGGAGATTACCCTTGTGCCCGGCCCAACAGACCGGGTGTCTACACGAACCTCTGCAAGTTCACCAAGTG
GATCCAGGAAACCATCCAGGCCAACTCCTGAGTCATCCCAGGACTCAGCACACCGGCATCCCCACCTGCT
GCAGGGACAGCCCTGACACTCCTTTCAGACCCTCATTCCTTCCCAGAGATGTTGAGAATGTTCATCTCTC
CAGCCCCTGACCCCATGTCTCCTGGACTCAGGGTCTGCTTCCCCCACATTGGGCTGACCGTGTCTCTCTA
GTTGAACCCTGGGAACAATTTCCAAAACTGTCCAGGGCGGGGGTTGCGTCTCAATCTCCCTGGGGCACTT
TCATCCTCAAGCTCAGGGCCCATCCCTTCTCTGCAGCTCTGACCCAAATTTAGTCCCAGAAATAAACTGA
GAAGTGGAAAAAAAAAAAACCTATAGTGAGTCGTATTAATTCTGTGCTCGC
```

Commented [CK8]: *KLK5* F(ATG)
Commented [CK9]: *KLK5* F(Nested)
Commented [CK10]: *KLK5* N/3F
Commented [CK11]: Reverse complement of *KLK5* 5/6R
Commented [CK12]: Reverse complement of *KLK5* 6R*
Commented [CK13]: Reverse complement of R(Inner)
Commented [CK14]: Reverse complement of R(Outer)

```
>KLK5 v.6
GTGCAGCGGCCATGGCTACAGCAAGACCCCCCTGGATGTGGGTGCTCTGTGCTCTGATCACAGCCTTGCT
TCTGGGGGTCACAGGTTCAAGTGATTCTCGTGCCTCCACCTCCTGAGTAGCTGGGATTACAGAGCATGTT
CTCGCCAACAATGATGTTTCCTGTGACCACCCCTCTAACACCGTGCCCTCTGGGAGCAACCAGGACCTGG
GAGCTGGGGCCGGGGAAGACGCCCGGTCGGATGACAGCAGCAGCCGCATCATCAATGGATCCGACTGCGA
TATGCACACCCAGCCGTGGCAGGCCGCGCTGTTGCTAAGGCCCAACCAGCTCTACTGCGGGGCGGTGTTG
GTGCATCCACAGTGGCTGCTCACGGCCGCCCACTGCAGGAAGAAAGTTTTCAGAGTCCGTCTCGGCCACT
ACTCCCTGTCACCAGTTTATGAATCTGGGCAGCAGATGTTCCAGGGGGTCAAATCCATCCCCCACCCTGG
CTACTCCCACCCTGGCCACTCTAACGACCTCATGCTCATCAAACTGAACAGAAGAATTCGTCCCACTAAA
GATGTCAGACCCATCAACGTCTCCTCTCATTGTCCCTCTGCTGGGACAAAGTGCTTGGTGTCTGGCTGGG
GGACAACCAAGAGCCCCCAAGTGCACTTCCCTAAGGTCCTCCAGTGCTTGAATATCAGCGTGCTAAGTCA
GAAAAGGTGCGAGGATGCTTACCCGAGACAGATAGATGACACCATGTTCTGCGCCGGTGACAAAGCAGGT
AGAGACTCCTGCCAGGCATCTCTGGGTCTCTCATGTCTCCTTCTGCCCACTTTGCCACATCTCTGCCTCT
CTCATGCCCCCCTTTCTCTCCTGCAGGGTGATTCTGGGGGGCCTGTGGTCTGCAATGGCTCCCTGCAGGG
ACTCGTGTCCTGGGGAGATTACCCTTGTGCCCGGCCCAACAGACCGGGTGTCTACACGAACCTCTGCAAG
TTCACCAAGTGGATCCAGGAAACCATCCAGGCCAACTCCTGAGTCATCCCAGGACTCAGCACACCGGCAT
CCCCACCTGCTGCAGGGACAGCCCTGACACTCCTTTCAGACCCTCATTCCTTCCCAGAGATGTTGAGAAT
GTTCATCTCTCCAGCCCCTGACCCCATGTCTCCTGGACTCAGGGTCTGCTTCCCCCACATTGGGCTGACC
GTGTCTCTCTAGTTGAACCCTGGGAACAATTTCCAAAACTGTCCAGGGCGGGGGTTGCGTCTCAATCTCC
CTGGGGCACTTTCATCCTCAAGCTCAGGGCCCATCCCTTCTCTGCAGCTCTGACCCAAATTTAGTCCCAG
AAATAAACTGAGAAGTGGAAAAAAAAAAAACCTATAGTGAGTCGTATTAATTCTGTGCTCGC
```

Commented [CK15]: *KLK5* F(ATG)
Commented [CK16]: *KLK5* F(Nested)
Commented [CK17]: *KLK5* N/3F
Commented [CK18]: Reverse complement of *KLK5* 5/6extR
Commented [CK19]: Reverse complement of *KLK5* 6R*
Commented [CK20]: Reverse complement of R(Inner)
Commented [CK21]: Reverse complement of R(Outer)

KLK6

```
>KLK6 v.6
GAGCGGCCATGAAGAAGCTGATGGTGGTGCTGAGTCTGATTGCTGCAGCCTGGGCAGAGGAGCAGAATAA
GTTGGTGCATGGCGGACCCTGCGACAAGACATCTCACCCCTACCAAGCTGCCCTCTACACCTCGGGCCAC
TTGCTCTGTGGTGGGGTCCTTATCCATCCACTGTGGGTCCTCACAGCTGCCCACTGCAAAAAACCGGTGA
TTCTGGGGGTCCGCTGGTATGTGGAGACCACCTCCGAGGCCTTGTGTCATGGGGTAACATCCCCTGTGGA
TCAAAGGAGAAGCCAGGAGTCTACACCAACGTCTGCAGATACACGAACTGGATCCAAAAAACCATTCAGG
CCAAGTGACCCTGACATGTGACATCTACCTCCCGACCTACCACCCCACTGGCTGGTTCCAGAACGTCTCT
CACCTAGACCTTGCCTCCCCTCCTCTCCTGCCCAGCTCTGACCCTGATGCTTAATAAACGCAGCGACGTG
AGGGTCCTGATTCTCCCTGGTTTTACCCCAGCTCCATCCTTGCATCACTGGGGAGGACGTGATGAGTGAG
GACTTGGGTCCTCGGTCTTACCCCCACCACTAAGAGAATACAGGAAAATCCCTTCTAGGCATCTCCTCTC
CCCAACCCTTCCACACGTTTGATTTCTTCCTGCAGAGGCCCAGCCACGTGTCTGGAATCCCAGCTCCGCT
GCTTACTGTCGGTGTCCCCTTGGGATGTACCTTTCTTCACTGCAGATTTCTCACCTGTAAGATGAAGATA
AGGATGATACAGTCTCCATAAGGCAGTGGCTGTTGGAAAGATTTAAGGTTTCACACCTATGACATACATG
GAATAGCACCTGGGCCACCATGCACTCAATAAAGAATGAATTTTATTATGAAAAAAAAAAAACCTATAGT
GAGTCGTATTAATTCTGTGCTCGC
```

```
>KLK6 v.7
GAGCGGCCATGAAGAAGCTGATGGTGGTGCTGAGTCTGATTGCTGCAGCCTGGGCAGAGGAGCAGAATAA
GTTGGTGCATGGCGGACCCTGCGACAAGACATCTCACCCCTACCAAGCTGCCCTCTACACCTCGGGCCAC
TTGCTCTGTGGTGGGGTCCTTATCCATCCACTGTGGGTCCTCACAGCTGCCCACTGCAAAAAACCGAATC
TTCAGGTCTTCCTGGGGAAGCATAACCTTCGGCAAAGGGAGAGTTCCCAGGAGCAGAGTTCTGTTGTCCG
GGCTGTGATCCACCCTGACTATGATGCCGCCAGCCATGACCAGGACATCATGCTGTTGCGCCTGGCACGC
CCAGCCAAACTCTCTGAACTCATCCAGCCCCTTCCCCTGGAGAGGGACTGCTCAGCCAACACCACCAGCT
GCCACATCCTGGGCTGGGGCAAGACAGCAGATGGGTGATTCTGGGGGTCCGCTGGTATGTGGAGACCACC
TCCGAGGCCTTGTGTCATGGGGTAACATCCCCTGTGGATCAAAGGAGAAGCCAGGAGTCTACACCAACGT
CTGCAGATACACGAACTGGATCCAAAAAACCATTCAGGCCAAGTGACCCTGACATGTGACATCTACCTCC
CGACCTACCACCCCACTGGCTGGTTCCAGAACGTCTCTCACCTAGACCTTGCCTCCCCTCCTCTCCTGCC
CAGCTCTGACCCTGATGCTTAATAAACGCAGCGACGTGAGGGTCCTGATTCTCCCTGGTTTTACCCCAGC
TCCATCCTTGCATCACTGGGGAGGACGTGATGAGTGAGGACTTGGGTCCTCGGTCTTACCCCCACCACTA
AGAGAATACAGGAAAATCCCTTCTAGGCATCTCCTCTCCCCAACCCTTCCACACGTTTGATTTCTTCCTG
CAGAGGCCCAGCCACGTGTCTGGAATCCCAGCTCCGCTGCTTACTGTCGGTGTCCCCTTGGGATGTACCT
TTCTTCACTGCAGATTTCTCACCTGTAAGATGAAGATAAGGATGATACAGTCTCCATAAGGCAGTGGCTG
TTGGAAAGATTTAAGGTTTCACACCTATGACATACATGGAATAGCACCTGGGCCACCATGCACTCAATAA
AGAATGAATTTTATTATGAAAAAAAAAAAACCTATAGTGAGTCGTATTAATTCTGTGCTCGC
```

```
>KLK6 v.8
GAGCGGCCATGAAGAAGCTGATGGTGGTGCTGAGTCTGATTGCTGCAGGAATCTTCAGGTCTTCCTGGGG
AAGCATAACCTTCGGCAAAGGGAGAGTTCCCAGGAGCAGAGTTCTGTTGTCCGGGCTGTGATCCACCCTG
ACTATGATGCCGCCAGCCATGACCAGGACATCATGCTGTTGCGCCTGGCACGCCCAGCCAAACTCTCTGA
ACTCATCCAGCCCCTTCCCCTGGAGAGGGACTGCTCAGCCAACACCACCAGCTGCCACATCCTGGGCTGG
GGCAAGACAGCAGATGGGTGATTCTGGGGGTCCGCTGGTATGTGGAGACCACCTCCGAGGCCTTGTGTCA
TGGGGTAACATCCCCTGTGGATCAAAGGAGAAGCCAGGAGTCTACACCAACGTCTGCAGATACACGAACT
GGATCCAAAAAACCATTCAGGCCAAGTGACCCTGACATGTGACATCTACCTCCCGACCTACCACCCCACT
GGCTGGTTCCAGAACGTCTCTCACCTAGACCTTGCCTCCCCTCCTCTCCTGCCCAGCTCTGACCCTGATG
CTTAATAAACGCAGCGACGTGAGGGTCCTGATTCTCCCTGGTTTTACCCCAGCTCCATCCTTGCATCACT
GGGGAGGACGTGATGAGTGAGGACTTGGGTCCTCGGTCTTACCCCCACCACTAAGAGAATACAGGAAAAT
CCCTTCTAGGCATCTCCTCTCCCCAACCCTTCCACACGTTTGATTTCTTCCTGCAGAGGCCCAGCCACGT
GTCTGGAATCCCAGCTCCGCTGCTTACTGTCGGTGTCCCCTTGGGATGTACCTTTCTTCACTGCAGATTT
CTCACCTGTAAGATGAAGATAAGGATGATACAGTCTCCATAAGGCAGTGGCTGTTGGAAAGATTTAAGGT
TTCACACCTATGACATACATGGAATAGCACCTGGGCCACCATGCACTCAATAAAGAATGAATTTTATTAT
GAAAAAAAAAAAAACCTATAGTGAGTCGTATTAATTCTGTGCTCGC
```

```
>KLK6 v.9
GAGCGGCCATGAAGAAGCTGATGGTGGTGCTGAGTCTGATTGCTGCAGCCTGGGCAGAGGAGCAGAATAA
GTTGGTGCATGGCGGACCCTGCGACAAGACATCTCACCCCTACCAAGCTGCCCTCTACACCTCGGGCCAC
TTGCTCTGTGGTGGGGTCCTTATCCATCCACTGTGGGTCCTCACAGCTGCCCACTGCAAAAAACCGTGAT
```

Commented [CK22]: *KLK6* $F_{(ATG)}$

Commented [CK23]: *KLK6* $F_{(Nested)}$

Commented [CK24]: *KLK6* 3/4F

Commented [CK25]: Reverse complement of *KLK6* 4/7R

Commented [CK26]: Reverse complement of *KLK6* 7R

Commented [CK27]: Reverse complement of $R_{(Inner)}$

Commented [CK28]: Reverse complement of $R_{(Outer)}$

Commented [CK29]: Reverse complement of $R_{(Outer)}$

Commented [CK30]: *KLK6* $F_{(ATG)}$

Commented [CK31]: *KLK6* $F_{(Nested)}$

Commented [CK32]: *KLK6* 3/4F

Commented [CK33]: Reverse complement of *KLK6* 5/7R

Commented [CK34]: Reverse complement of *KLK6* 7R

Commented [CK35]: Reverse complement of $R_{(Inner)}$

Commented [CK36]: Reverse complement of $R_{(Outer)}$

Commented [CK37]: *KLK6* $F_{(ATG)}$

Commented [CK38]: *KLK6* $F_{(Nested)}$

Commented [CK39]: *KLK6* 3/5F

Commented [CK40]: Reverse complement of *KLK6* 5/7R

Commented [CK41]: Reverse complement of *KLK6* 7R

Commented [CK42]: Reverse complement of $R_{(Inner)}$

Commented [CK43]: Reverse complement of $R_{(Outer)}$

Commented [CK44]: *KLK6* $F_{(ATG)}$

Commented [CK45]: *KLK6* $F_{(Nested)}$

TTCCCTGACACCATCCAGTGTGCATACATCCACCTGGTGTCCCGTGAGGAGTGTGAGCATGCCTACCCTG
GCCAGATCACCCAGAACATGTTGTGTGCTGGGGATGAGAAGTACGGGAAGGATTCCTGCCAGGGTGATTC
TGGGGGTCCGCTGGTATGTGGAGACCACCTCCGAGGCCTTGTGTCATGGGGTAACATCCCCTGTGGATCA
AAGGAGAAGCCAGGAGTCTACACCAACGTCTGCAGATACACGAACTGGATCCAAAAAACCATTCAGGCCA
AGTGACCCTGACATGTGACATCTACCTCCCGACCTACCACCCCACTGGCTGGTTCCAGAACGTCTCTCAC
CTAGACCTTGCCTCCCCTCCTCTCCTGCCCAGCTCTGACCCTGATGCTTAATAAACGCAGCGACGTGAGG
GTCCTGATTCTCCCTGGTTTTACCCCAGCTCCATCCTTGCATCACTGGGGAGGACGTGATGAGTGAGGAC
TTGGGTCCTCGGTCTTACCCCCACCACTAAGAGAATACAGGAAAATCCCTTCTAGGCATCTCCTCTCCCC
AACCCTTCCACACGTTTGATTTCTTCCTGCAGAGGCCCAGCCACGTGTCTGGAATCCCAGCTCCGCTGCT
TACTGTCGGTGTCCCCTTGGGATGTACCTTTCTTCACTGCAGATTTCTCACCTGTAAGATGAAGATAAGG
ATGATACAGTCTCCATAAGGCAGTGGCTGTTGGAAAGATTTAAGGTTTCACACCTATGACATACATGGAA
TAGCACCTGGGCCACCATGCACTCAATAAAGAATGAATTTTATTATGAAAAAAAAAAAACCTATAGTGAG
TCGTATTAATTCTGTGCTCGC

>KLK6 v.10
GAGCGGCCATGAAGAAGCTGATGGTGGTGCTGAGTCTGATTGCTGCAGCCTGGGCAGAGGAGCAGAATAA
GTTGGTGCATGGCGGACCCTGCGACAAGACATCTCACCCCTACCAAGCTGCCCTCTACACCTCGGGCCAC
TTGCTCTGTGGTGGGGTCCTTATCCATCCACTGTGGGTCCTCACAGCTGCCCACTGCAAAAAACCAGCAC
CTGCTAAGTGCCACATTCGAGTTGGGCAGTGGAGATTCAGCAATGGATGGGACACACACGTCATCCCTGC
CCTCGGGAGCACAAGGACAGAAAGGAATCTTCAGGTCTTCCTGGGGAAGCATAACCTTCGGCAAAGGGAG
AGTTCCCAGGAGCAGAGTTCTGTTGTCCGGGCTGTGATCCACCCTGACTATGATGCCGCCAGCCATGACC
AGGACATCATGCTGTTGCGCCTGGCACGCCCAGCCAAACTCTCTGAACTCATCCAGCCCCTTCCCCTGGA
GAGGGACTGCTCAGCCAACACCACCAGCTGCCACATCCTGGGCTGGGGCAAGACAGCAGATGGTGATTTC
CCTGACACCATCCAGTGTGCATACATCCACCTGGTGTCCCGTGAGGAGTGTGAGCATGCCTACCCTGGCC
AGATCACCCAGAACATGTTGTGTGCTGGGGATGAGAAGTACGGGAAGGATTCCTGCCAGGGTGATTCTGG
GGGTCCGCTGGTATGTGGAGACCACCTCCGAGGCCTTGTGTCATGGGGTAACATCCCCTGTGGATCAAAG
GAGAAGCCAGGAGTCTACACCAACGTCTGCAGATACACGAACTGGATCCAAAAAACCATTCAGGCCAAGT
GACCCTGACATGTGACATCTACCTCCCGACCTACCACCCCACTGGCTGGTTCCAGAACGTCTCTCACCTA
GACCTTGCCTCCCCTCCTCTCCTGCCCAGCTCTGACCCTGATGCTTAATAAACGCAGCGACGTGAGGGTC
CTGATTCTCCCTGGTTTTACCCCAGCTCCATCCTTGCATCACTGGGGAGGACGTGATGAGTGAGGACTTG
GGTCCTCGGTCTTACCCCCACCACTAAGAGAATACAGGAAAATCCCTTCTAGGCATCTCCTCTCCCCAAC
CCTTCCACACGTTTGATTTCTTCCTGCAGAGGCCCAGCCACGTGTCTGGAATCCCAGCTCCGCTGCTTAC
TGTCGGTGTCCCCTTGGGATGTACCTTTCTTCACTGCAGATTTCTCACCTGTAAGATGAAGATAAGGATG
ATACAGTCTCCATAAGGCAGTGGCTGTTGGAAAGATTTAAGGTTTCACACCTATGACATACATGGAATAG
CACCTGGGCCACCATGCACTCAATAAAGAATGAATTTTATTATGAAAAAAAAAAAACCTATAGTGAGTCG
TATTAATTCTGTGCTCGC

>KLK6 v.11
GAGCGGCCATGAAGAAGCTGATGGTGGTGCTGAGTCTGATTGCTGCAGCCTGGGCAGAGGAGCAGAATAA
GTTGGTGCATGGCGGACCCTGCGACAAGACATCTCACCCCTACCAAGCTGCCCTCTACACCTCGGGCCAC
TTGCTCTGTGGTGGGGTCCTTATCCATCCACTGTGGGTCCTCACAGCTGCCCACTGCAAAAAACCAGCAC
CTGCTAAGTGCCACATTCGAGTTGGGCAGTGGAGATTCAGCAATGGATGGGACACACACGTCATCCCTGC
CCTCGGGAGCACAAGGACAGAAAGGAATCTTCAGGTCTTCCTGGGGAAGCATAACCTTCGGCAAAGGGAG
AGTTCCCAGGAGCAGAGTTCTGTTGTCCGGGCTGTGATCCACCCTGACTATGATGCCGCCAGCCATGACC
AGGACATCATGCTGTTGCGCCTGGCACGCCCAGCCAAACTCTCTGAACTCATCCAGCCCCTTCCCCTGGA
GAGGGACTGCTCAGCCAACACCACCAGCTGCCACATCCTGGGCTGGGGCAAGACAGCAGATGGGTGATTC
TGGGGGTCCGCTGGTATGTGGAGACCACCTCCGAGGCCTTGTGTCATGGGGTAACATCCCCTGTGGATCA
AAGGAGAAGCCAGGAGTCTACACCAACGTCTGCAGATACACGAACTGGATCCAAAAAACCATTCAGGCCA
AGTGACCCTGACATGTGACATCTACCTCCCGACCTACCACCCCACTGGCTGGTTCCAGAACGTCTCTCAC
CTAGACCTTGCCTCCCCTCCTCTCCTGCCCAGCTCTGACCCTGATGCTTAATAAACGCAGCGACGTGAGG
GTCCTGATTCTCCCTGGTTTTACCCCAGCTCCATCCTTGCATCACTGGGGAGGACGTGATGAGTGAGGAC
TTGGGTCCTCGGTCTTACCCCCACCACTAAGAGAATACAGGAAAATCCCTTCTAGGCATCTCCTCTCCCC
AACCCTTCCACACGTTTGATTTCTTCCTGCAGAGGCCCAGCCACGTGTCTGGAATCCCAGCTCCGCTGCT
TACTGTCGGTGTCCCCTTGGGATGTACCTTTCTTCACTGCAGATTTCTCACCTGTAAGATGAAGATAAGG
ATGATACAGTCTCCATAAGGCAGTGGCTGTTGGAAAGATTTAAGGTTTCACACCTATGACATACATGGAA
TAGCACCTGGGCCACCATGCACTCAATAAAGAATGAATTTTATTATGAAAAAAAAAAAACCTATAGTGAG
TCGTATTAATTCTGTGCTCGC

Commented [CK46]: *KLK6* 4/6F

Commented [CK47]: Reverse complement of *KLK6* 6/7R

Commented [CK48]: Reverse complement of *KLK6* 7R

Commented [CK49]: Reverse complement of $R_{(Inner)}$

Commented [CK50]: Reverse complement of $R_{(Outer)}$

Commented [CK51]: *KLK6* $F_{(ATG)}$

Commented [CK52]: *KLK6* $F_{(Nested)}$

Commented [CK53]: *KLK6* N/5F

Commented [CK54]: Reverse complement of *KLK6* 6/7R

Commented [CK55]: Reverse complement of *KLK6* 7R

Commented [CK56]: Reverse complement of $R_{(Inner)}$

Commented [CK57]: Reverse complement of $R_{(Outer)}$

Commented [CK58]: Reverse complement of $R_{(Outer)}$

Commented [CK59]: *KLK6* $F_{(ATG)}$

Commented [CK60]: *KLK6* $F_{(Nested)}$

Commented [CK61]: *KLK6* N/5F

Commented [CK62]: Reverse complement of *KLK6* 5/7R

Commented [CK63]: Reverse complement of *KLK6* 7R

Commented [CK64]: Reverse complement of $R_{(Inner)}$

Commented [CK65]: Reverse complement of $R_{(Outer)}$

Commented [CK66]: Reverse complement of $R_{(Outer)}$

KLK7

>KLK7 v.5
CTCCAGCAGGAGAGGCCCTTCCTCGCCTGGCAGCCCCTGAGCGGCTCAGCAGGGCACCATGGCAAGATCC
CTTCTCCTGCCCCTGCAGATCTTACTGCTATCCTTAGCCTTGGAAACTGCAGGAGAAGAAGCCCAGGGTG
ACAAGATTATTGATGGCGCCCCATGTGCAAGAGGCTCCCACCCATGGCAGGTGGCCCTGCTCAGTGGCAA
TCAGCTCCACTGCGGAGGCGTCCTGGTCAATGAGCGCTGGGTGCTCACTGCCGCCCACTGCAAGATGAAG
GTGACTCAGGGGGACCGTTGGTGTGCAGAGGTACCCTGCAAGGTCTGGTGTCCTGGGGAACTTTCCCTTG
CGGCCAACCCAATGACCCAGGAGTCTACACTCAAGTGTGCAAGTTCACCAAGTGGATAAATGACACCATG
AAAAAGCATCGCTAACGCCACACTGAGTTAATTAACTGTGTGCTTCCAACAGAAAATGCACAGGAGTGAG
GACGCCGATGACCTATGAAGTCAAATTTGACTTTACCTTTCCTCAAAGATATATTTAAACCAACCTCATG
CCCTGTTGATAAACCAATCAAATTGGTAAAGACCTAAAACCAAAACAAATAAAGAAACACAAAACCCTCA
GTGCTGGAGAAGAGTCAGTGAGACCAGCACTCTCAAACACTGGAACTGGACGTTCGTACAGTCTTTACGG
AAGACACTTGGTCAACGTACACCGAGACCCTTATTCACCACCTTTGACCCAGTAACTCTAATCTTAGGAA
GAACCTACTGAAACAAAAAAAATCCAAAATGTAGAACAAGACTTGAATTTACCATGATATTATTTATCAC
AGAAATGAAGTGAAACCATCAAACATGTTCCAAAAGTACCAGATGGCTTAAATAATAGTCTGGCTTGGCA
CAACGATGTTTTTTTTCTTTGAGACAGAGTCTCTGTTGCTTGGGCTGCAATGCAGTGATGCAATCTTGGC
TCACTGCAACCTCCGCCTCCTGGGTTCAAGTGATTCTCGTGCTTCAGCCTCCCAAGTACCTGGGACTACA
GGTGTGCACCACCACACCAGGCTAATTTTTTGTGTATTTTTACTAGAGACAGGGTTTCACCATGTTGGCC
AGCATGGTCTTGAACGCCTGACCTCAGATGATCCACCCACCTTGGCCTCCCAAAGTGCTGGGATTACAGG
CATGAGCCACCACGGCCAGCCCACAATGATATTACAAACCTATTAAAAATGATACTTAGACAGAATTGTC
AGTATTATTCAAGAACATTTAGGCTATAGGATGTTAAATGACAAAAGGAAGGACAAAAATATATATGTAT
GTGACCCTACCCATAAAAAATGAAATATTCACAGAATCAGATCTGAAAACACATGTCCCAGACTGCATAC
TGGGGTCGTCATGAGGTGTCTCCTTCCTTCTGTGTACTTTTCCTTGAATGTGCACTTTTATAACATGAAA
AATAAAGGTGGGGAAAAAAGTCAAAAAAAAAAAACCTATAGTGAGTCGTATTAATTCTGTGCTCGC

>KLK7 v.6
CTCCAGCAGGAGAGGCCCTTCCTCGCCTGGCAGCCCCTGAGCGGCTCAGCAGGGCACCATGGCAAGATCC
CTTCTCCTGCCCCTGCAGATCTTACTGCTATCCTTAGCCTTGGAAACTGCAGGAGAAGAAGTGACCTTTC
CCTCTGACCTCATGTGCGTGGATGTCAAGCTCATCTCCCCCCAGGACTGCACGAAGGTTTACAAGGACTT
ACTGGAAAATTCCATGCTGTGCGCTGGCATCCCCGACTCCAAGAAAAACGCCTGCAATGGTGACTCAGGG
GGACCGTTGGTGTGCAGAGGTACCCTGCAAGGTCTGGTGTCCTGGGGAACTTTCCCTTGCGGCCAACCCA
ATGACCCAGGAGTCTACACTCAAGTGTGCAAGTTCACCAAGTGGATAAATGACACCATGAAAAAGCATCG
CTAACGCCACACTGAGTTAATTAACTGTGTGCTTCCAACAGAAAATGCACAGGAGTGAGGACGCCGATGA
CCTATGAAGTCAAATTTGACTTTACCTTTCCTCAAAGATATATTTAAACCAACCTCATGCCCTGTTGATA
AACCAATCAAATTGGTAAAGACCTAAAACCAAAACAAATAAAGAAACACAAAACCCTCAGTGCTGGAGAA
GAGTCAGTGAGACCAGCACTCTCAAACACTGGAACTGGACGTTCGTACAGTCTTTACGGAAGACACTTGG
TCAACGTACACCGAGACCCTTATTCACCACCTTTGACCCAGTAACTCTAATCTTAGGAAGAACCTACTGA
AACAAAAAAAATCCAAAATGTAGAACAAGACTTGAATTTACCATGATATTATTTATCACAGAAATGAAGT
GAAACCATCAAACATGTTCCAAAAGTACCAGATGGCTTAAATAATAGTCTGGCTTGGCACAACGATGTTT
TTTTTCTTTGAGACAGAGTCTCTGTTGCTTGGGCTGCAATGCAGTGATGCAATCTTGGCTCACTGCAACC
TCCGCCTCCTGGGTTCAAGTGATTCTCGTGCTTCAGCCTCCCAAGTACCTGGGACTACAGGTGTGCACCA
CCACACCAGGCTAATTTTTTGTGTATTTTTACTAGAGACAGGGTTTCACCATGTTGGCCAGCATGGTCTT
GAACGCCTGACCTCAGATGATCCACCCACCTTGGCCTCCCAAAGTGCTGGGATTACAGGCATGAGCCACC
ACGGCCAGCCCACAATGATATTACAAACCTATTAAAAATGATACTTAGACAGAATTGTCAGTATTATTCA
AGAACATTTAGGCTATAGGATGTTAAATGACAAAAGGAAGGACAAAAATATATATGTATGTGACCCTACC
CATAAAAAATGAAATATTCACAGAATCAGATCTGAAAACACATGTCCCAGACTGCATACTGGGGTCGTCA
TGAGGTGTCTCCTTCCTTCTGTGTACTTTTCCTTGAATGTGCACTTTTATAACATGAAAAATAAAGGTGG
GGAAAAAAGTCAAAAAAAAAAAACCTATAGTGAGTCGTATTAATTCTGTGCTCGC

Commented [CK67]: *KLK7* $F_{(ATG)}$

Commented [CK68]: *KLK7* $F_{(Nested)}$

Commented [CK69]: *KLK7* 3/4F

Commented [CK70]: Reverse complement of *KLK7* 4/7R

Commented [CK71]: Reverse complement of *KLK7* 7R

Commented [CK72]: Reverse complement of $R_{(Inner)}$

Commented [CK73]: Reverse complement of $R_{(Outer)}$

Commented [CK74]: *KLK7* $F_{(ATG)}$

Commented [CK75]: *KLK7* $F_{(Nested)}$

Commented [CK76]: *KLK7* 3/6F

Commented [CK77]: Reverse complement of *KLK7* 6/7R

Commented [CK78]: Reverse complement of *KLK7* 7R

Commented [CK79]: Reverse complement of $R_{(Inner)}$

Commented [CK80]: Reverse complement of $R_{(Outer)}$

KLK8

>KLK8 v.7
ACCTCACCATGGGACGCCCCCGACCTCGTGCGGCCAAGACGTGGATGTTCCTGCTCTTGCTGGGGGGAGC
CTGGGCAGGACACTCCAGGGCACAGGAGGACAAGGTGCTGGGGGGTCATGAGTGCCAACCCCATTCGCAG
CCTTGGCAGGCGGCCTTGTTCCAGGGCCAGCAACTACTCTGTGGCGGTGTCCTTGTAGGTGGCAACTGGG
TCCTTACAGCTGCCCACTGTAAAAAACCGGCGATTCTGGAGGCCCCCTGGTGTGTGATGGTGCACTCCAG
GGCATCACATCCTGGGGCTCAGACCCCTGTGGGAGGTCCGACAAACCTGGCGTCTATACCAACATCTGCC
GCTACCTGGACTGGATCAAGAAGATCATAGGCAGCAAGGGCTGATTCTAGGATAAGCACTAGATCTCCCT
TAATAAACTCACAACTCTCTGGTTCAAAAAAAAAAAACCTATAGTGAGTCGTATTAATTCTGTGCTCGC

>KLK8 v.8
ACCTCACCATGGGACGCCCCCGACCTCGTGCGGCCAAGACGTGGATGTTCCTGCTCTTGCTGGGGGGAGC
CTGGGCAGAGACAGGGATTCACCATGTTGGCCAGGCTGGTCTGGAACGCCTGACTTCAAGTGATCTACCG
CCTCGGCCTCCCAAAGTGCCGGGATTGCAGGGCGATTCTGGAGGCCCCCTGGTGTGTGATGGTGCACTCC
AGGGCATCACATCCTGGGGCTCAGACCCCTGTGGGAGGTCCGACAAACCTGGCGTCTATACCAACATCTG
CCGCTACCTGGACTGGATCAAGAAGATCATAGGCAGCAAGGGCTGATTCTAGGATAAGCACTAGATCTCC
CTTAATAAACTCACAACTCTCTGGTTCAAAAAAAAAAAACCTATAGTGAGTCGTATTAATTCTGTGCTCG
C

>KLK8 v.9
ACCTCACCATGGGACGCCCCCGACCTCGTGCGGCCAAGACGTGGATGTTCCTGCTCTTGCTGGGGGGAGC
CTGGGCAGGGTCTCGCTCTGTCCCCCAAGCTGGAATGCAGTGGCACAATCTTGGCTCACTGCAGCCTCTG
CCTCCCAGTTCAAGCAATTCTCCCACCTCAGCCTCCCGAGTAGCTGTGATTACAGGGCGATTCTGGAGGC
CCCCTGGTGTGTGATGGTGCACTCCAGGGCATCACATCCTGGGGCTCAGACCCCTGTGGGAGGTCCGACA
AACCTGGCGTCTATACCAACATCTGCCGCTACCTGGACTGGATCAAGAAGATCATAGGCAGCAAGGGCTG
ATTCTAGGATAAGCACTAGATCTCCCTTAATAAACTCACAACTCTCTGGTTCAAAAAAAAAAAACCTATA
GTGAGTCGTATTAATTCTGTGCTCGC

>KLK8 v.10
ACCTCACCATGGGACGCCCCCGACCTCGTGCGGCCAAGACGTGGATGTTCCTGCTCTTGCTGGGGGGAGC
CTGGGCAGGGGACCAATCATGCCAAAGAACTGGTAAAACGCTGGGACAGCAGGAAAAGGGACGTTGTGGA
CATCTCAGATGCAAGGCTGTTCTCATTCTCCCTGTCTAGGGCGATTCTGGAGGCCCCCTGGTGTGTGATG
GTGCACTCCAGGGCATCACATCCTGGGGCTCAGACCCCTGTGGGAGGTCCGACAAACCTGGCGTCTATAC
CAACATCTGCCGCTACCTGGACTGGATCAAGAAGATCATAGGCAGCAAGGGCTGATTCTAGGATAAGCAC
TAGATCTCCCTTAATAAACTCACAACTCTCTGGTTCAAAAAAAAAAAACCTATAGTGAGTCGTATTAATT
CTGTGCTCGC

>KLK8 v.11
ACCTCACCATGGGACGCCCCCGACCTCGTGCGGCCAAGACGTGGATGTTCCTGCTCTTGCTGGGGGGAGC
CTGGGCAGAAGTAAAAATCTTTCCCCAGAAGAAGTGTGAGGATGCTTACCCGGGGCAGATCACAGATGGC
ATGGTCTGTGCAGGCAGCAGCAAAGGGGCTGACACGTGCCAGGGCGATTCTGGAGGCCCCCTGGTGTGTG
ATGGTGCACTCCAGGGCATCACATCCTGGGGCTCAGACCCCTGTGGGAGGTCCGACAAACCTGGCGTCTA
TACCAACATCTGCCGCTACCTGGACTGGATCAAGAAGATCATAGGCAGCAAGGGCTGATTCTAGGATAAG
CACTAGATCTCCCTTAATAAACTCACAACTCTCTGGTTCAAAAAAAAAAAACCTATAGTGAGTCGTATTA
ATTCTGTGCTCGC

>KLK8 v.12
ACCTCACCATGGGACGCCCCCGACCTCGTGCGGCCAAGACGTGGATGTTCCTGCTCTTGCTGGGGGGAGC
CTGGGCAGGAAATACACAGTACGCCTGGGAGACCACAGCCTACAGAATAAAGATGGCCCAGAGCAAGAAA
TACCTGTGGTTCAGTCCATCCCACACCCCTGCTACAACAGCAGCGATGTGGAGGACCACAACCATGATCT
GATGCTTCTTCAACTGCGTGACCAGGCATCCCTGGGGTCCAAAGTGAAGCCCATCAGCCTGGCAGATCAT
TGCACCCAGCCTGGCCAGAAGTGCACCGTCTCAGGCTGGGGCACTGTCACCAGTCCCCGAGGGCGATTCT
GGAGGCCCCCTGGTGTGTGATGGTGCACTCCAGGGCATCACATCCTGGGGCTCAGACCCCTGTGGGAGGT
CCGACAAACCTGGCGTCTATACCAACATCTGCCGCTACCTGGACTGGATCAAGAAGATCATAGGCAGCAA

Commented [CK81]: *KLK8* $F_{(ATG)}$
Commented [CK82]: *KLK8* $F_{(Nested)}$
Commented [CK83]: *KLK8* 2/3altF
Commented [CK84]: Reverse complement of *KLK8* 3/6R
Commented [CK85]: Reverse complement of *KLK8* 6R*
Commented [CK86]: Reverse complement of $R_{(Inner)}$
Commented [CK87]: Reverse complement of $R_{(Outer)}$
Commented [CK88]: *KLK8* $F_{(ATG)}$
Commented [CK89]: *KLK8* $F_{(Nested)}$
Commented [CK90]: *KLK8* 2/N_2F
Commented [CK91]: Reverse complement of *KLK8* N_2/6R
Commented [CK92]: Reverse complement of *KLK8* 6R*
Commented [CK93]: Reverse complement of $R_{(Inner)}$
Commented [CK94]: Reverse complement of $R_{(Outer)}$
Commented [CK95]: *KLK8* $F_{(ATG)}$
Commented [CK96]: *KLK8* $F_{(Nested)}$
Commented [CK97]: *KLK8* 2/N_1F
Commented [CK98]: Reverse complement of *KLK8* N_1/6R
Commented [CK99]: Reverse complement of *KLK8* 6R*
Commented [CK100]: Reverse complement of $R_{(Inner)}$
Commented [CK101]: Reverse complement of $R_{(Outer)}$
Commented [CK102]: *KLK8* $F_{(ATG)}$
Commented [CK103]: *KLK8* $F_{(Nested)}$
Commented [CK104]: *KLK8* 2/6extF
Commented [CK105]: Reverse complement of *KLK8* 6R
Commented [CK106]: Reverse complement of *KLK8* 6R*
Commented [CK107]: Reverse complement of $R_{(Inner)}$
Commented [CK108]: Reverse complement of $R_{(Outer)}$
Commented [CK109]: *KLK8* $F_{(ATG)}$
Commented [CK110]: *KLK8* $F_{(Nested)}$
Commented [CK111]: *KLK8* 2/5trF
Commented [CK112]: Reverse complement of *KLK8* 5/6R
Commented [CK113]: Reverse complement of *KLK8* 6R*
Commented [CK114]: Reverse complement of $R_{(Inner)}$
Commented [CK115]: Reverse complement of $R_{(Outer)}$
Commented [CK116]: *KLK8* $F_{(ATG)}$
Commented [CK117]: *KLK8* $F_{(Nested)}$
Commented [CK118]: *KLK8* 2/4F
Commented [CK119]: Reverse complement of *KLK8* 4/6R
Commented [CK120]: Reverse complement of *KLK8* 6R*

GGGCTGATTCTAGGATAAGCACTAGATCTCCCTTAATAAACTCACAACTCTCTGGTTCAAAAAAAAAAAA
CCTATAGTGAGTCGTATTAATTCTGTGCTCGC

Commented [CK121]: Reverse complement of $R_{(Inner)}$

Commented [CK122]: Reverse complement of $R_{(Outer)}$

>KLK8 v.13
ACCTCACCATGGGACGCCCCCGACCTCGTGCGGCCAAGACGTGGATGTTCCTGCTCTTGCTGGGGGGAGC
CTGGGCAGGACACTCCAGGGCACAGGAGGACAAGGTGCTGGGGGGTCATGAGTGCCAACCCCATTCGCAG
CCTTGGCAGGCGGCCTTGTTCCAGGGCCAGCAACTACTCTGTGGCGGTGTCCTTGTAGGTGGCAACTGGG
TCCTTACAGCTGCCCACTGTAAAAAACCGAAATACACAGTACGCCTGGGAGACCACAGCCTACAGAATAA
AGATGGCCCAGAGCAAGAAATACCTGTGGTTCAGTCCATCCCACACCCCTGCTACAACAGCAGCGATGTG
GAGGACCACAACCATGATCTGATGCTTCTTCAACTGCGTGACCAGGCATCCCTGGGGTCCAAAGTGAAGC
CCATCAGCCTGGCAGATCATTGCACCCAGCCTGGCCAGAAGTGCACCGTCTCAGGCTGGGGCACTGTCAC
CAGTCCCCGAGGGCGATTCTGGAGGCCCCCTGGTGTGTGATGGTGCACTCCAGGGCATCACATCCTGGGG
CTCAGACCCCTGTGGGAGGTCCGACAAACCTGGCCGTCTATACCAACATCTGCCGCTACCTGGACTGGAT
CAAGAAGATCATAGGCAGCAAGGGCTGATTCTAGGATAAGCACTAGATCTCCCTTAATAAACTCACAACT
CTCTGGTTCAAAAAAAAAAAACCTATAGTGAGTCGTATTAATTCTGTGCTCGC

Commented [CK123]: *KLK8* $F_{(ATG)}$

Commented [CK124]: *KLK8* $F_{(Nested)}$

Commented [CK125]: *KLK8* 2/3altF

Commented [CK126]: Reverse complement of *KLK8* 4/6R

Commented [CK127]: Reverse complement of *KLK8* 6R*

Commented [CK128]: Reverse complement of $R_{(Inner)}$

Commented [CK129]: Reverse complement of $R_{(Outer)}$

>KLK8 v.14
ACCTCACCATGGGACGCCCCCGACCTCGTGCGGCCAAGACGTGGATGTTCCTGCTCTTGCTGGGGGGAGC
CTGGGCAGCGTGTGGAAGCCTGGACCTCCTCACTAAGTTGTATGCGGAGAACTTGCCGTGTGTCCATTTG
AACCCACAGTGGCCTTCCCAGCCCTCGCACTGCCCCAGAGGGTGGCGATCCAACCCTCTCCCTCCTGCTG
CAGGACACTCCAGGGCACAGGAGGACAAGGTGCTGGGGGGTCATGAGTGCCAACCCCATTCGCAGCCTTG
GCAGGCGGCCTTGTTCCAGGGCCAGCAACTACTCTGTGGCGGTGTCCTTGTAGGTGGCAACTGGGTCCTT
ACAGCTGCCCACTGTAAAAAACCGAAATACACAGTACGCCTGGGAGACCACAGCCTACAGAATAAAGATG
GCCCAGAGCAAGAAATACCTGTGGTTCAGTCCATCCCACACCCCTGCTACAACAGCAGCGATGTGGAGGA
CCACAACCATGATCTGATGCTTCTTCAACTGCGTGACCAGGCATCCCTGGGGTCCAAAGTGAAGCCCATC
AGCCTGGCAGATCATTGCACCCAGCCTGGCCAGAAGTGCACCGTCTCAGGCTGGGGCACTGTCACCAGTC
CCCGAGGGCGATTCTGGAGGCCCCCTGGTGTGTGATGGTGCACTCCAGGGCATCACATCCTGGGGCTCAG
ACCCCTGTGGGAGGTCCGACAAACCTGGCGTCTATACCAACATCTGCCGCTACCTGGACTGGATCAAGAA
GATCATAGGCAGCAAGGGCTGATTCTAGGATAAGCACTAGATCTCCCTTAATAAACTCACAACTCTCTGG
TTCAAAAAAAAAAAACCTATAGTGAGTCGTATTAATTCTGTGCTCGC

Commented [CK130]: *KLK8* $F_{(ATG)}$

Commented [CK131]: *KLK8* $F_{(Nested)}$

Commented [CK132]: *KLK8* 2/3F

Commented [CK133]: Reverse complement of *KLK8* 4/6R

Commented [CK134]: Reverse complement of *KLK8* 6R*

Commented [CK135]: Reverse complement of $R_{(Inner)}$

Commented [CK136]: Reverse complement of $R_{(Outer)}$

>KLK8 v.15
ACCTCACCATGGGACGCCCCCGACCTCGTGCGGCCAAGACGTGGATGTTCCTGCTCTTGCTGGGGGGAGC
CTGGGCAGAAGTAAAAATCTTTCCCCAGAAGAAGTGTGAGGATGCTTACCCGGGGCAGATCACAGATGGC
ATGGTCTGTGCAGGCAGCAGCAAAGGGGCTGACACGTGCCAGGGGGACCAATCATGCCAAAGAACTGGTAA
AACGCTGGGACAGCAGGAAAAGGGACGTTGTGGACATCTCAGATGCAAGGCTGTTCTCATTCTCCCTGTC
TAGGGCGATTCTGGAGGCCCCCTGGTGTGTGATGGTGCACTCCAGGGCATCACATCCTGGGGCTCAGACC
CCTGTGGGAGGTCCGACAAACCTGGCGTCTATACCAACATCTGCCGCTACCTGGACTGGATCAAGAAGAT
CATAGGCAGCAAGGGCTGATTCTAGGATAAGCACTAGATCTCCCTTAATAAACTCACAACTCTCTGGTTC
AAAAAAAAAAAACCTATAGTGAGTCGTATTAATTCTGTGCTCGC

Commented [CK137]: *KLK8* $F_{(ATG)}$

Commented [CK138]: *KLK8* $F_{(Nested)}$

Commented [CK139]: *KLK8* 2/5trF

Commented [CK140]: Reverse complement of *KLK8* 5/6extR

Commented [CK141]: Reverse complement of *KLK8* 6R*

Commented [CK142]: Reverse complement of $R_{(Inner)}$

Commented [CK143]: Reverse complement of $R_{(Outer)}$

KLK9

>KLK9 v.2
CATGGAGGAGGAAGGAGATGGCATGGCTTACCATAAAGAAGCACTGGACGCCGGGTGCACGTTCCAGGAT
CCAGGTGCCCAGGGGTCATGAAGCTGGGACTCCTCTGTGCTCTGCTCTCTCTGCTGGCAGGGTGACTCTG
GGGGCCCCCTGGTTTGCAATGGAACCTTGGCAGGCGTGGTGTCTGGGGGTGCTGAGCCCTGCTCCAGACC
CCGGCGCCCCGCAGTCTACACCAGCGTATGCCACTACCTTGACTGGATCCAAGAAATCATGGAGAACTGA
GCCCGCGCGCCACGGGGGCACCTTGGAAGACCAAGAGAGGCCGAAGGGCACGGGGTAGGGGGTTCTCGTA
GGGTCCCAGCCTCAATGGTTCCCGCCCTGGACCTCCAGCTGCCCTGACTCCCCTCTGGACACTAAGACTC
CGCCCCTGAGGCTCCGCCCCCTCACGAGGTCAAGCAAGACACAGTCGCGCCCCCTCGGAACGGAGCAGGG
ACACGCCCTTCAGAGCCCGTCTCTATGACGTCACCGACAGCCATCACCTCCTTCTTGGAACAGCACAGCC
TGTGGCTCCGCCCCAAGGAACCACTTACACAAAATAGCTCCGCCCCTCGGAACTTTGCCCAGTGGGACTT
CCCCTCGGGACTCCACCCCTTGTGGCCCCGCCTCCTTCACCAGAGATCTCGCCCCTCGTGATGTCAGGGG
CGCAGTAGCTCCGCCCACGTGGAGCTCGGGCGGTGTAGAGCTCAGCCCCTTGTGGCCCCGTCCTGGGCGT
GTGCTGGGTTTGAATCCTGGCGGAGACCTGGGGGGAAATTGAGGGAGGGTCTGGATACCTTTAGAGCCAA
TGCAACGGATGATTTTTCAGTAAACGCGGGAAACCTCAAAAAAAAAAAACCTATAGTGAGTCGTATTAAT
TCTGTGCTCGC

Commented [CK144]: *KLK9* F$_{(ATG)}$
Commented [CK145]: *KLK9* F$_{(Nested)}$
Commented [CK146]: *KLK9* 1/5F
Commented [CK147]: Reverse complement of *KLK9* 5R
Commented [CK148]: Reverse complement of *KLK9* 5R*
Commented [CK149]: Reverse complement of R$_{(Inner)}$
Commented [CK150]: Reverse complement of R$_{(Outer)}$

>KLK9 v.3
CATGGAGGAGGAAGGAGATGGCATGGCTTACCATAAAGAAGCACTGGACGCCGGGTGCACGTTCCAGGAT
CCAGGTGCCCAGGGGTCATGAAGCTGGGACTCCTCTGTGCTCTGCTCTCTCTGCTGGCAGGGCATGGCTG
GGCAGACACCCGTGCCATCGGGGCCGAGGAATGTCGCCCCAACTCCCAGCCTTGGCAGGCCGGCCTCTTC
CACCTTACTCGGCTCTTCTGTGGGGCGACCCTCATCAGTGACCGCTGGCTGCTCACAGCTGCCCACTGCC
GCAAGCCGGTGACTCTGGGGGCCCCCTGGTTTGCAATGGAACCTTGGCAGGCGTGGTGTCTGGGGGTGCT
GAGCCCTGCTCCAGACCCCGGCGCCCCGCAGTCTACACCAGCGTATGCCACTACCTTGACTGGATCCAAG
AAATCATGGAGAACTGAGCCCGCGCGCCACGGGGGCACCTTGGAAGACCAAGAGAGGCCGAAGGGCACGG
GGTAGGGGGTTCTCGTAGGGTCCCAGCCTCAATGGTTCCCGCCCTGGACCTCCAGCTGCCCTGACTCCCC
TCTGGACACTAAGACTCCGCCCCTGAGGCTCCGCCCCCTCACGAGGTCAAGCAAGACACAGTCGCGCCCC
CTCGGAACGGAGCAGGGACACGCCCTTCAGAGCCCGTCTCTATGACGTCACCGACAGCCATCACCTCCTT
CTTGGAACAGCACAGCCTGTGGCTCCGCCCCAAGGAACCACTTACACAAAATAGCTCCGCCCCTCGGAAC
TTTGCCCAGTGGGACTTCCCCTCGGGACTCCACCCCTTGTGGCCCCGCCTCCTTCACCAGAGATCTCGCC
CCTCGTGATGTCAGGGGCGCAGTAGCTCCGCCCACGTGGAGCTCGGGCGGTGTAGAGCTCAGCCCCTTGT
GGCCCCGTCCTGGGCGTGTGCTGGGTTTGAATCCTGGCGGAGACCTGGGGGGAAATTGAGGGAGGGTCTG
GATACCTTTAGAGCCAATGCAACGGATGATTTTTCAGTAAACGCGGGAAACCTCAAAAAAAAAAAACCTA
TAGTGAGTCGTATTAATTCTGTGCTCGC

Commented [CK151]: *KLK9* F$_{(ATG)}$
Commented [CK152]: *KLK9* F$_{(Nested)}$
Commented [CK153]: *KLK9* 1/2F
Commented [CK154]: Reverse complement of *KLK9* 2/5R
Commented [CK155]: Reverse complement of *KLK9* 5R*
Commented [CK156]: Reverse complement of R$_{(Inner)}$
Commented [CK157]: Reverse complement of R$_{(Outer)}$

>KLK9 v.4
CATGGAGGAGGAAGGAGATGGCATGGCTTACCATAAAGAAGCACTGGACGCCGGGTGCACGTTCCAGGAT
CCAGGTGCCCAGGGGTCATGAAGCTGGGACTCCTCTGTGCTCTGCTCTCTCTGCTGGCAGGTATCTGTGG
GTCCGCCTTGGAGAGCACCACCTCTGGAAATGGGAGGGTCCGGAGCAGCTGTTCCGGGTTACGGACTTCT
TCCCCCACCCTGGCTTCAACAAGGACCTCAGCGCCAATGACCACAATGATGACATCATGCTGATCCGCCT
GCCCAGGCAGGCACGTCTGAGTCCTGCTGTGCAGCCCCTCAACCTCAGCCAGACCTGTGTCTCCCCAGGC
ATGCAGTGTCTCATCTCAGGCTGGGGGGCCGTGTCCAGCCCCAAGGGGTGACTCTGGGGGCCCCCTGGTT
TGCAATGGAACCTTGGCAGGCGTGGTGTCTGGGGGTGCTGAGCCCTGCTCCAGACCCCGGCGCCCCGCAG
TCTACACCAGCGTATGCCACTACCTTGACTGGATCCAAGAAATCATGGAGAACTGAGCCCGCGCGCCACG
GGGGCACCTTGGAAGACCAAGAGAGGCCGAAGGGCACGGGGTAGGGGGTTCTCGTAGGGTCCCAGCCTCA
ATGGTTCCCGCCCTGGACCTCCAGCTGCCCTGACTCCCCTCTGGACACTAAGACTCCGCCCCTGAGGCTC
CGCCCCCTCACGAGGTCAAGCAAGACACAGTCGCGCCCCCTCGGAACGGAGCAGGGACACGCCCTTCAGA
GCCCGTCTCTATGACGTCACCGACAGCCATCACCTCCTTCTTGGAACAGCACAGCCTGTGGCTCCGCCCC
AAGGAACCACTTACACAAAATAGCTCCGCCCCTCGGAACTTTGCCCAGTGGGACTTCCCCTCGGGACTCC
ACCCCTTGTGGCCCCGCCTCCTTCACCAGAGATCTCGCCCCTCGTGATGTCAGGGGCGCAGTAGCTCCGC
CCACGTGGAGCTCGGGCGGTGTAGAGCTCAGCCCCTTGTGGCCCCGTCCTGGGCGTGTGCTGGGTTTGAA
TCCTGGCGGAGACCTGGGGGGAAATTGAGGGAGGGTCTGGATACCTTTAGAGCCAATGCAACGGATGATT
TTTCAGTAAACGCGGGAAACCTCAAAAAAAAAAAACCTATAGTGAGTCGTATTAATTCTGTGCTCGC

Commented [CK158]: *KLK9* F$_{(ATG)}$
Commented [CK159]: *KLK9* F$_{(Nested)}$
Commented [CK160]: *KLK9* 1/3F
Commented [CK161]: Reverse complement of *KLK9* 3/5R
Commented [CK162]: Reverse complement of *KLK9* 5R*
Commented [CK163]: Reverse complement of R$_{(Inner)}$
Commented [CK164]: Reverse complement of R$_{(Outer)}$

>KLK9 v.5

CATGGAGGAGGAAGGAGATGGCATGGCTTACCATAAAGAAGCACTGGACGCCGGGTGCACGTTCCAGGAT
CCAGGTGCCCAGGGGTCATGAAGCTGGGACTCCTCTGTGCTCTGCTCTCTCTGCTGGCAGGTATCTGTGG
GTCCGCCTTGGAGAGCACCACCTCTGGAAATGGGAGGGTCCGGAGCAGCTGTTCCGGGTTACGGACTTCT
TCCCCCACCCTGGCTTCAACAAGGACCTCAGCGCCAATGACCACAATGATGACATCATGCTGATCCGCCT
GCCCAGGCAGGCACGTCTGAGTCCTGCTGTGCAGCCCCTCAACCTCAGCCAGACCTGTGTCTCCCCAGGC
ATGCAGTGTCTCATCTCAGGCTGGGGGGCCGTGTCCAGCCCCAAGGCGCTGTTTCCAGTCACACTGCAGT
GTGCCAACATCAGCATCCTGGAGAACAAACTCTGTCACTGGGCATACCCTGGACACATCTCGGACAGCAT
GCTCTGTGCGGGCCTGTGGGAGGGGGGCCGAGGTTCCTGCCAGGGTGACTCTGGGGGCCCCCTGGTTTGC
AATGGAACCTTGGCAGGCGTGGTGTCTGGGGGTGCTGAGCCCTGCTCCAGACCCCGGCGCCCCGCAGTCT
ACACCAGCGTATGCCACTACCTTGACTGGATCCAAGAAATCATGGAGAACTGAGCCCGCGCGCCACGGGG
GCACCTTGGAAGACCAAGAGAGGCCGAAGGGCACGGGGTAGGGGGTTCTCGTAGGGTCCCAGCCTCAATG
GTTCCCGCCCTGGACCTCCAGCTGCCCTGACTCCCCTCTGGACACTAAGACTCCGCCCCTGAGGCTCCGC
CCCCTCACGAGGTCAAGCAAGACACAGTCGCGCCCCCTCGGAACGGAGCAGGGACACGCCCTTCAGAGCC
CGTCTCTATGACGTCACCGACAGCCATCACCTCCTTCTTGGAACAGCACAGCCTGTGGCTCCGCCCCAAG
GAACCACTTACACAAAATAGCTCCGCCCCTCGGAACTTTGCCCAGTGGGACTTCCCCTCGGGACTCCACC
CCTTGTGGCCCCGCCTCCTTCACCAGAGATCTCGCCCCTCGTGATGTCAGGGGCGCAGTAGCTCCGCCCA
CGTGGAGCTCGGGCGGTGTAGAGCTCAGCCCCTTGTGGCCCCGTCCTGGGCGTGTGCTGGGTTTGAATCC
TGGCGGAGACCTGGGGGGAAATTGAGGGAGGGTCTGGATACCTTTAGAGCCAATGCAACGGATGATTTTT
CAGTAAACGCGGGAAACCTCAAAAAAAAAAAACCTATAGTGAGTCGTATTAATTCTGTGCTCGC

Commented [CK165]: *KLK9* $F_{(ATG)}$

Commented [CK166]: *KLK9* $F_{(Nested)}$

Commented [CK167]: *KLK9* 1/3F

Commented [CK168]: Reverse complement of *KLK9* 4/5R

Commented [CK169]: Reverse complement of *KLK9* 5R*

Commented [CK170]: Reverse complement of $R_{(Inner)}$

Commented [CK171]: Reverse complement of $R_{(Outer)}$

> KLK9 v.6

CATGGAGGAGGAAGGAGATGGCATGGCTTACCATAAAGAAGCACTGGACGCCGGGTGCACGTTCCAGGAT
CCAGGTGCCCAGGGGTCATGAAGCTGGGACTCCTCTGTGCTCTGCTCTCTCTGCTGGCAGAGTCTCCCTC
TATCTCCCAGGCTGGAGTGCAGTGGCACAATCTCAGCTCACTGCAACCTCTGCCTCTCGGGTTCAAGCAA
TTCTCCTGCCTCAGCCTCCTGAGTAGCTGGGATTACAGGTATCTGTGGGTCCGCCTTGGAGAGCACCACC
TCTGGAAATGGGAGGGTCCGGAGCAGCTGTTCCGGGTTACGGACTTCTTCCCCCACCCTGGCTTCAACAA
GGACCTCAGCGCCAATGACCACAATGATGACATCATGCTGATCCGCCTGCCCAGGCAGGCACGTCTGAGT
CCTGCTGTGCAGCCCCTCAACCTCAGCCAGACCTGTGTCTCCCCAGGCATGCAGTGTCTCATCTCAGGCT
GGGGGGCCGTGTCCAGCCCCAAGGGGTGACTCTGGGGGCCCCCTGGTTTGCAATGGAACCTTGGCAGGCG
TGGTGTCTGGGGGTGCTGAGCCCTGCTCCAGACCCCGGCGCCCCGCAGTCTACACCAGCGTATGCCACTA
CCTTGACTGGATCCAAGAAATCATGGAGAACTGAGCCCGCGCGCCACGGGGGCACCTTGGAAGACCAAGA
GAGGCCGAAGGGCACGGGGTAGGGGGTTCTCGTAGGGTCCCAGCCTCAATGGTTCCCGCCCTGGACCTCC
AGCTGCCCTGACTCCCCTCTGGACACTAAGACTCCGCCCCTGAGGCTCCGCCCCCTCACGAGGTCAAGCA
AGACACAGTCGCGCCCCCTCGGAACGGAGCAGGGACACGCCCTTCAGAGCCCGTCTCTATGACGTCACCG
ACAGCCATCACCTCCTTCTTGGAACAGCACAGCCTGTGGCTCCGCCCCAAGGAACCACTTACACAAAATA
GCTCCGCCCCTCGGAACTTTGCCCAGTGGGACTTCCCCTCGGGACTCCACCCCTTGTGGCCCCGCCTCCT
TCACCAGAGATCTCGCCCCTCGTGATGTCAGGGGCGCAGTAGCTCCGCCCACGTGGAGCTCGGGCGGTGT
AGAGCTCAGCCCCTTGTGGCCCCGTCCTGGGCGTGTGCTGGGTTTGAATCCTGGCGGAGACCTGGGGGGA
AATTGAGGGAGGGTCTGGATACCTTTAGAGCCAATGCAACGGATGATTTTTCAGTAAACGCGGGAAACCT
CAAAAAAAAAAAACCTATAGTGAGTCGTATTAATTCTGTGCTCGC

Commented [CK172]: *KLK9* $F_{(ATG)}$

Commented [CK173]: *KLK9* $F_{(Nested)}$

Commented [CK174]: *KLK9* 1/NF

Commented [CK175]: Reverse complement of *KLK9* 3/5R

Commented [CK176]: Reverse complement of *KLK9* 5R*

Commented [CK177]: Reverse complement of $R_{(Inner)}$

Commented [CK178]: Reverse complement of $R_{(Outer)}$

>KLK9 v.7

CATGGAGGAGGAAGGAGATGGCATGGCTTACCATAAAGAAGCACTGGACGCCGGGTGCACGTTCCAGGAT
CCAGGTGCCCAGGGGTCATGAAGCTGGGACTCCTCTGTGCTCTGCTCTCTCTGCTGGCAGAGTCTCCCTC
TATCTCCCAGGCTGGAGTGCAGTGGCACAATCTCAGCTCACTGCAACCTCTGCCTCTCGGGTTCAAGCAA
TTCTCCTGCCTCAGCCTCCTGAGTAGCTGGGATTACAGGTATCTGTGGGTCCGCCTTGGAGAGCACCACC
TCTGGAAATGGGAGGGTCCGGAGCAGCTGTTCCGGGTTACGGACTTCTTCCCCCACCCTGGCTTCAACAA
GGACCTCAGCGCCAATGACCACAATGATGACATCATGCTGATCCGCCTGCCCAGGCAGGCACGTCTGAGT
CCTGCTGTGCAGCCCCTCAACCTCAGCCAGACCTGTGTCTCCCCAGGCATGCAGTGTCTCATCTCAGGCT
GGGGGGCCGTGTCCAGCCCCAAGGCGCTGTTTCCAGTCACACTGCAGTGTGCCAACATCAGCATCCTGGA
GAACAAACTCTGTCACTGGGCATACCCTGGACACATCTCGGACAGCATGCTCTGTGCGGGCCTGTGGGAG
GGGGGCCGAGGTTCCTGCCAGGGTGACTCTGGGGGCCCCCTGGTTTGCAATGGAACCTTGGCAGGCGTGG
TGTCTGGGGGTGCTGAGCCCTGCTCCAGACCCCGGCGCCCCGCAGTCTACACCAGCGTATGCCACTACCT
TGACTGGATCCAAGAAATCATGGAGAACTGAGCCCGCGCGCCACGGGGGCACCTTGGAAGACCAAGAGAG

Commented [CK179]: *KLK9* $F_{(ATG)}$

Commented [CK180]: *KLK9* $F_{(Nested)}$

Commented [CK181]: *KLK9* 1/NF

Commented [CK182]: Reverse complement of *KLK9* 4/5R

Commented [CK183]: Reverse complement of *KLK9* 5R*

GCCGAAGGGCACGGGGTAGGGGGTTCTCGTAGGGTCCCAGCCTCAATGGTTCCCGCCCTGGACCTCCAGC
TGCCCTGACTCCCCTCTGGACACTAAGACTCCGCCCCTGAGGCTCCGCCCCCTCACGAGGTCAAGCAAGA
CACAGTCGCGCCCCCTCGGAACGGAGCAGGGACACGCCCTTCAGAGCCCGTCTCTATGACGTCACCGACA
GCCATCACCTCCTTCTTGGAACAGCACAGCCTGTGGCTCCGCCCCAAGGAACCACTTACACAAAATAGCT
CCGCCCCTCGGAACTTTGCCCAGTGGGACTTCCCCTCGGGACTCCACCCCTTGTGGCCCCGCCTCCTTCA
CCAGAGATCTCGCCCCTCGTGATGTCAGGGGCGCAGTAGCTCCGCCCACGTGGAGCTCGGGCGGTGTAGA
GCTCAGCCCCTTGTGGCCCCGTCCTGGGCGTGTGCTGGGTTTGAATCCTGGCGGAGACCTGGGGGGAAAT
TGAGGGAGGGTCTGGATACCTTTAGAGCCAATGCAACGGATGATTTTTCAGTAAACGCGGGAAACCTCAA
AAAAAAAAAACCTATAGTGAGTCGTATTAATTCTGTGCTCGC

>KLK9 v.8
CATGGAGGAGGAAGGAGATGGCATGGCTTACCATAAAGAAGCACTGGACGCCGGGTGCACGTTCCAGGAT
CCAGGTGCCCAGGGGTCATGAAGCTGGGACTCCTCTGTGCTCTGCTCTCTCTGCTGGCAGGGCATGGCTG
GGCAGACACCCGTGCCATCGGGGCCGAGGAATGTCGCCCCAACTCCCAGCCTTGGCAGGCCGGCCTCTTC
CACCTTACTCGGCTCTTCTGTGGGGCGACCCTCATCAGTGACCGCTGGCTGCTCACAGCTGCCCACTGCC
GCAAGCCGCTGGAGTGCAGTGGCACAATCTCAGCTCACTGCAACCTCTGCCTCTCGGGTTCAAGCAATTC
TCCTGCCTCAGCCTCCTGAGTAGCTGGGATTACAGGTATCTGTGGGTCCGCCTTGGAGAGCACCACCTCT
GGAAATGGGAGGGTCCGGAGCAGCTGTTCCGGGTTACGGACTTCTTCCCCCACCCTGGCTTCAACAAGGA
CCTCAGCGCCAATGACCACAATGATGACATCATGCTGATCCGCCTGCCCAGGCAGGCACGTCTGAGTCCT
GCTGTGCAGCCCCTCAACCTCAGCCAGACCTGTGTCTCCCCAGGCATGCAGTGTCTCATCTCAGGCTGGG
GGGCCGTGTCCAGCCCCAAGGGGTGACTCTGGGGGCCCCCTGGTTTGCAATGGAACCTTGGCAGGCGTGG
TGTCTGGGGGTGCTGAGCCCTGCTCCAGACCCCGGCGCCCCGCAGTCTACACCAGCGTATGCCACTACCT
TGACTGGATCCAAGAAATCATGGAGAACTGAGCCCGCGCGCCACGGGGGCACCTTGGAAGACCAAGAGAG
GCCGAAGGGCACGGGGTAGGGGGTTCTCGTAGGGTCCCAGCCTCAATGGTTCCCGCCCTGGACCTCCAGC
TGCCCTGACTCCCCTCTGGACACTAAGACTCCGCCCCTGAGGCTCCGCCCCCTCACGAGGTCAAGCAAGA
CACAGTCGCGCCCCCTCGGAACGGAGCAGGGACACGCCCTTCAGAGCCCGTCTCTATGACGTCACCGACA
GCCATCACCTCCTTCTTGGAACAGCACAGCCTGTGGCTCCGCCCCAAGGAACCACTTACACAAAATAGCT
CCGCCCCTCGGAACTTTGCCCAGTGGGACTTCCCCTCGGGACTCCACCCCTTGTGGCCCCGCCTCCTTCA
CCAGAGATCTCGCCCCTCGTGATGTCAGGGGCGCAGTAGCTCCGCCCACGTGGAGCTCGGGCGGTGTAGA
GCTCAGCCCCTTGTGGCCCCGTCCTGGGCGTGTGCTGGGTTTGAATCCTGGCGGAGACCTGGGGGGAAAT
TGAGGGAGGGTCTGGATACCTTTAGAGCCAATGCAACGGATGATTTTTCAGTAAACGCGGGAAACCTCAA
AAAAAAAAAACCTATAGTGAGTCGTATTAATTCTGTGCTCGC

>KLK9 v.9
CATGGAGGAGGAAGGAGATGGCATGGCTTACCATAAAGAAGCACTGGACGCCGGGTGCACGTTCCAGGAT
CCAGGTGCCCAGGGGTCATGAAGCTGGGACTCCTCTGTGCTCTGCTCTCTCTGCTGGCAGGGCATGGCTG
GGCAGACACCCGTGCCATCGGGGCCGAGGAATGTCGCCCCAACTCCCAGCCTTGGCAGGCCGGCCTCTTC
CACCTTACTCGGCTCTTCTGTGGGGCGACCCTCATCAGTGACCGCTGGCTGCTCACAGCTGCCCACTGCC
GCAAGCCGCTGGAGTGCAGTGGCACAATCTCAGCTCACTGCAACCTCTGCCTCTCGGGTTCAAGCAATTC
TCCTGCCTCAGCCTCCTGAGTAGCTGGGATTACAGGTATCTGTGGGTCCGCCTTGGAGAGCACCACCTCT
GGAAATGGGAGGGTCCGGAGCAGCTGTTCCGGGTTACGGACTTCTTCCCCCACCCTGGCTTCAACAAGGA
CCTCAGCGCCAATGACCACAATGATGACATCATGCTGATCCGCCTGCCCAGGCAGGCACGTCTGAGTCCT
GCTGTGCAGCCCCTCAACCTCAGCCAGACCTGTGTCTCCCCAGGCATGCAGTGTCTCATCTCAGGCTGGG
GGGCCGTGTCCAGCCCCAAGGCGCTGTTTCCAGTCACACTGCAGTGTGCCAACATCAGCATCCTGGAGAA
CAAACTCTGTCACTGGGCATACCCTGGACACATCTCGGACAGCATGCTCTGTGCGGGCCTGTGGGAGGGG
GGCCGAGGTTCCTGCCAGGGTGACTCTGGGGGCCCCCTGGTTTGCAATGGAACCTTGGCAGGCGTGGTGT
CTGGGGGTGCTGAGCCCTGCTCCAGACCCCGGCGCCCCGCAGTCTACACCAGCGTATGCCACTACCTTGA
CTGGATCCAAGAAATCATGGAGAACTGAGCCCGCGCGCCACGGGGGCACCTTGGAAGACCAAGAGAGGCC
GAAGGGCACGGGGTAGGGGGTTCTCGTAGGGTCCCAGCCTCAATGGTTCCCGCCCTGGACCTCCAGCTGC
CCTGACTCCCCTCTGGACACTAAGACTCCGCCCCTGAGGCTCCGCCCCCTCACGAGGTCAAGCAAGACAC
AGTCGCGCCCCCTCGGAACGGAGCAGGGACACGCCCTTCAGAGCCCGTCTCTATGACGTCACCGACAGCC
ATCACCTCCTTCTTGGAACAGCACAGCCTGTGGCTCCGCCCCAAGGAACCACTTACACAAAATAGCTCCG
CCCCTCGGAACTTTGCCCAGTGGGACTTCCCCTCGGGACTCCACCCCTTGTGGCCCCGCCTCCTTCACCA
GAGATCTCGCCCCTCGTGATGTCAGGGGCGCAGTAGCTCCGCCCACGTGGAGCTCGGGCGGTGTAGAGCT
CAGCCCCTTGTGGCCCCGTCCTGGGCGTGTGCTGGGTTTGAATCCTGGCGGAGACCTGGGGGGAAATTGA

Commented [CK184]: Reverse complement of $R_{(Inner)}$

Commented [CK185]: Reverse complement of $R_{(Outer)}$

Commented [CK186]: *KLK9* $F_{(ATG)}$

Commented [CK187]: *KLK9* $F_{(Nested)}$

Commented [CK188]: *KLK9* 2/NtrF

Commented [CK189]: Reverse complement of *KLK9* 3/5R

Commented [CK190]: Reverse complement of *KLK9* 5R*

Commented [CK191]: Reverse complement of $R_{(Inner)}$

Commented [CK192]: Reverse complement of $R_{(Outer)}$

Commented [CK193]: *KLK9* $F_{(ATG)}$

Commented [CK194]: *KLK9* $F_{(Nested)}$

Commented [CK195]: *KLK9* 2/NtrF

Commented [CK196]: Reverse complement of *KLK9* 4/5R

Commented [CK197]: Reverse complement of *KLK9* 5R*

GGGAGGGTCTGGATACCTTTAGAGCCAATGCAACGGATGATTTTTCAGTAAACGCGGGAAACCTCAAAAA
AAAAAAACCTATAGTGAGTCGTATTAATTCTGTGCTCGC

>KLK9 v.10
CATGGAGGAGGAAGGAGATGGCATGGCTTACCATAAAGAAGCACTGGACGCCGGGTGCACGTTCCAGGAT
CCAGGTGCCCAGGGGTCATGAAGCTGGGACTCCTCTGTGCTCTGCTCTCTCTGCTGGCAGGGCATGGCTG
GGCAGACACCCGTGCCATCGGGGCCGAGGAATGTCGCCCCAACTCCCAGCCTTGGCAGGCCGGCCTCTTC
CACCTTACTCGGCTCTTCTGTGGGGCGACCCTCATCAGTGACCGCTGGCTGCTCACAGCTGCCCACTGCC
GCAAGCCAGTCTCCCTCTATCTCCCAGGCTGGAGTGCAGTGGCACAATCTCAGCTCACTGCAACCTCTGC
CTCTCGGGTTCAAGCAATTCTCCTGCCTCAGCCTCCTGAGTAGCTGGGATTACAGGTATCTGTGGGTCCG
CCTTGGAGAGCACCACCTCTGGAAATGGGAGGGTCCGGAGCAGCTGTTCCGGGTTACGGACTTCTTCCCC
CACCCTGGCTTCAACAAGGACCTCAGCGCCAATGACCACAATGATGACATCATGCTGATCCGCCTGCCCA
GGCAGGCACGTCTGAGTCCTGCTGTGCAGCCCCTCAACCTCAGCCAGACCTGTGTCTCCCCAGGCATGCA
GTGTCTCATCTCAGGCTGGGGGGCCGTGTCCAGCCCCAAGGGGTGACTCTGGGGGCCCCCTGGTTTGCAA
TGGAACCTTGGCAGGCGTGGTGTCTGGGGGTGCTGAGCCCTGCTCCAGACCCCGGCGCCCCGCAGTCTAC
ACCAGCGTATGCCACTACCTTGACTGGATCCAAGAAATCATGGAGAACTGAGCCCGCGCGCCACGGGGGC
ACCTTGGAAGACCAAGAGAGGCCGAAGGGCACGGGGTAGGGGGTTCTCGTAGGGTCCCAGCCTCAATGGT
TCCCGCCCTGGACCTCCAGCTGCCCTGACTCCCCTCTGGACACTAAGACTCCGCCCCTGAGGCTCCGCCC
CCTCACGAGGTCAAGCAAGACACAGTCGCGCCCCCTCGGAACGGAGCAGGGACACGCCCTTCAGAGCCCG
TCTCTATGACGTCACCGACAGCCATCACCTCCTTCTTGGAACAGCACAGCCTGTGGCTCCGCCCCAAGGA
ACCACTTACACAAAATAGCTCCGCCCCTCGGAACTTTGCCCAGTGGGACTTCCCCTCGGGACTCCACCCC
TTGTGGCCCCGCCTCCTTCACCAGAGATCTCGCCCCTCGTGATGTCAGGGGCGCAGTAGCTCCGCCCACG
TGGAGCTCGGGCGGTGTAGAGCTCAGCCCCTTGTGGCCCCGTCCTGGGCGTGTGCTGGGTTTGAATCCTG
GCGGAGACCTGGGGGGAAATTGAGGGAGGGTCTGGATACCTTTAGAGCCAATGCAACGGATGATTTTTCA
GTAAACGCGGGAAACCTCAAAAAAAAAAAACCTATAGTGAGTCGTATTAATTCTGTGCTCGC

>KLK9 v.11
CATGGAGGAGGAAGGAGATGGCATGGCTTACCATAAAGAAGCACTGGACGCCGGGTGCACGTTCCAGGAT
CCAGGTGCCCAGGGGTCATGAAGCTGGGACTCCTCTGTGCTCTGCTCTCTCTGCTGGCAGGGCATGGCTG
GGCAGACACCCGTGCCATCGGGGCCGAGGAATGTCGCCCCAACTCCCAGCCTTGGCAGGCCGGCCTCTTC
CACCTTACTCGGCTCTTCTGTGGGGCGACCCTCATCAGTGACCGCTGGCTGCTCACAGCTGCCCACTGCC
GCAAGCCAGTCTCCCTCTATCTCCCAGGCTGGAGTGCAGTGGCACAATCTCAGCTCACTGCAACCTCTGC
CTCTCGGGTTCAAGCAATTCTCCTGCCTCAGCCTCCTGAGTAGCTGGGATTACAGGTATCTGTGGGTCCG
CCTTGGAGAGCACCACCTCTGGAAATGGGAGGGTCCGGAGCAGCTGTTCCGGGTTACGGACTTCTTCCCC
CACCCTGGCTTCAACAAGGACCTCAGCGCCAATGACCACAATGATGACATCATGCTGATCCGCCTGCCCA
GGCAGGCACGTCTGAGTCCTGCTGTGCAGCCCCTCAACCTCAGCCAGACCTGTGTCTCCCCAGGCATGCA
GTGTCTCATCTCAGGCTGGGGGGCCGTGTCCAGCCCCAAGGCGCTGTTTCCAGTCACACTGCAGTGTGCC
AACATCAGCATCCTGGAGAACAAACTCTGTCACTGGGCATACCCTGGACACATCTCGGACAGCATGCTCT
GTGCGGGCCTGTGGGAGGGGGGCCGAGGTTCCTGCCAGGGTGACTCTGGGGGCCCCCTGGTTTGCAATGG
AACCTTGGCAGGCGTGGTGTCTGGGGGTGCTGAGCCCTGCTCCAGACCCCGGCGCCCCGCAGTCTACACC
AGCGTATGCCACTACCTTGACTGGATCCAAGAAATCATGGAGAACTGAGCCCGCGCGCCACGGGGGCACC
TTGGAAGACCAAGAGAGGCCGAAGGGCACGGGGTAGGGGGTTCTCGTAGGGTCCCAGCCTCAATGGTTCC
CGCCCTGGACCTCCAGCTGCCCTGACTCCCCTCTGGACACTAAGACTCCGCCCCTGAGGCTCCGCCCCCT
CACGAGGTCAAGCAAGACACAGTCGCGCCCCCTCGGAACGGAGCAGGGACACGCCCTTCAGAGCCCGTCT
CTATGACGTCACCGACAGCCATCACCTCCTTCTTGGAACAGCACAGCCTGTGGCTCCGCCCCAAGGAACC
ACTTACACAAAATAGCTCCGCCCCTCGGAACTTTGCCCAGTGGGACTTCCCCTCGGGACTCCACCCCTTG
TGGCCCCGCCTCCTTCACCAGAGATCTCGCCCCTCGTGATGTCAGGGGCGCAGTAGCTCCGCCCACGTGG
AGCTCGGGCGGTGTAGAGCTCAGCCCCTTGTGGCCCCGTCCTGGGCGTGTGCTGGGTTTGAATCCTGGCG
GAGACCTGGGGGGAAATTGAGGGAGGGTCTGGATACCTTTAGAGCCAATGCAACGGATGATTTTTCAGTA
AACGCGGGAAACCTCAAAAAAAAAAAACCTATAGTGAGTCGTATTAATTCTGTGCTCGC

Commented [CK198]: Reverse complement of $R_{(Inner)}$

Commented [CK199]: Reverse complement of $R_{(Outer)}$

Commented [CK200]: *KLK9* $F_{(ATG)}$

Commented [CK201]: *KLK9* $F_{(Nested)}$

Commented [CK202]: *KLK9* 2/NF

Commented [CK203]: Reverse complement of *KLK9* 3/5R

Commented [CK204]: Reverse complement of *KLK9* 5R*

Commented [CK205]: Reverse complement of $R_{(Inner)}$

Commented [CK206]: Reverse complement of $R_{(Outer)}$

Commented [CK207]: *KLK9* $F_{(ATG)}$

Commented [CK208]: *KLK9* $F_{(Nested)}$

Commented [CK209]: *KLK9* 2/NF

Commented [CK210]: Reverse complement of *KLK9* 4/5R

Commented [CK211]: Reverse complement of *KLK9* 5R*

Commented [CK212]: Reverse complement of $R_{(Inner)}$

Commented [CK213]: Reverse complement of $R_{(Outer)}$

Original agarose gel pictures

Original agarose gel pictures showing the results of expression analysis of each novel splice variant in a panel of cDNAs corresponding to distinct cancerous and normal human tissues. cDNAs derived from established cell lines were mixed to generate cDNA pools, each one representing a different human tissue. With regard to *KLK9* variants, only tissues with detectable expression of at least one novel splice variant are shown.

KLK5 v.4

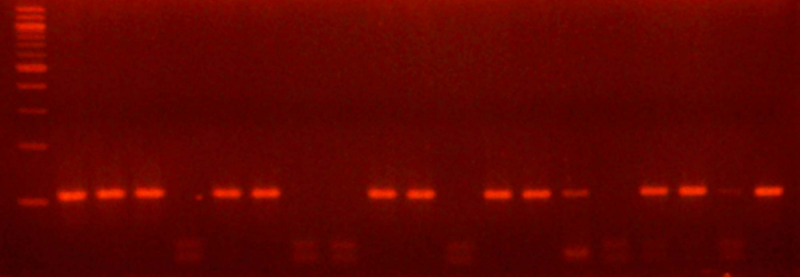

KLK5 v.5

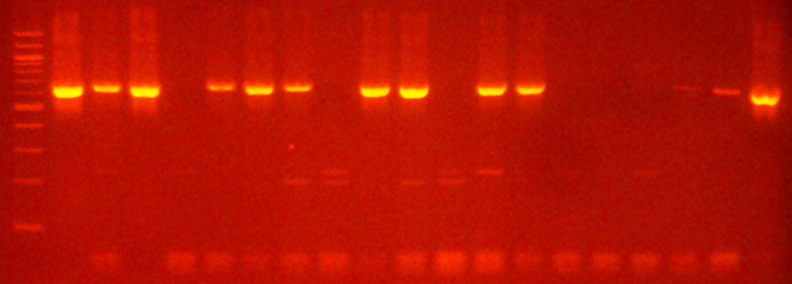

***KLK5* v.6**

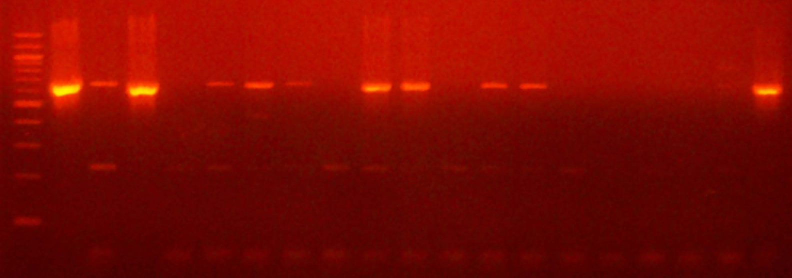

***KLK6* v.6**

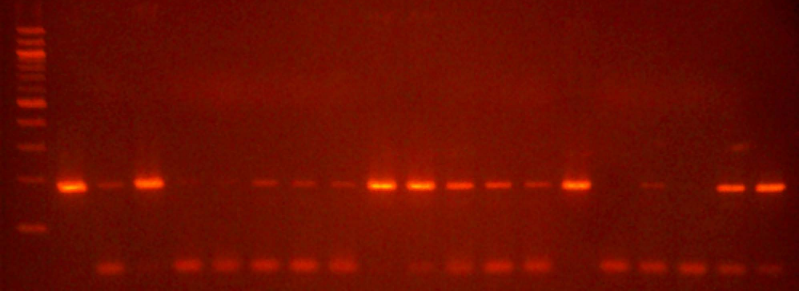

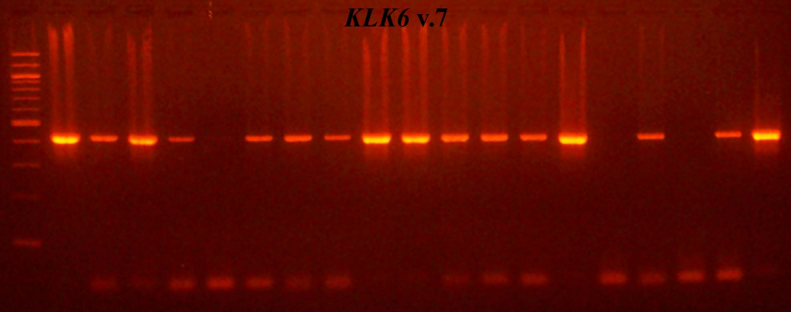
KLK6 v.7

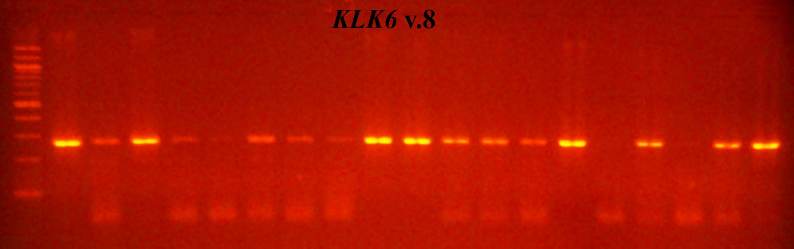

KLK6 v.8

KLK6 v.9

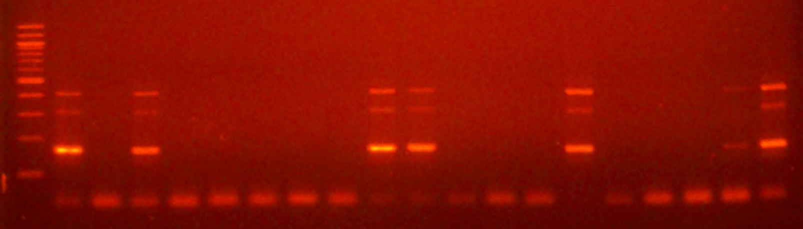

KLK6 v.10

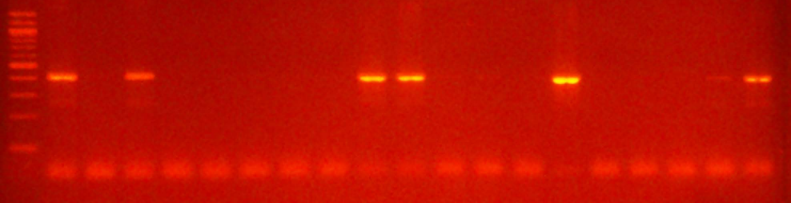

KLK6 v.11

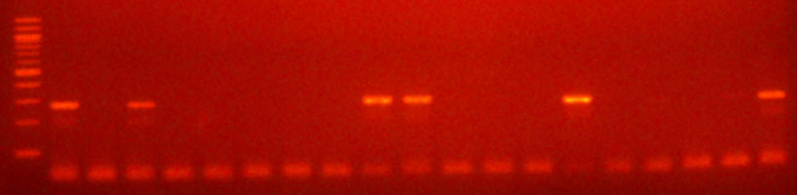

KLK7 v.5

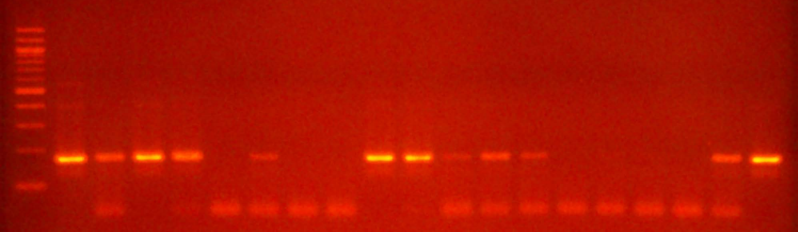

KLK7 v.6

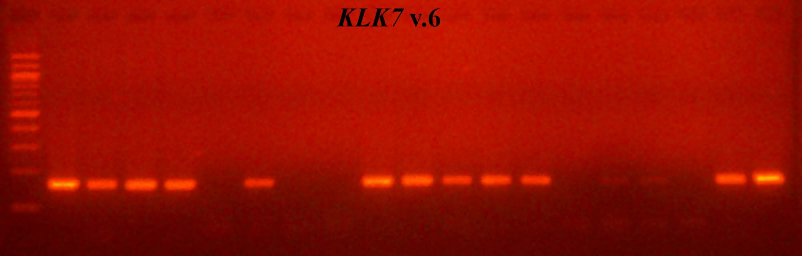

***KLK8* v.7**

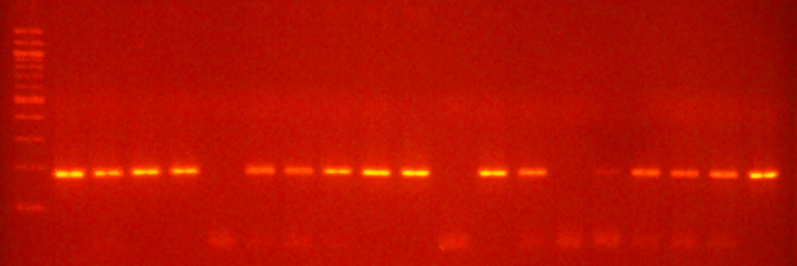

***KLK8* v.8**

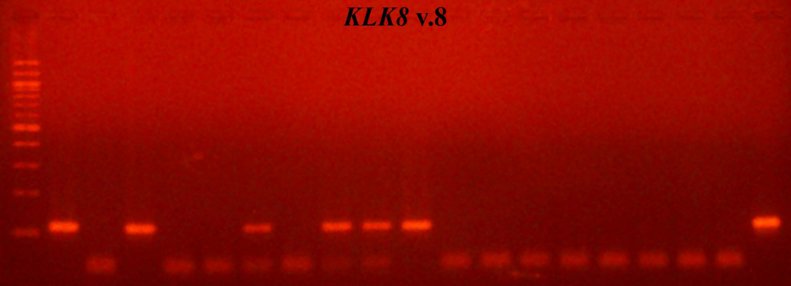

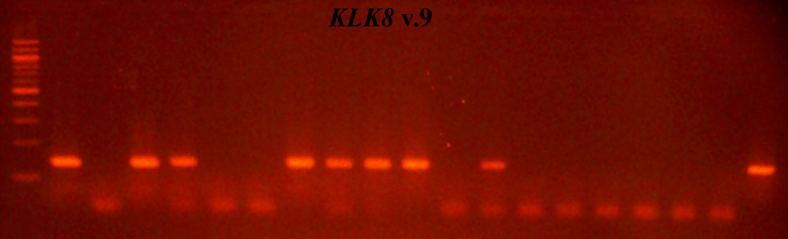
KLK8 v.9

***KLK8* v.10**

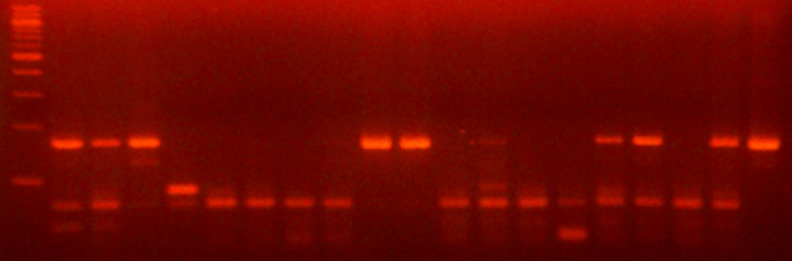

***KLK8* v.11**

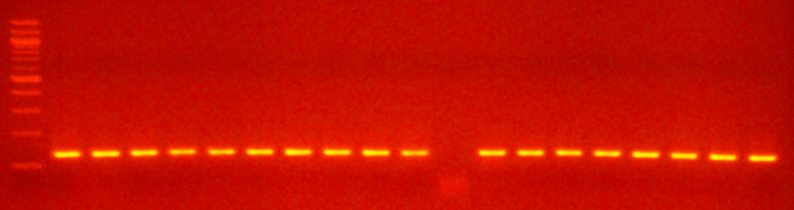

KLK8 v.12

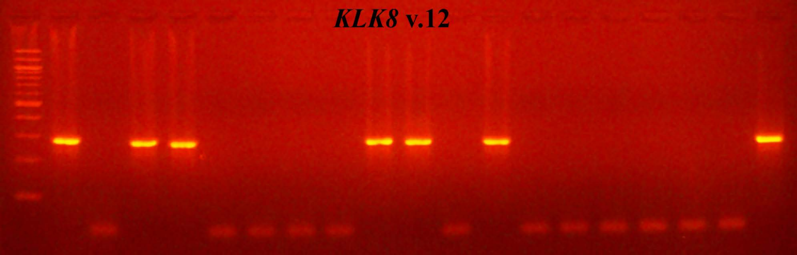

KLK8 v.13

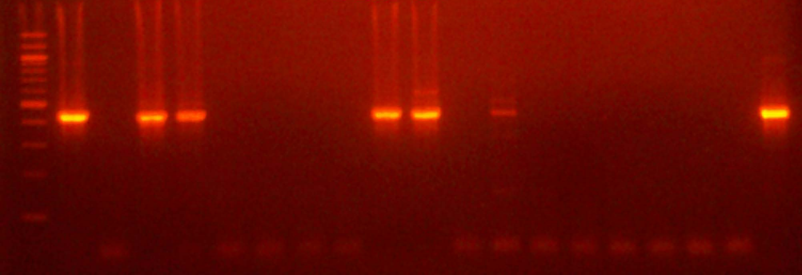

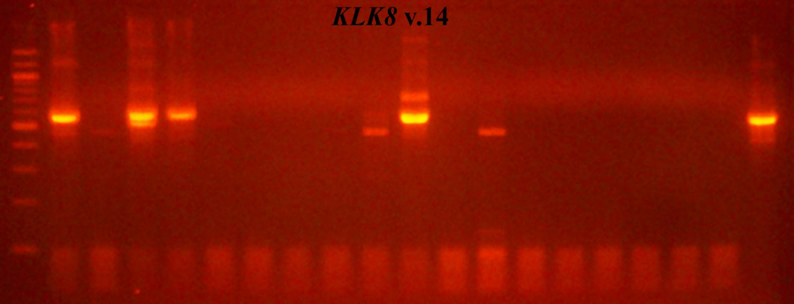
KLK8 v.14

KLK8 v.15

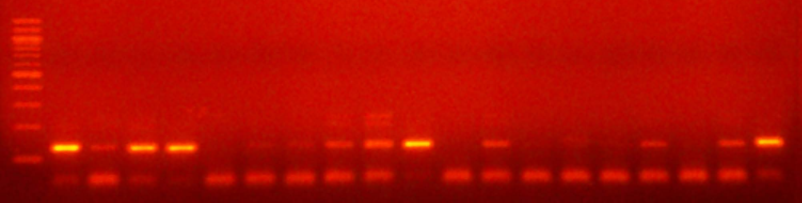

KLK9 v.2

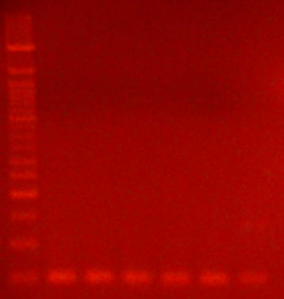

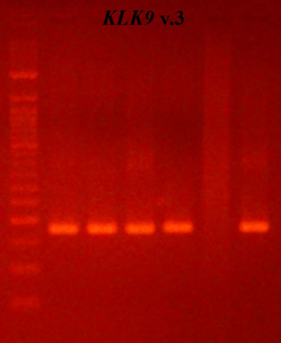
KLK9 v.3

***KLK9* v.4**

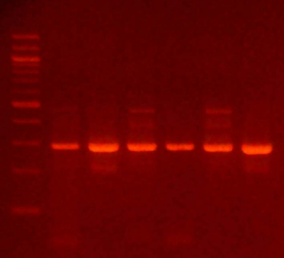

***KLK9* v.5**

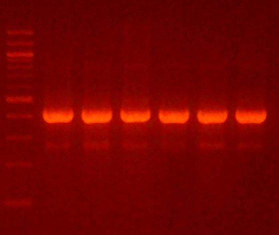

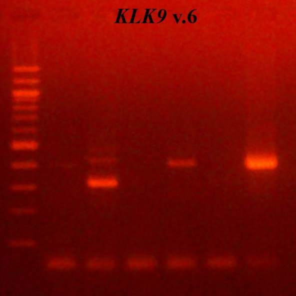
KLK9 v.6

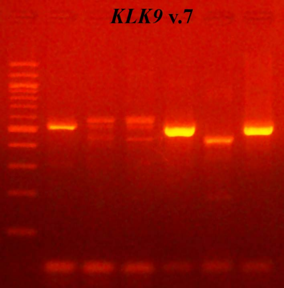
KLK9 v.7

KLK9 v.8

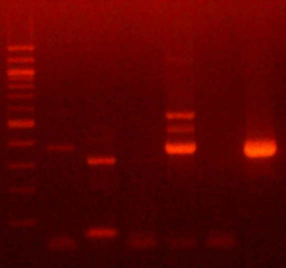

KLK9 v.9

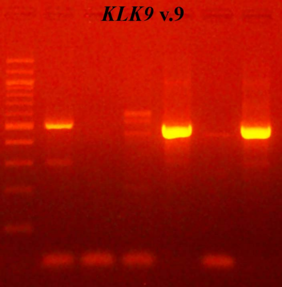

***KLK9* v.10**

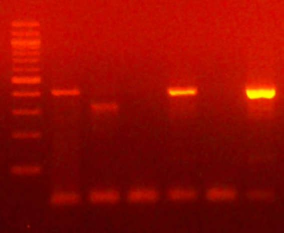

KLK9 v.11

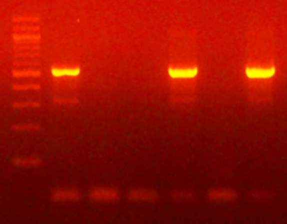

Sanger sequencing results

The nested RT-PCR products (>200 bp) of the novel splice variants of *KLK5*, *KLK6*, *KLK7*, *KLK8*, and *KLK9* were sequenced in both directions. Sanger sequencing results are presented in the following pages.

Clip. 1 BQ 20 WL 10 Sequence: KLK5 V5 Forward

Clipped length:	626	Samples:	8177	Quality: 0 - 9	Page: 1 / 2
Left clip:	13	Bases:	683	10 - 19	22.09.2016
Right clip:	638	Average spacing:	12.0	20 - 29	
Avg. qual. in clip.:	52.51	Average quality >=	10: 23, 20: 13, 30: 600	>= 30	

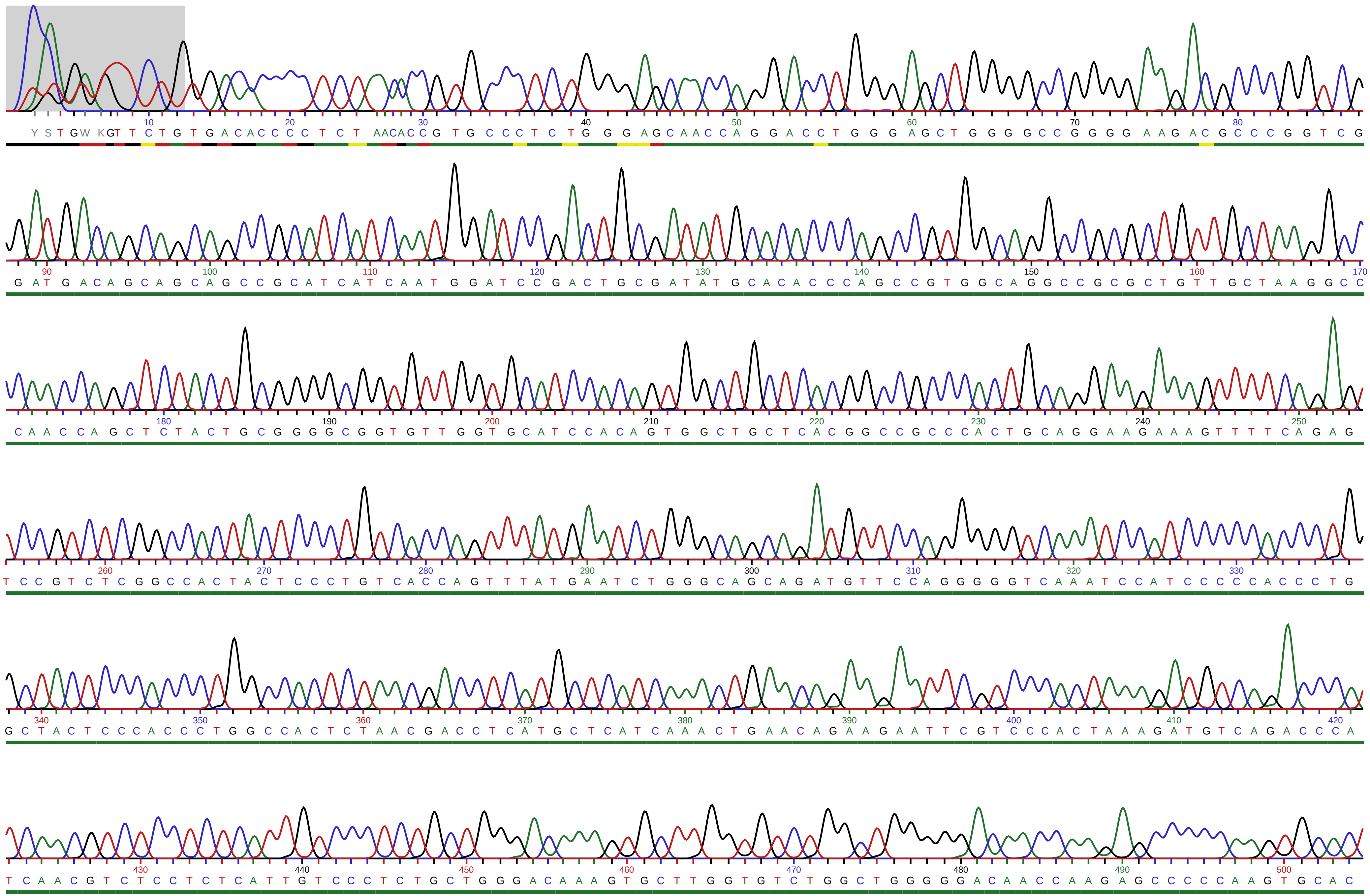

Clip. 1 BQ 20 WL 10 Sequence: KLK5 V5 Forward

Clipped length:	626	Samples:	8177
Left clip:	13	Bases:	683
Right clip:	638	Average spacing:	12.0
Avg. qual. in clip.:	52.51	Average quality >=	10: 23, 20: 13, 30: 600

Quality: 0 - 9, 10 - 19, 20 - 29, >= 30

22.09.2016

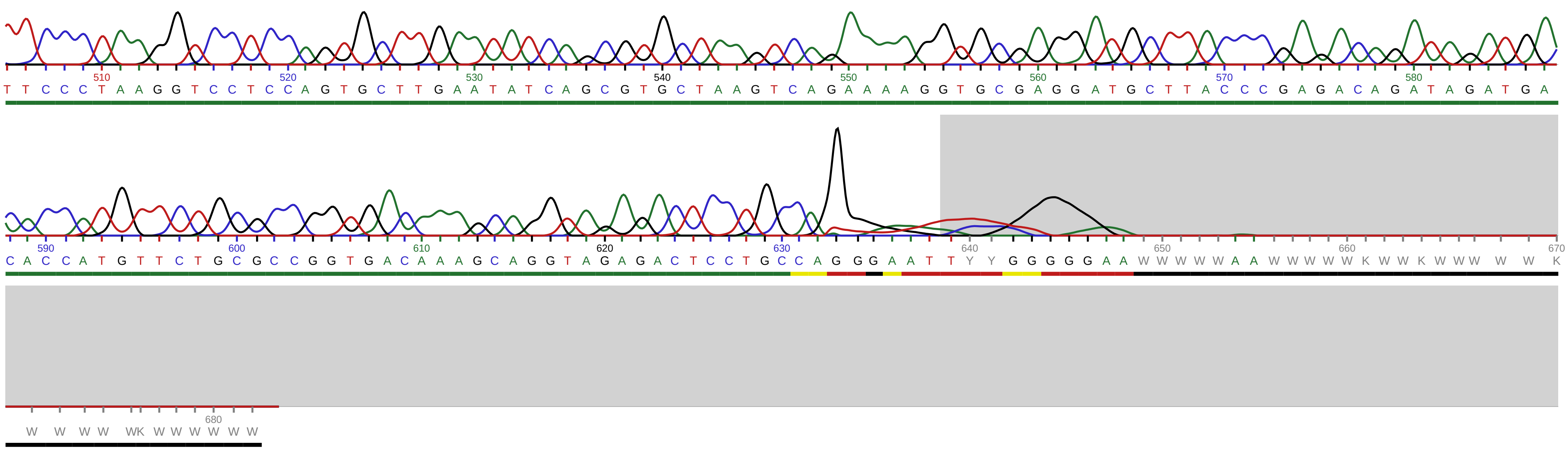

Clip. 1 BQ 20 WL 10 Sequence: KLK5 V5 Reverse

Clipped length: 621
Left clip: 16
Right clip: 636
Avg. qual. in clip.: 51.87

Samples: 12961
Bases: 653
Average spacing: 20.0
Average quality >= 10: 10, 20: 11, 30: 598

Quality: 0 - 9; 10 - 19; 20 - 29; >= 30

26.05.2017

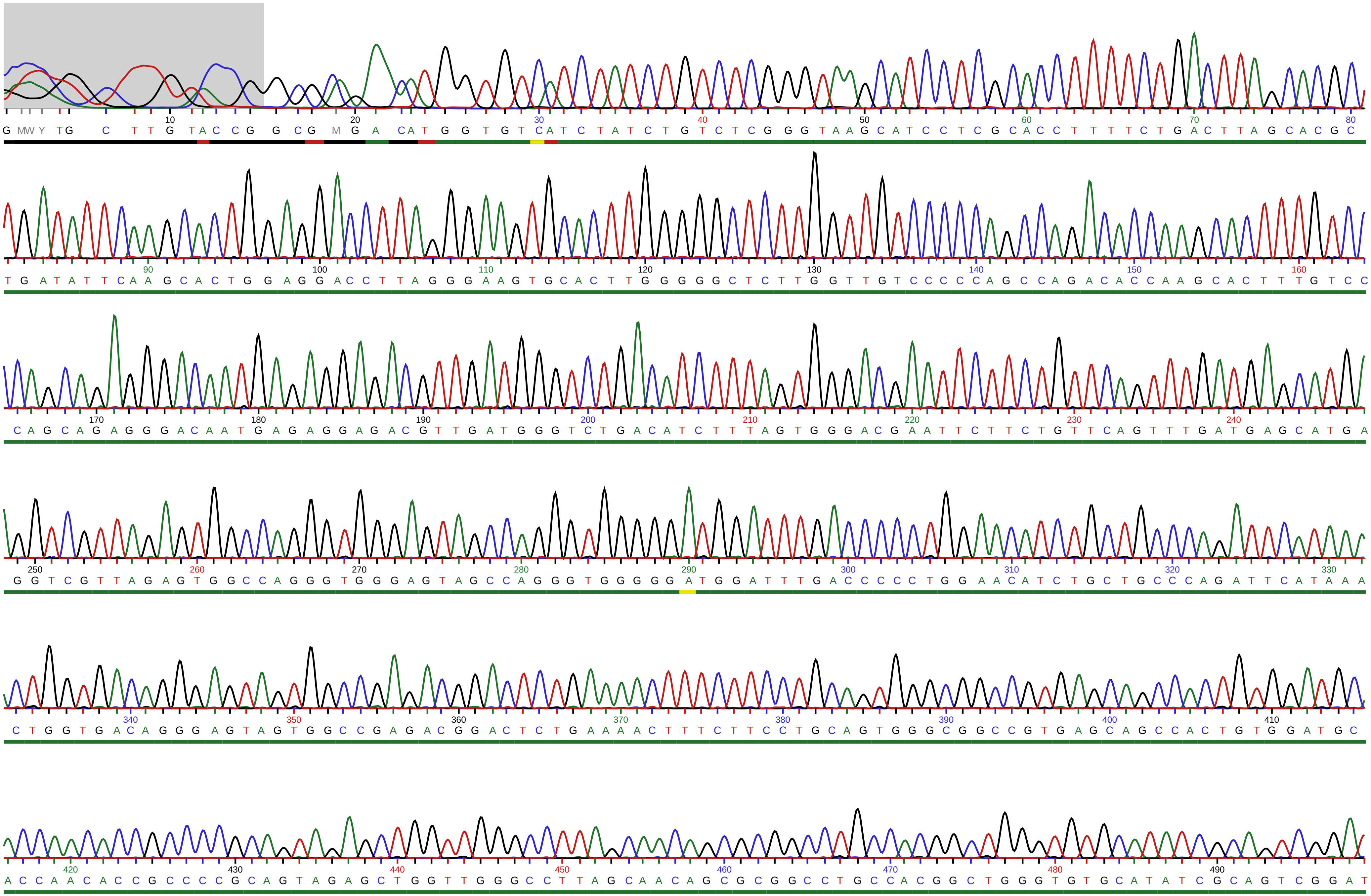

Clip. 1 BQ 20 WL 10 Sequence: KLK5 V5 Reverse

Clipped length:	621	Samples:	12961	Quality: 0 - 9	
Left clip:	16	Bases:	653	10 - 19	
Right clip:	636	Average spacing:	20.0	20 - 29	
Avg. qual. in clip.:	51.87	Average quality >=	10: 10, 20: 11, 30: 598	>= 30	

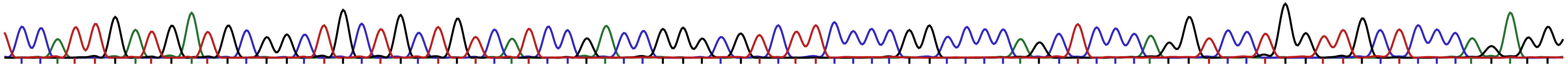

500 510 520 530 540 550 560 570 580

C C A T T G A T G A T G C G G C T G C T G C T G T C A T C C G A C C G G G C G T C T T C C C C G G C C C C A G C T C C C A G G T C C T G G T T G C T C C C A G A G G

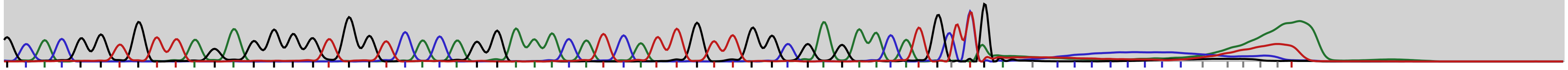

590 600 610 620 630 640 650

G C A C G G T G T T A G A G G G G T G G T C A C A G G A A A C A T C A T T G T T G G C G A G A A C A T G Y T G A W C C C C C C C C Y W W W W T

Clip. 1 BQ 20 WL 10 Sequence: KLK5 V6 Forward

Clipped length:	630	Samples:	8072	Quality: 0 - 9	
Left clip:	10	Bases:	675	10 - 19	
Right clip:	639	Average spacing:	12.0	20 - 29	
Avg. qual. in clip.:	52.12	Average quality >=	10: 25, 20: 15, 30: 601	>= 30	

22.09.2016

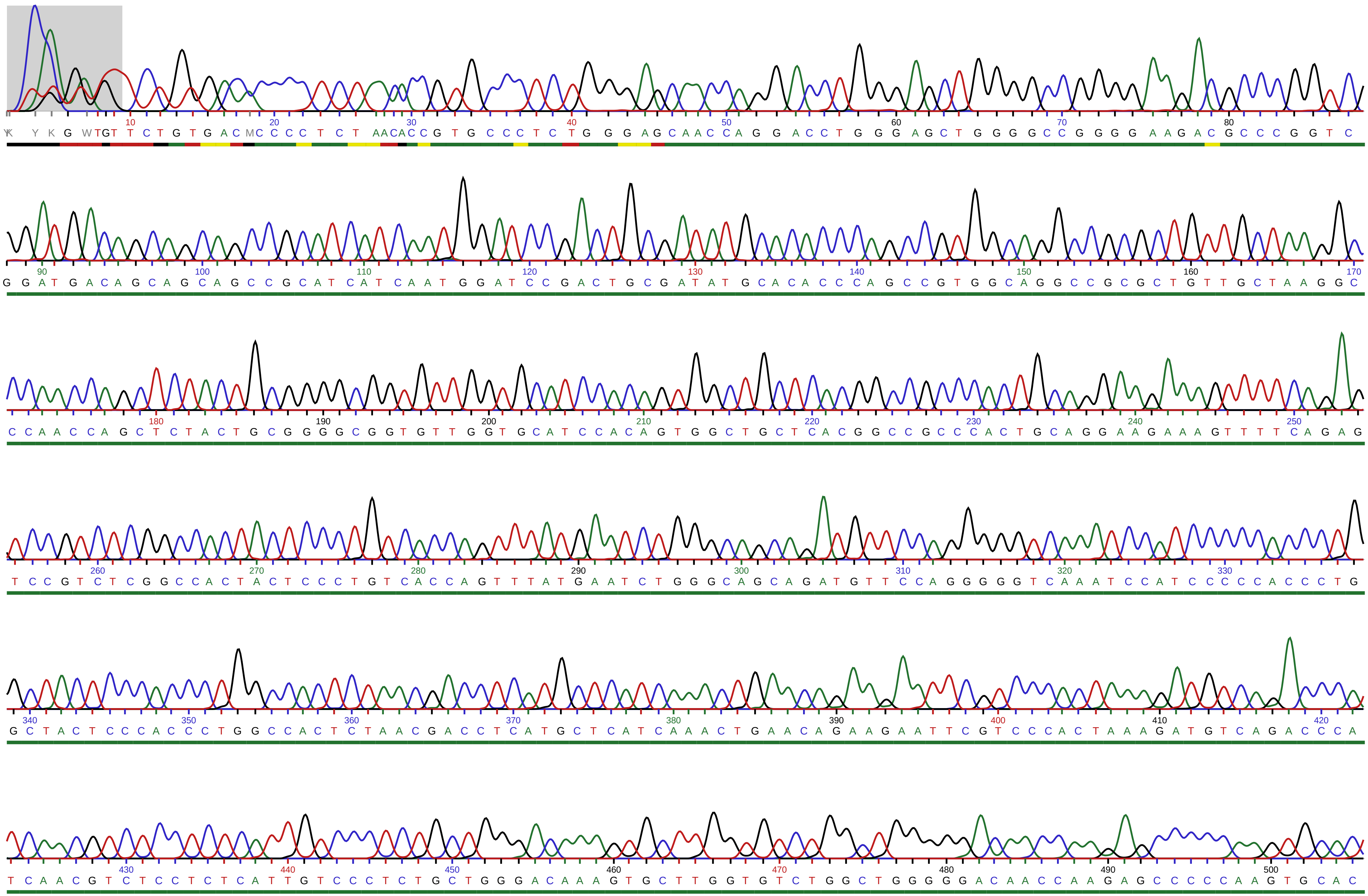

Clip. 1 BQ 20 WL 10 Sequence: KLK5 V6 Forward

Clipped length:	630	Samples:	8072	Quality: 0 - 9	
Left clip:	10	Bases:	675	10 - 19	
Right clip:	639	Average spacing:	12.0	20 - 29	
Avg. qual. in clip.:	52.12	Average quality >=	10: 25, 20: 15, 30: 601	>= 30	

22.09.2016

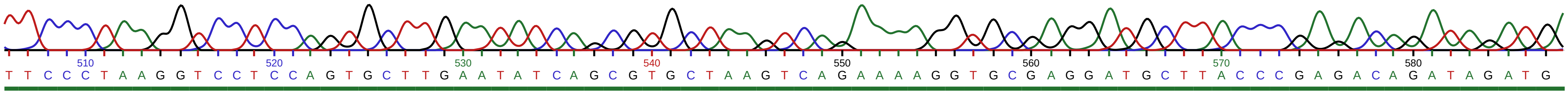

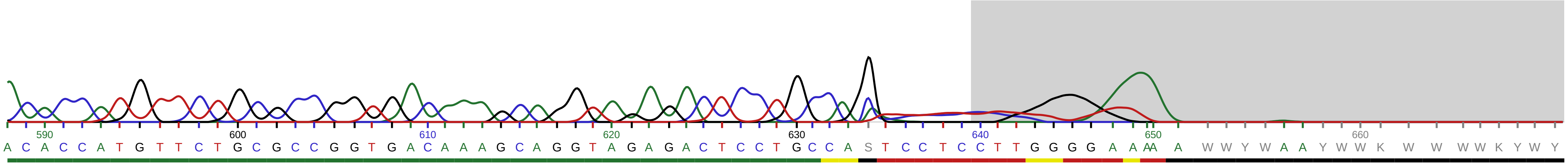

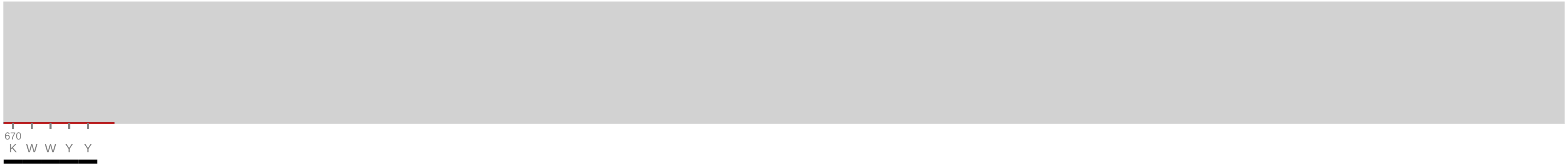

Clip. 1 BQ 20 WL 10 Sequence: KLK5 V6 Reverse

Clipped length: 620
Left clip: 18
Right clip: 637
Avg. qual. in clip.: 51.0

Samples: 12962
Bases: 659
Average spacing: 20.0
Average quality >= 10: 6, 20: 18, 30: 593

Quality: 0 - 9; 10 - 19; 20 - 29; >= 30

26.05.2017

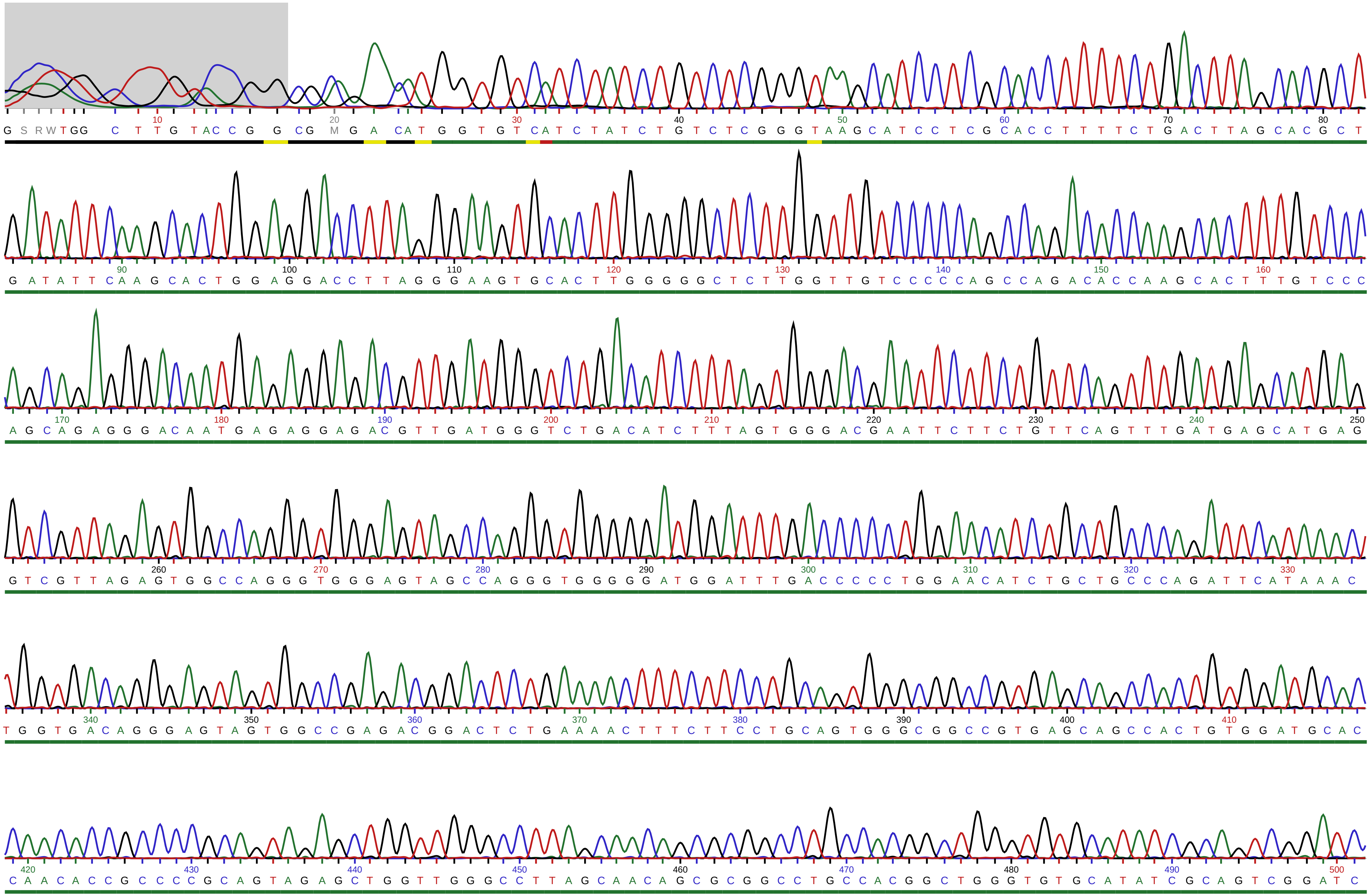

Clip. 1 BQ 20 WL 10 Sequence: KLK5 V6 Reverse

Clipped length:	620	Samples:	12962	Quality:	0 - 9	
Left clip:	18	Bases:	659		10 - 19	
Right clip:	637	Average spacing:	20.0		20 - 29	
Avg. qual. in clip.:	51.0	Average quality >=	10: 6, 20: 18, 30: 593		>= 30	

26.05.2017

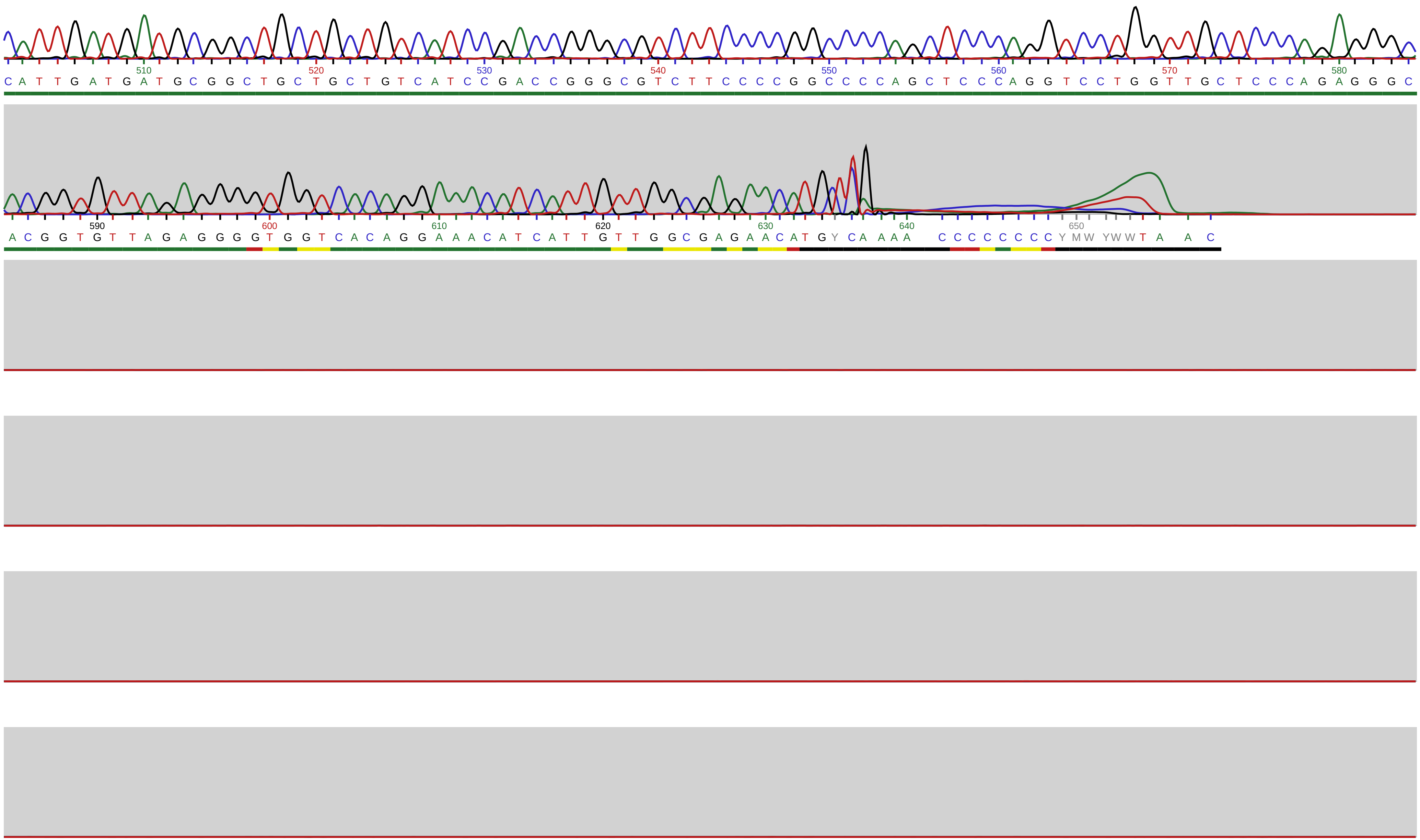

Clip. 1 BQ 20 WL 10

Sequence: KLK6 V7 Forward

Clipped length: 388
Left clip: 12
Right clip: 399
Avg. qual. in clip.: 50.19

Samples: 12962
Bases: 399
Average spacing: 33.0
Average quality >= 10: 7, 20: 18, 30: 360

Quality: 0 - 9, 10 - 19, 20 - 29, >= 30

22.09.2016

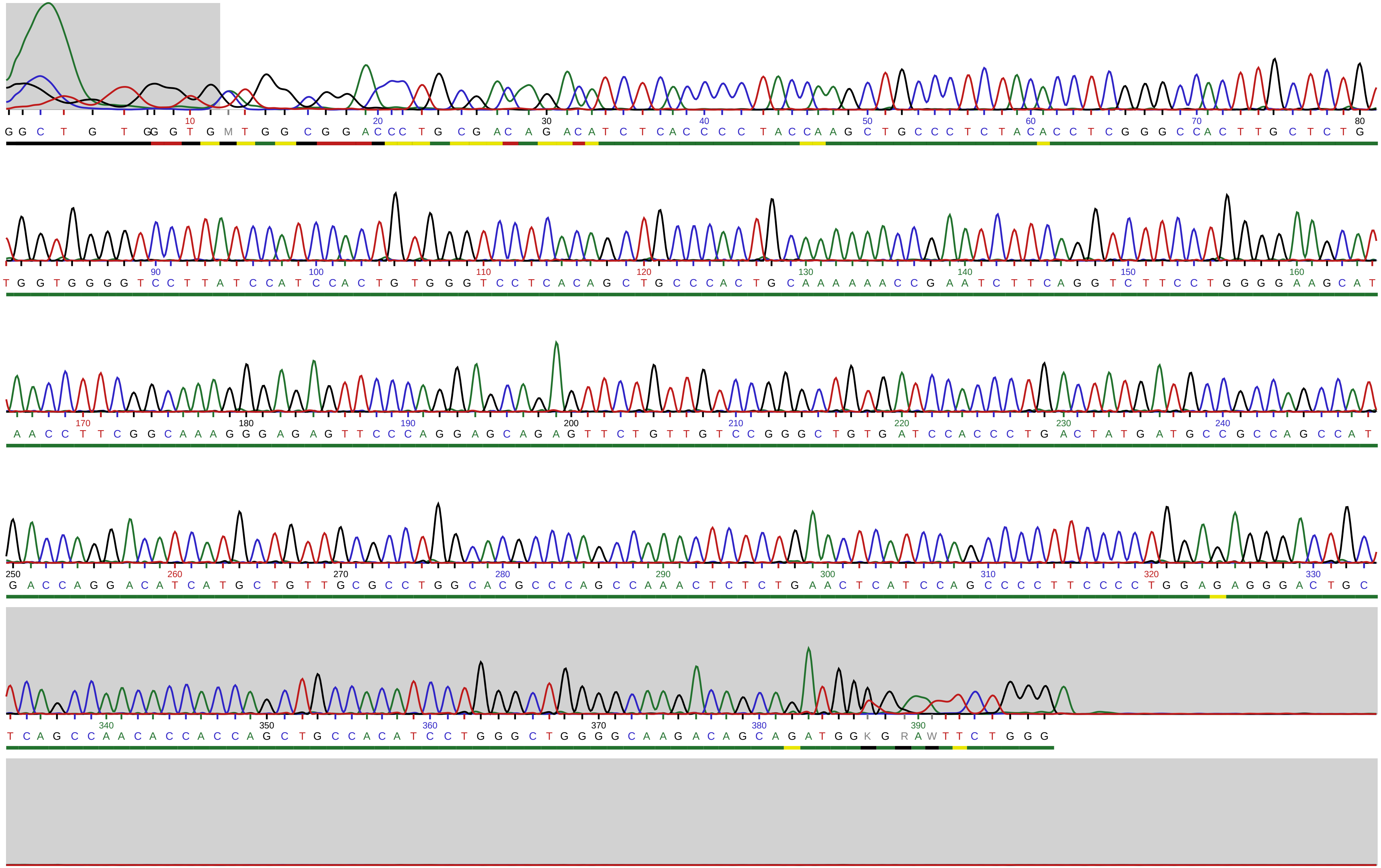

Clip. 1 BQ 20 WL 10 Sequence: KLK6 V7 Reverse

Clipped length: 383
Left clip: 9
Right clip: 391
Avg. qual. in clip.: 50.21

Samples: 12961
Bases: 652
Average spacing: 20.0
Average quality >= 10: 167, 20: 47, 30: 360

Quality: 0 - 9, 10 - 19, 20 - 29, >= 30

26.05.2017

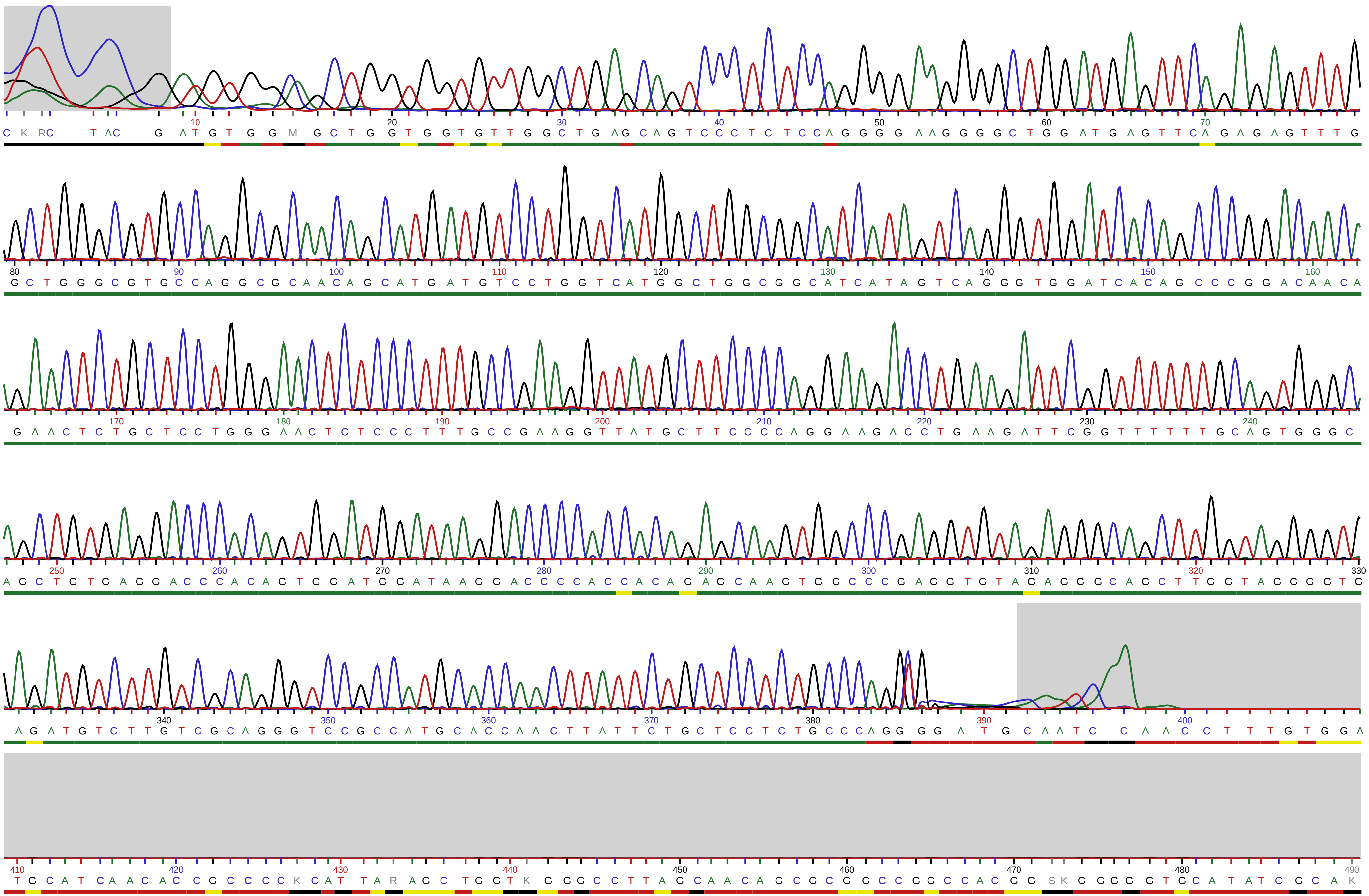

Clip. 1 BQ 20 WL 10 Sequence: KLK6 V8 Forward

Clipped length: 230
Left clip: 14
Right clip: 243
Avg. qual. in clip.: 48.7

Samples: 12962
Bases: 396
Average spacing: 33.0
Average quality >= 10: 115, 20: 30, 30: 205

Quality: 0 - 9
10 - 19
20 - 29
>= 30

22.09.2016

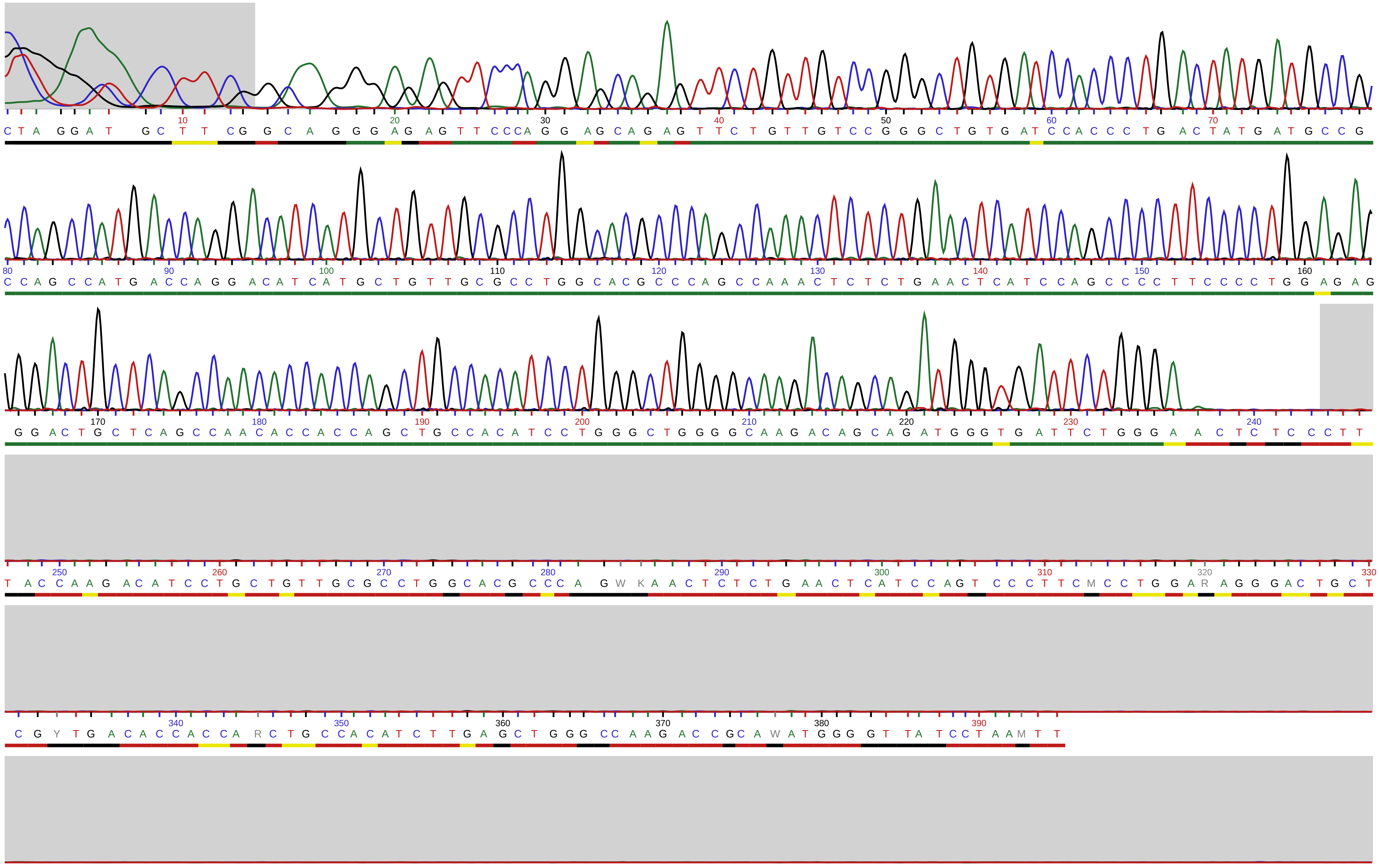

Clip. 1 BQ 20 WL 10 Sequence: KLK6 V8 Reverse

Clipped length: 223
Left clip: 9
Right clip: 231
Avg. qual. in clip.: 49.27

Samples: 12962
Bases: 388
Average spacing: 34.0
Average quality >= 10: 108, 20: 39, 30: 202

Quality: 0 - 9; 10 - 19; 20 - 29; >= 30

26.05.2017

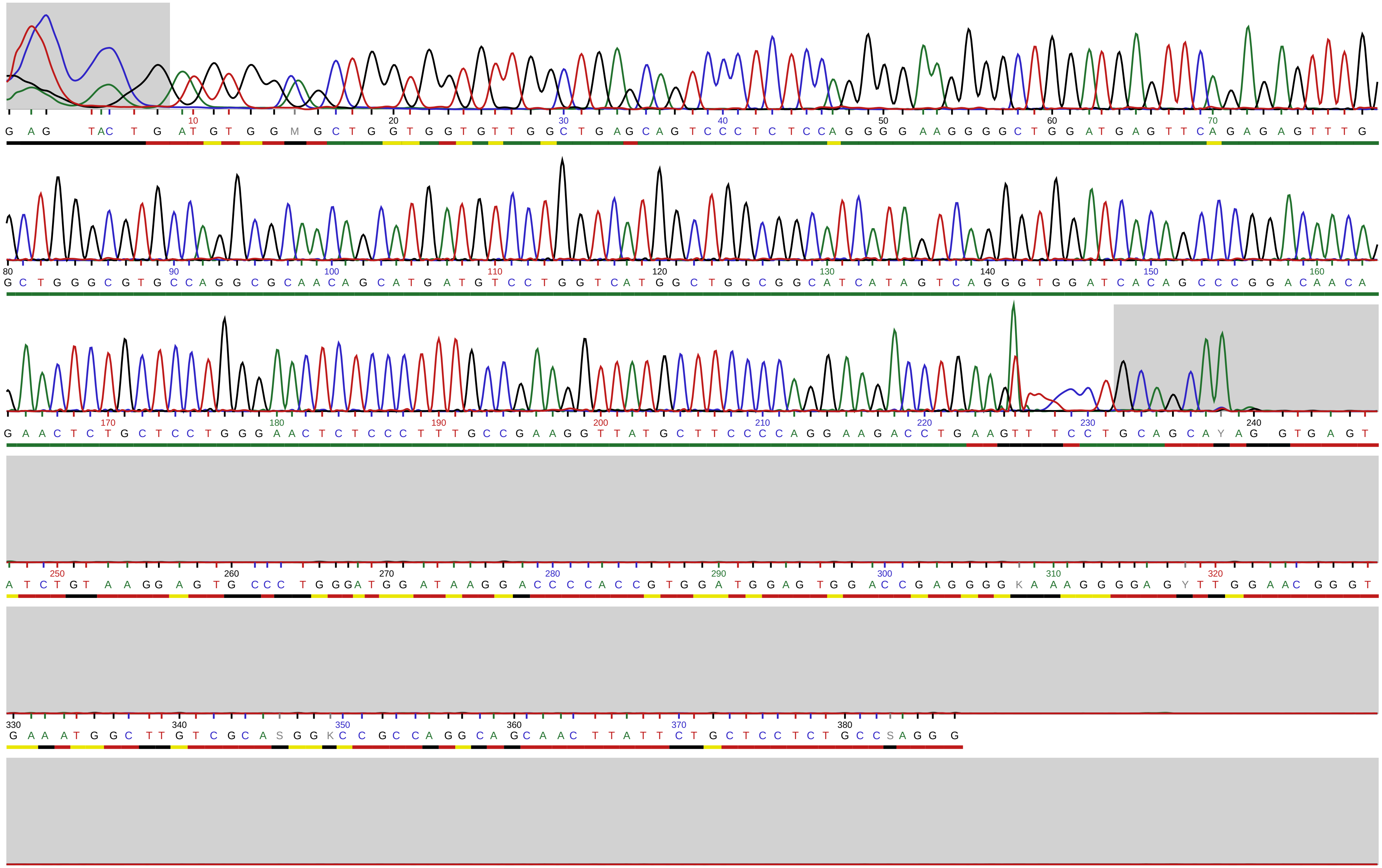

Clip. 1 BQ 20 WL 10 Sequence: KLK6 V10 Forward

Clipped length:	378	Samples:	12963	Quality: 0 - 9	
Left clip:	14	Bases:	598	10 - 19	
Right clip:	391	Average spacing:	22.0	20 - 29	
Avg. qual. in clip.:	49.55	Average quality >=	10: 118, 20: 35, 30: 342	>= 30	

22.09.2016

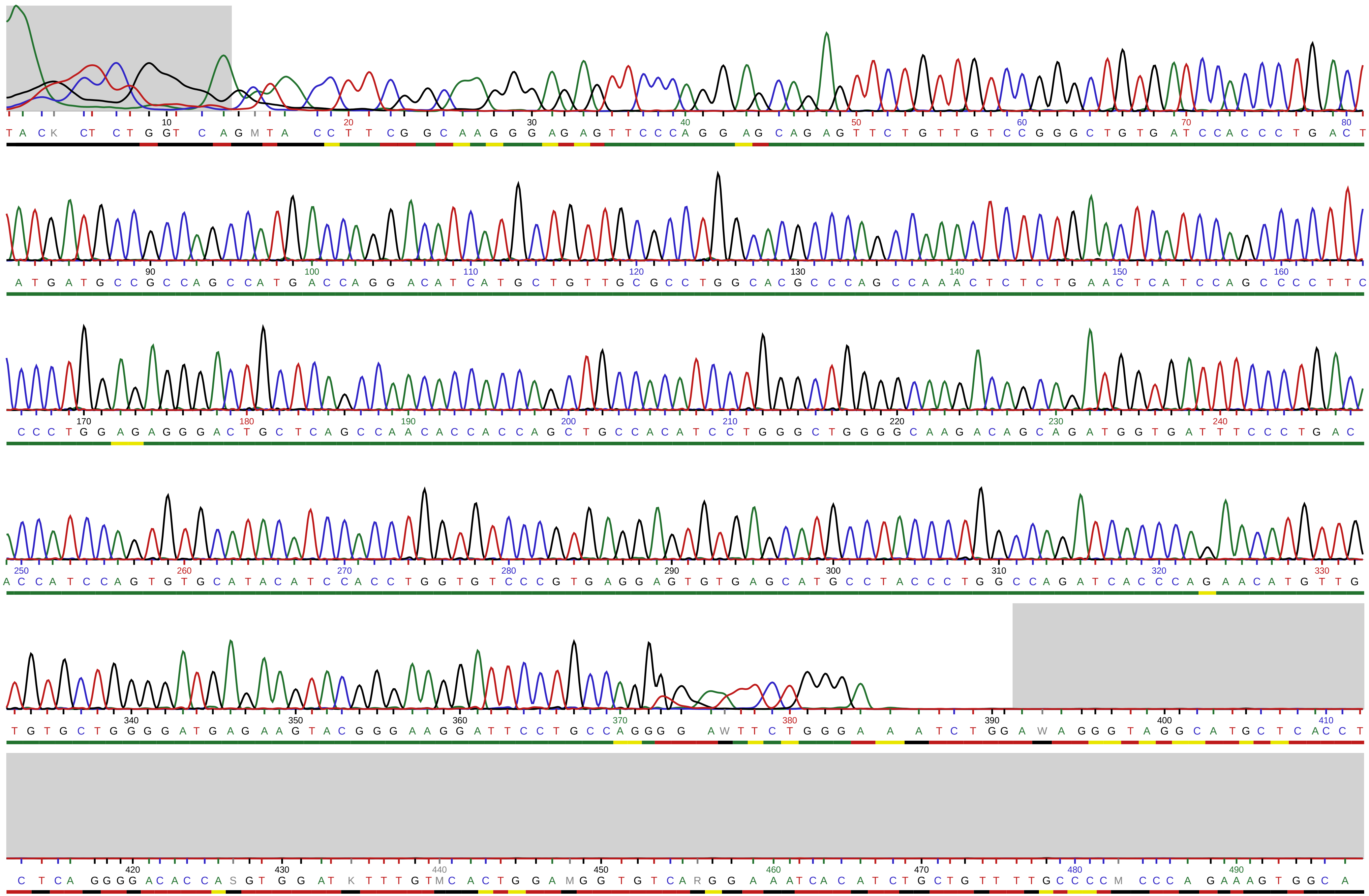

Clip. 1 BQ 20 WL 10 Sequence: KLK6 V10 Reverse

Clipped length: 359
Left clip: 19
Right clip: 377
Avg. qual. in clip.: 51.4

Samples: 12963
Bases: 385
Average spacing: 34.0
Average quality >= 10: 11, 20: 8, 30: 340

Quality: 0 - 9; 10 - 19; 20 - 29; >= 30

Page: 1 / 3
26.05.2017

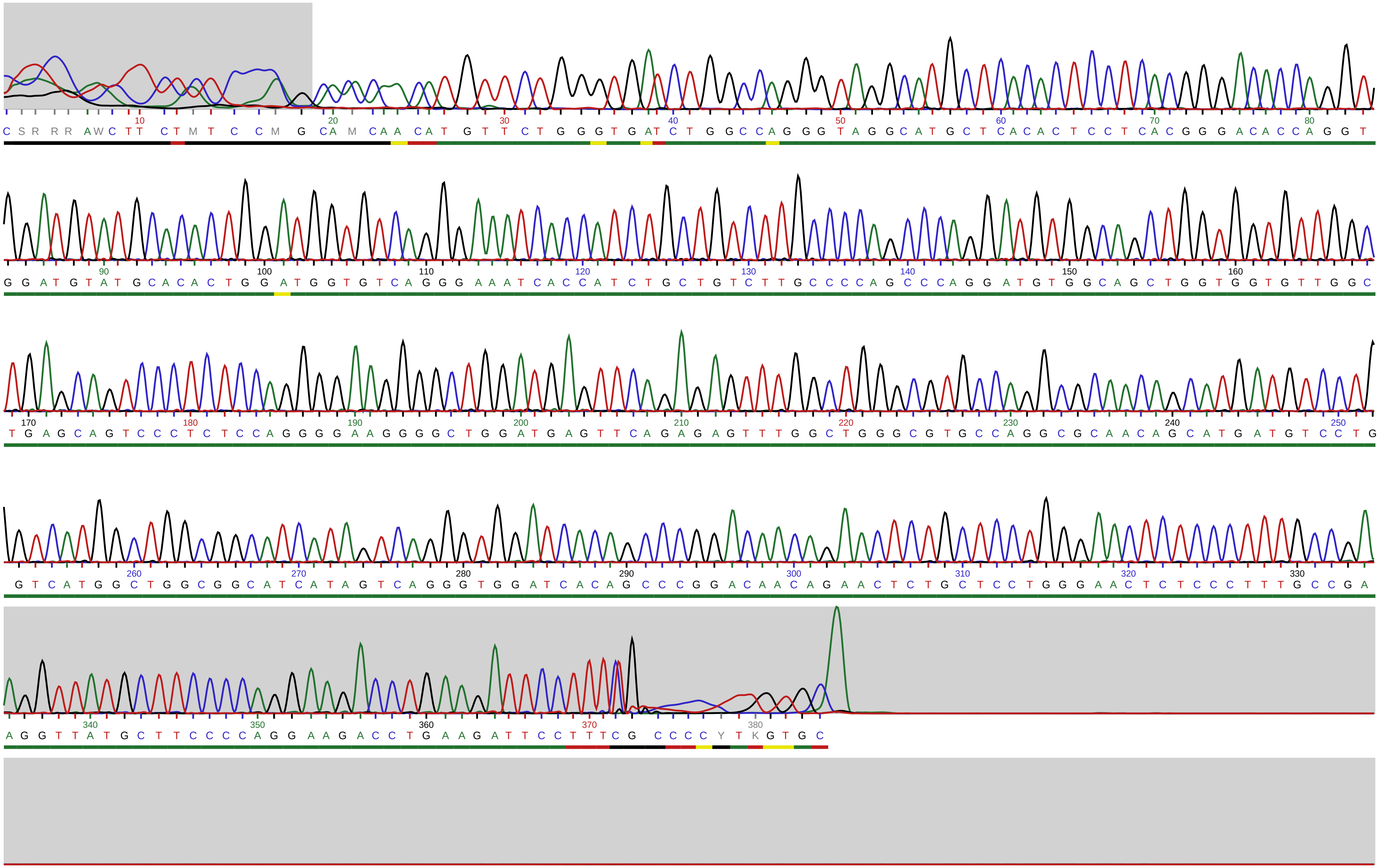

Clip. 1 BQ 20 WL 10 Sequence: KLK6 V11 Forward

Clipped length:	242	Samples:	12962	Quality:	0 - 9	
Left clip:	13	Bases:	431		10 - 19	
Right clip:	254	Average spacing:	31.0		20 - 29	
Avg. qual. in clip.:	48.33	Average quality >=	10: 139, 20: 20, 30: 212		>= 30	

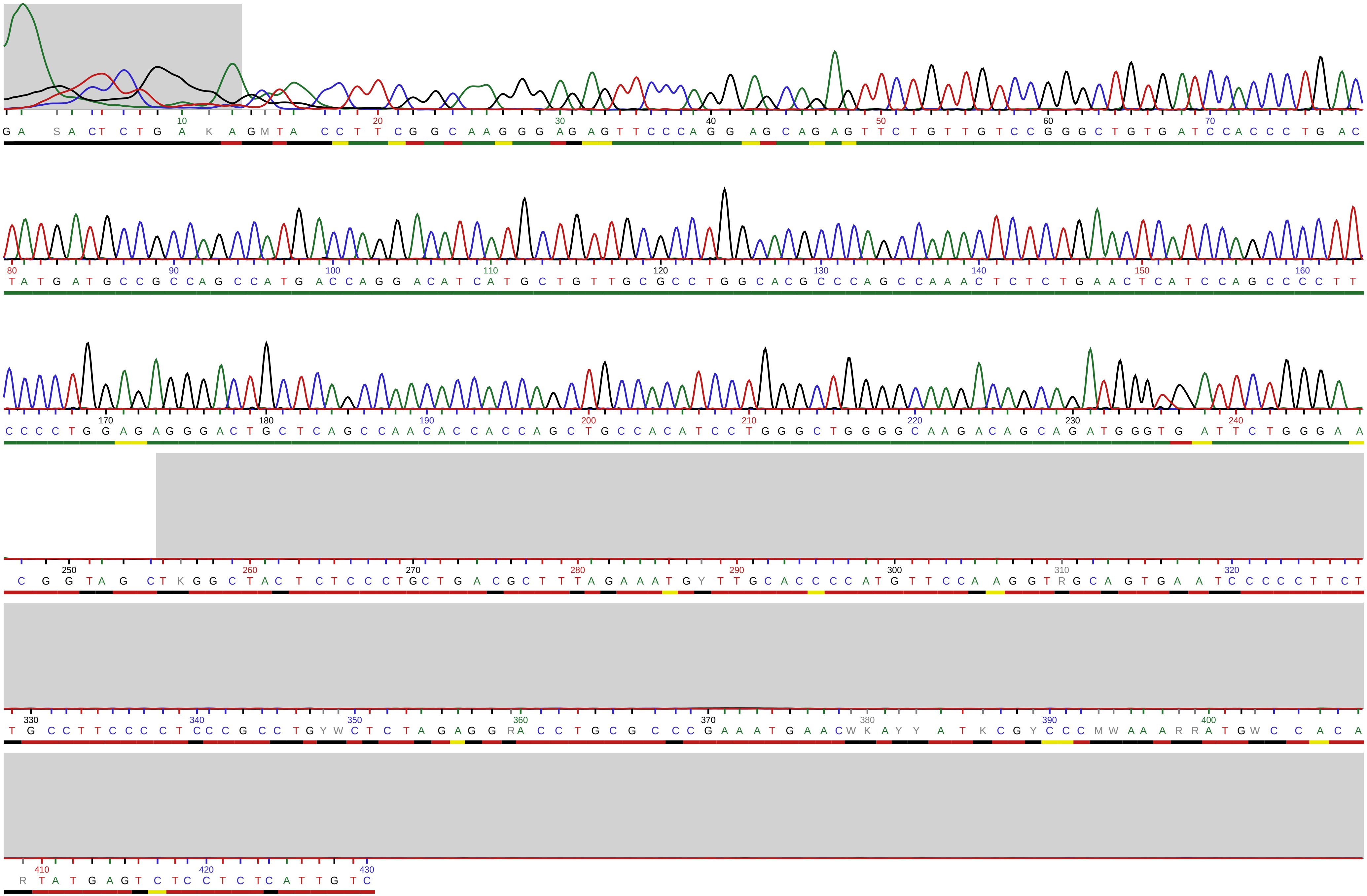

Clip. 1 BQ 20 WL 10 Sequence: KLK6 V11 Reverse

Clipped length: 241
Left clip: 12
Right clip: 252
Avg. qual. in clip.: 47.82

Samples: 12963
Bases: 431
Average spacing: 31.0
Average quality >= 10: 151, 20: 22, 30: 208

Quality: 0 - 9
10 - 19
20 - 29
>= 30

26.05.2017

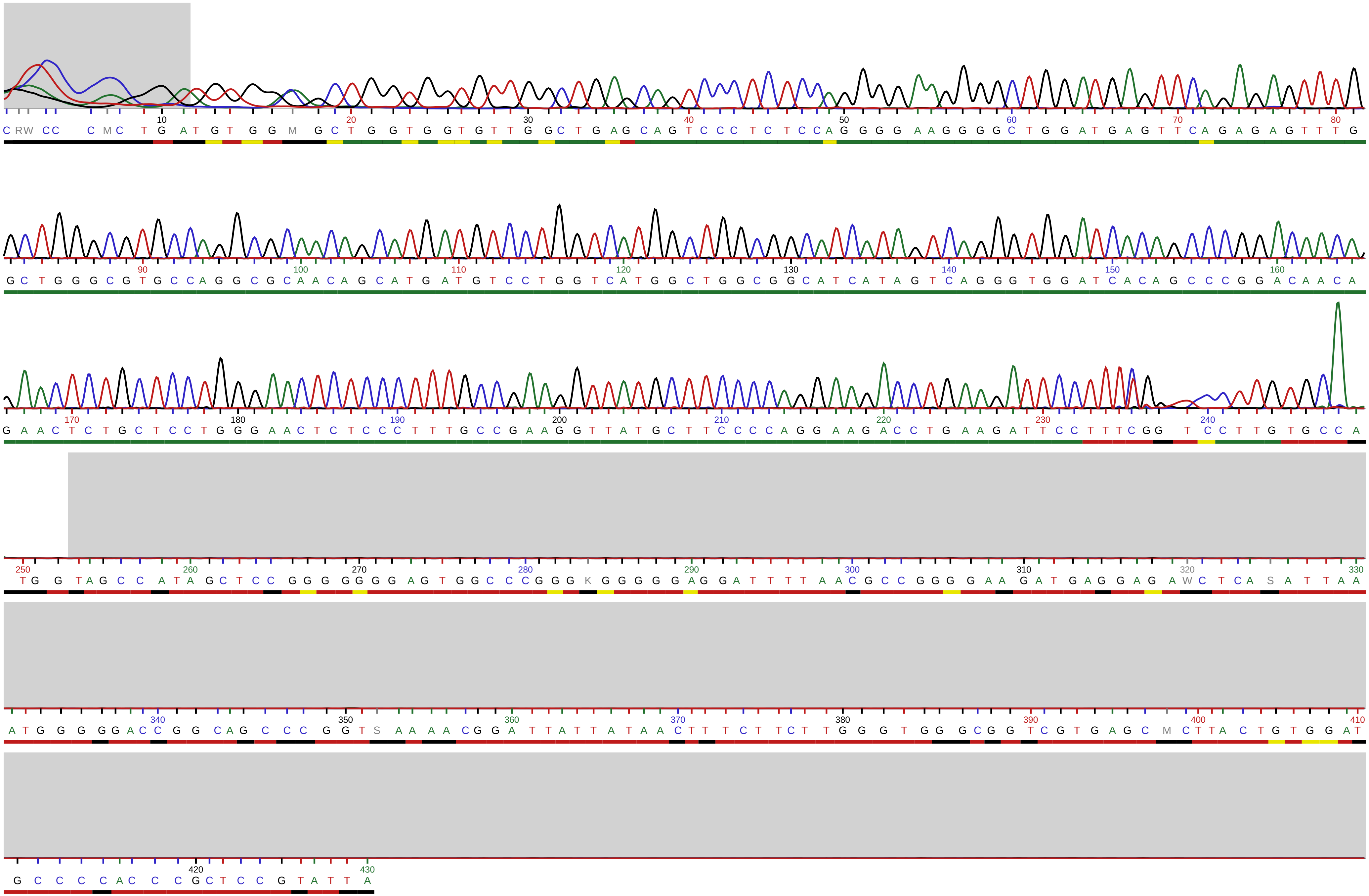

Clip. 1 BQ 20 WL 10 Sequence: KLK8 V12 Forward

Clipped length:	242	Samples:	12964	Quality:	0 - 9	Page: 1 / 3
Left clip:	22	Bases:	444		10 - 19	26.05.2017
Right clip:	263	Average spacing:	30.0		20 - 29	
Avg. qual. in clip.:	47.27	Average quality >=	10: 143, 20: 35, 30: 210		>= 30	

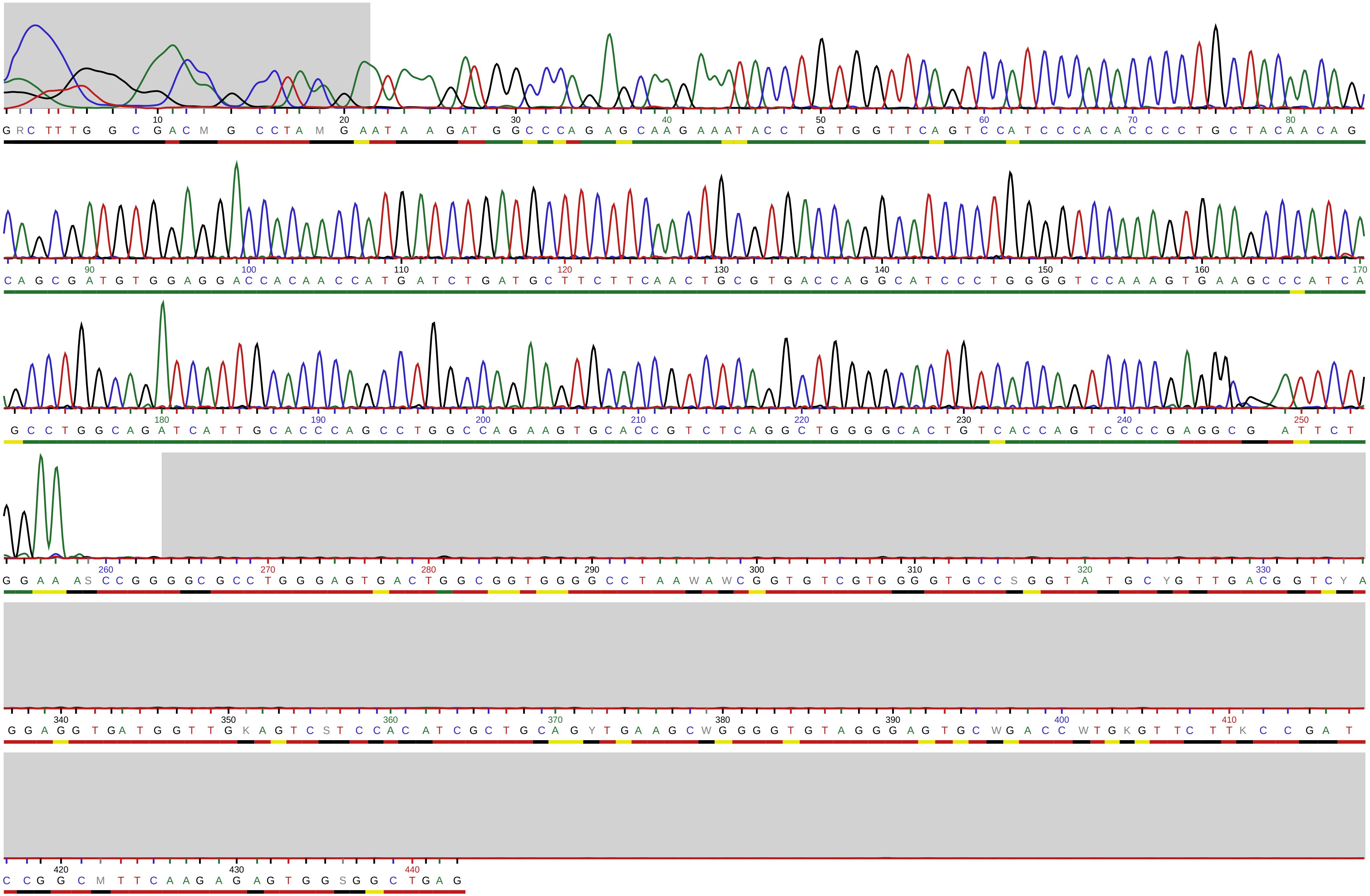

Clip. 1 BQ 20 WL 10 Sequence: KLK8 V12 Reverse

Clipped length: 252
Left clip: 11
Right clip: 262
Avg. qual. in clip.: 47.8

Samples: 12966
Bases: 510
Average spacing: 26.0
Average quality >= 10: 194, 20: 17, 30: 219

Quality: 0 - 9
10 - 19
20 - 29
>= 30

22.09.2016

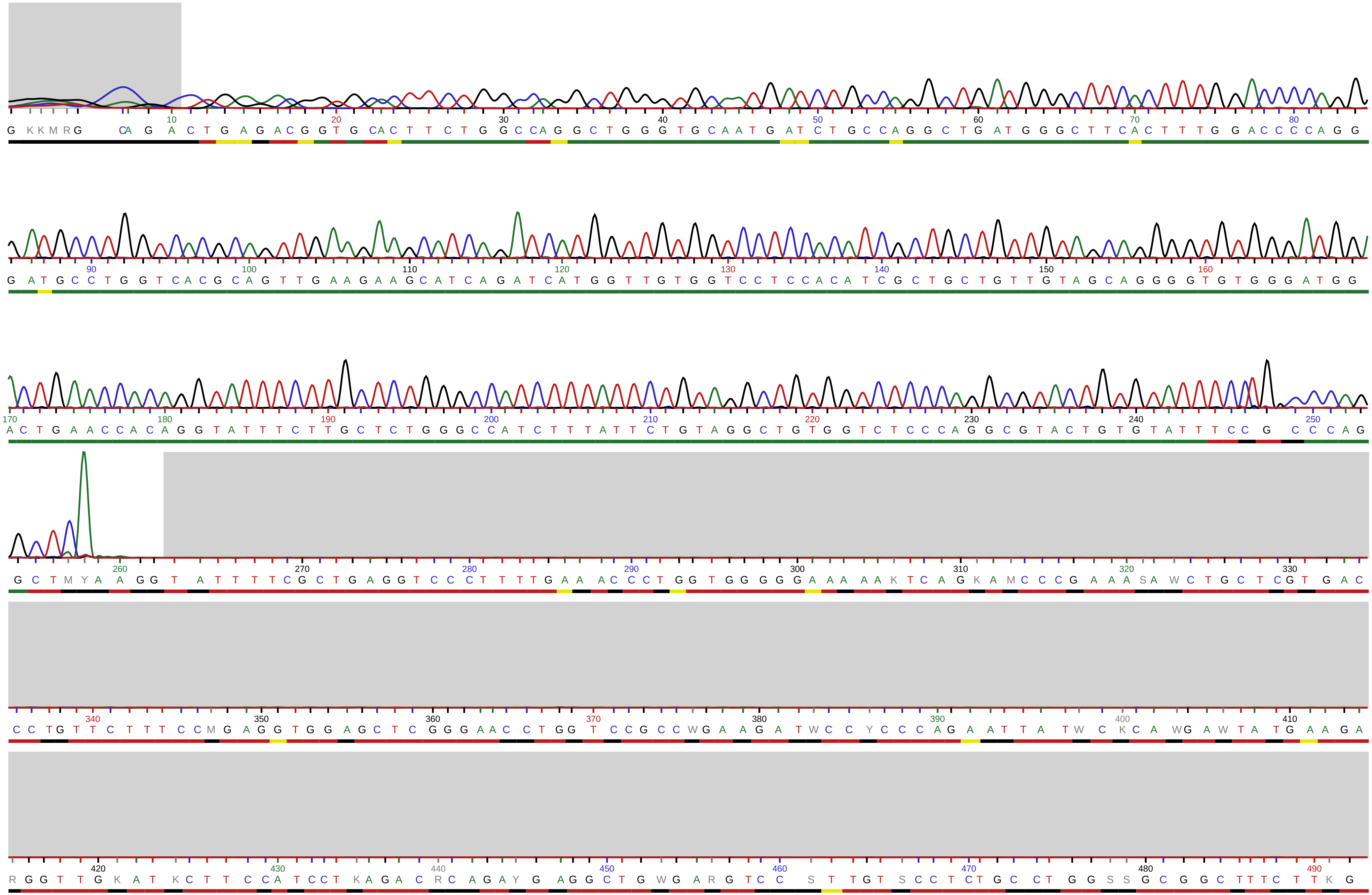

Clip. 1 BQ 20 WL 10 Sequence: KLK8 V13 Forward

Clipped length:	403	Samples:	12965	Quality:	0 - 9
Left clip:	9	Bases:	692		10 - 19
Right clip:	411	Average spacing:	19.0		20 - 29
Avg. qual. in clip.:	50.46	Average quality >=	10: 160, 20: 18, 30: 376		>= 30

03.06.2017

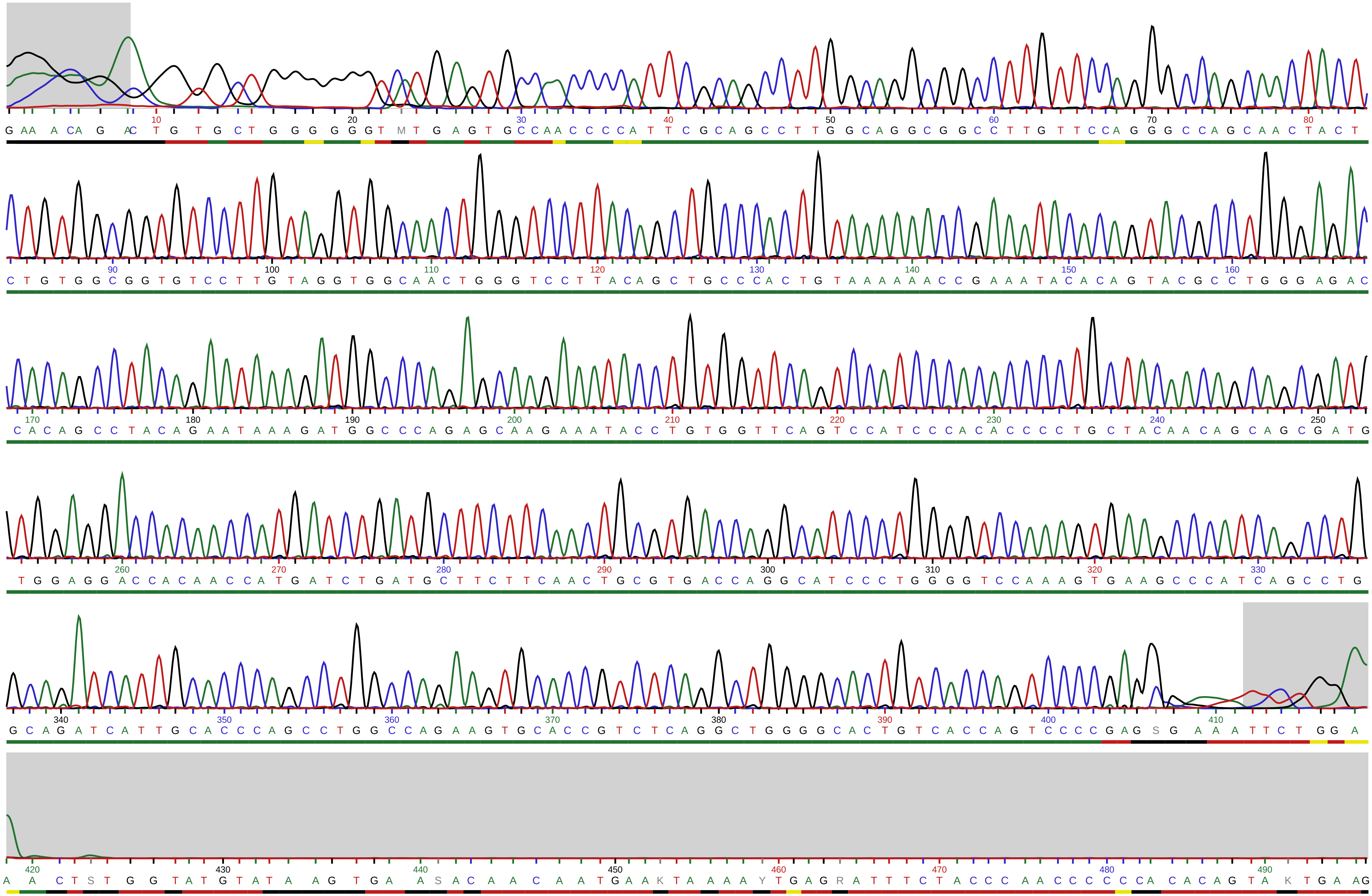

Clip. 1 BQ 20 WL 10 Sequence: KLK8 V13 Reverse

Clipped length:	409	Samples:	12962	Quality:	0 - 9		Page: 1 / 3
Left clip:	13	Bases:	553		10 - 19		22.09.2016
Right clip:	421	Average spacing:	24.0		20 - 29		
Avg. qual. in clip.:	49.89	Average quality >=	10: 78, 20: 17, 30: 374		>= 30		

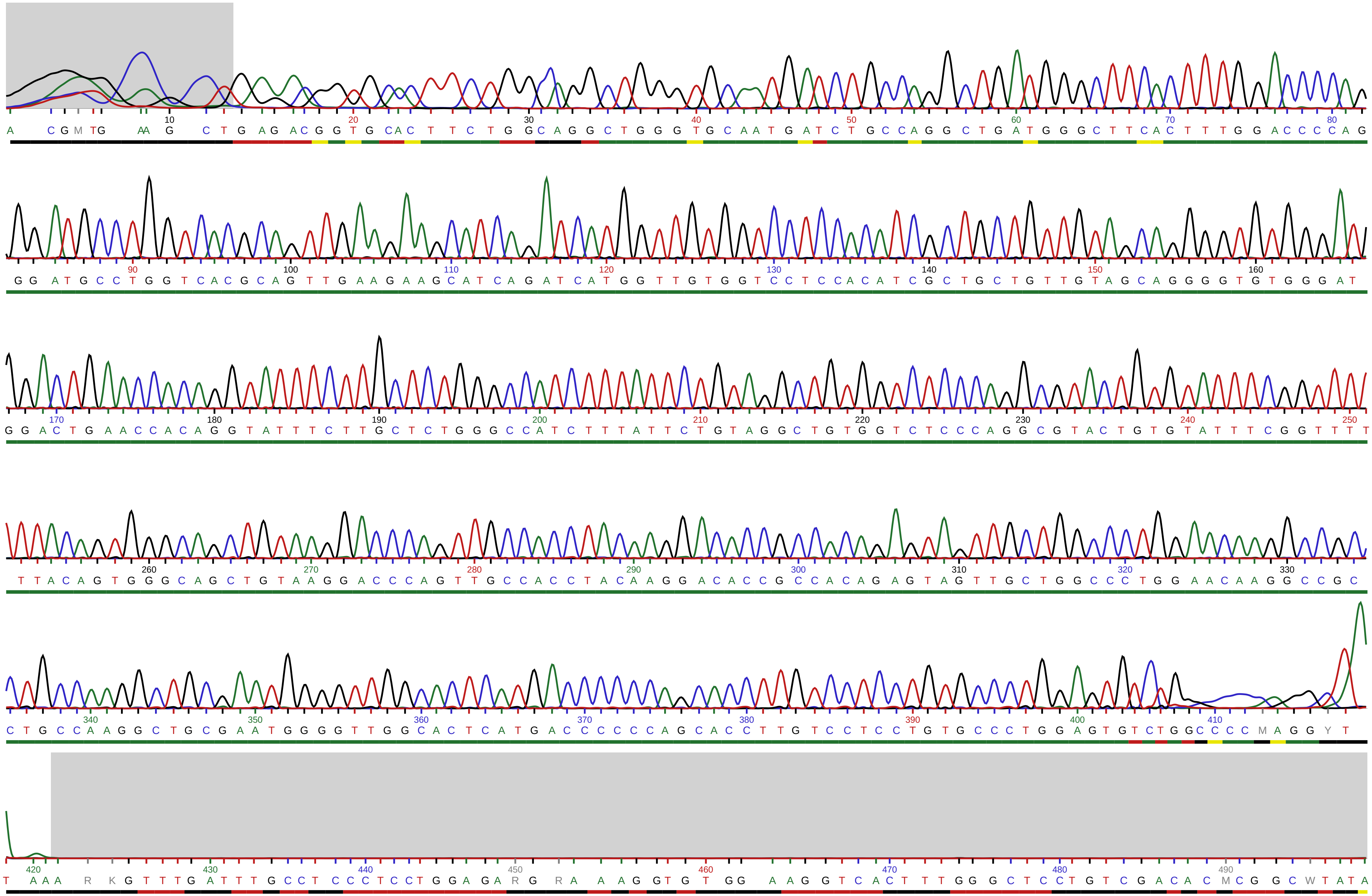

Clip. 1 BQ 20 WL 10 Sequence: KLK8 V14 Forward

Clipped length:	540	Samples:	12964	Quality:	0 - 9	Page: 1 / 3
Left clip:	23	Bases:	1019		10 - 19	03.06.2017
Right clip:	562	Average spacing:	13.0		20 - 29	
Avg. qual. in clip.:	46.47	Average quality >=	10: 200, 20: 64, 30: 499		>= 30	

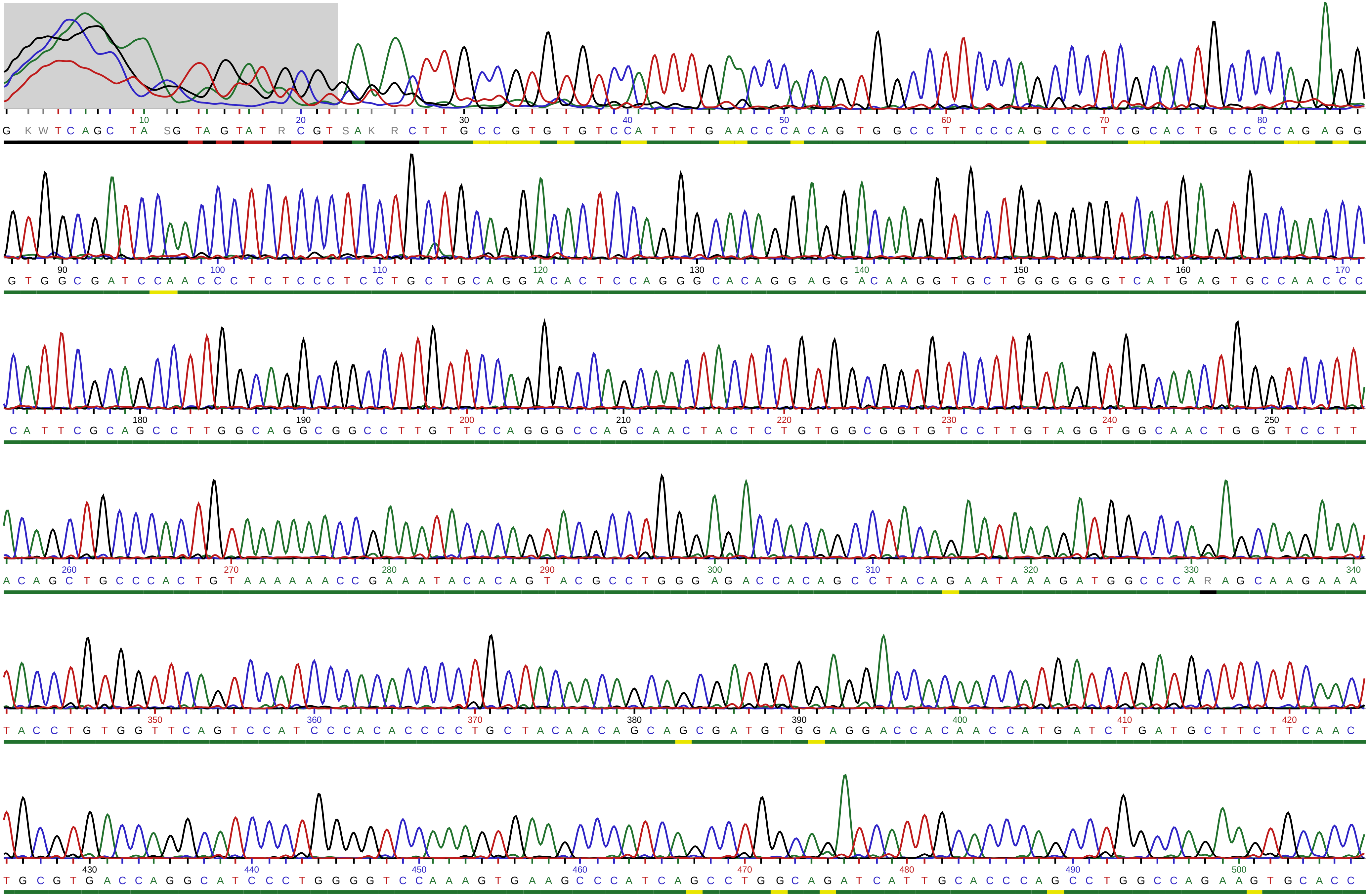

Clip. 1 BQ 20 WL 10 Sequence: KLK8 V14 Forward

Clipped length:	540	Samples:	12964	Quality: 0 - 9	
Left clip:	23	Bases:	1019	10 - 19	
Right clip:	562	Average spacing:	13.0	20 - 29	
Avg. qual. in clip.:	46.47	Average quality >=	10: 200, 20: 64, 30: 499	>= 30	

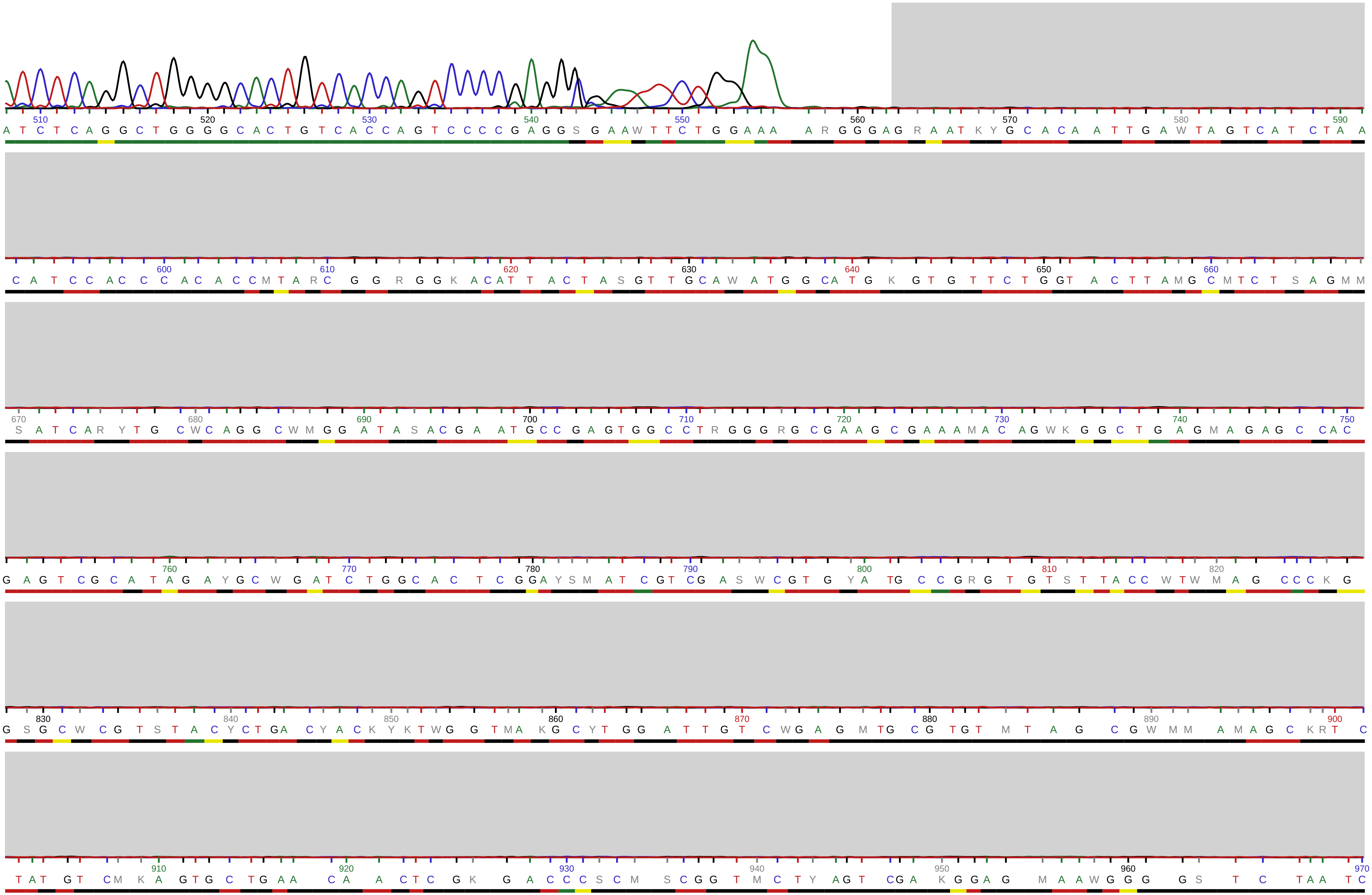

Clip. 1 BQ 20 WL 10 Sequence: KLK8 V14 Reverse

Clipped length: 513
Left clip: 31
Right clip: 543
Avg. qual. in clip.: 47.24

Samples: 12964
Bases: 553
Average spacing: 24.0
Average quality >= 10: 13, 20: 30, 30: 477

Quality: 0 - 9, 10 - 19, 20 - 29, >= 30

22.09.2016

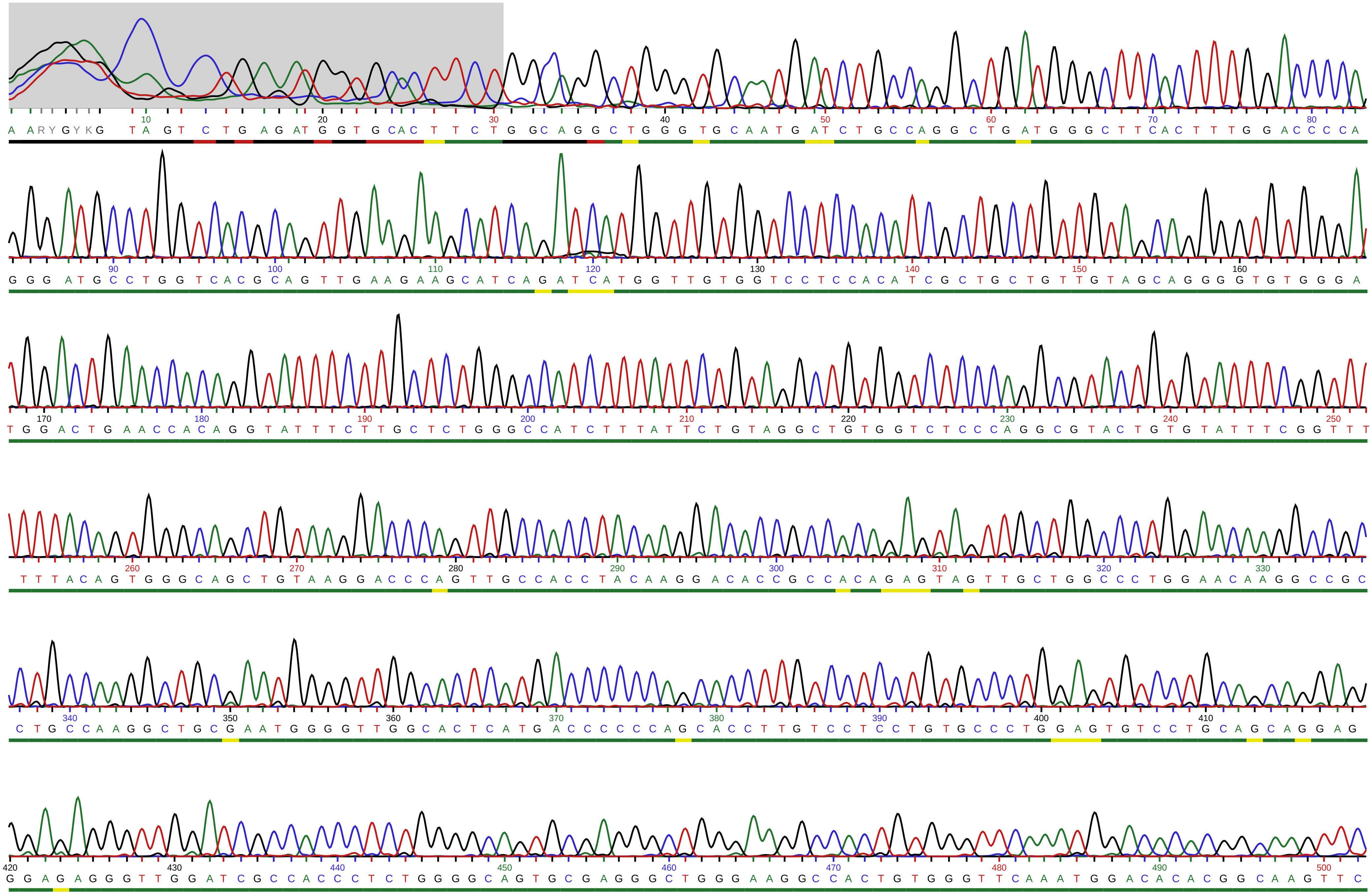

Clip. 1 BQ 20 WL 10 Sequence: KLK8 V14 Reverse

Clipped length: 513
Left clip: 31
Right clip: 543
Avg. qual. in clip.: 47.24

Samples: 12964
Bases: 553
Average spacing: 24.0
Average quality >= 10: 13, 20: 30, 30: 477

Quality:
0 - 9
10 - 19
20 - 29
>= 30

22.09.2016

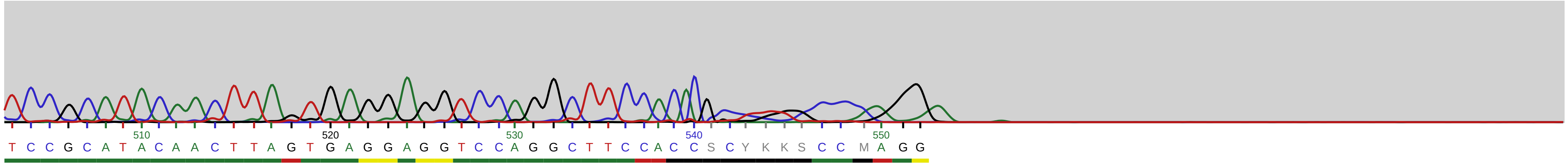

Clip. 1 BQ 20 WL 10 Sequence: KLK9 V4 Forward

Clipped length:	253	Samples:	12966	Quality: 0 - 9		Page: 1 / 3
Left clip:	12	Bases:	1010	10 - 19		03.06.2017
Right clip:	264	Average spacing:	13.0	20 - 29		
Avg. qual. in clip.:	48.73	Average quality >=	10: 396, 20: 78, 30: 227	>= 30		

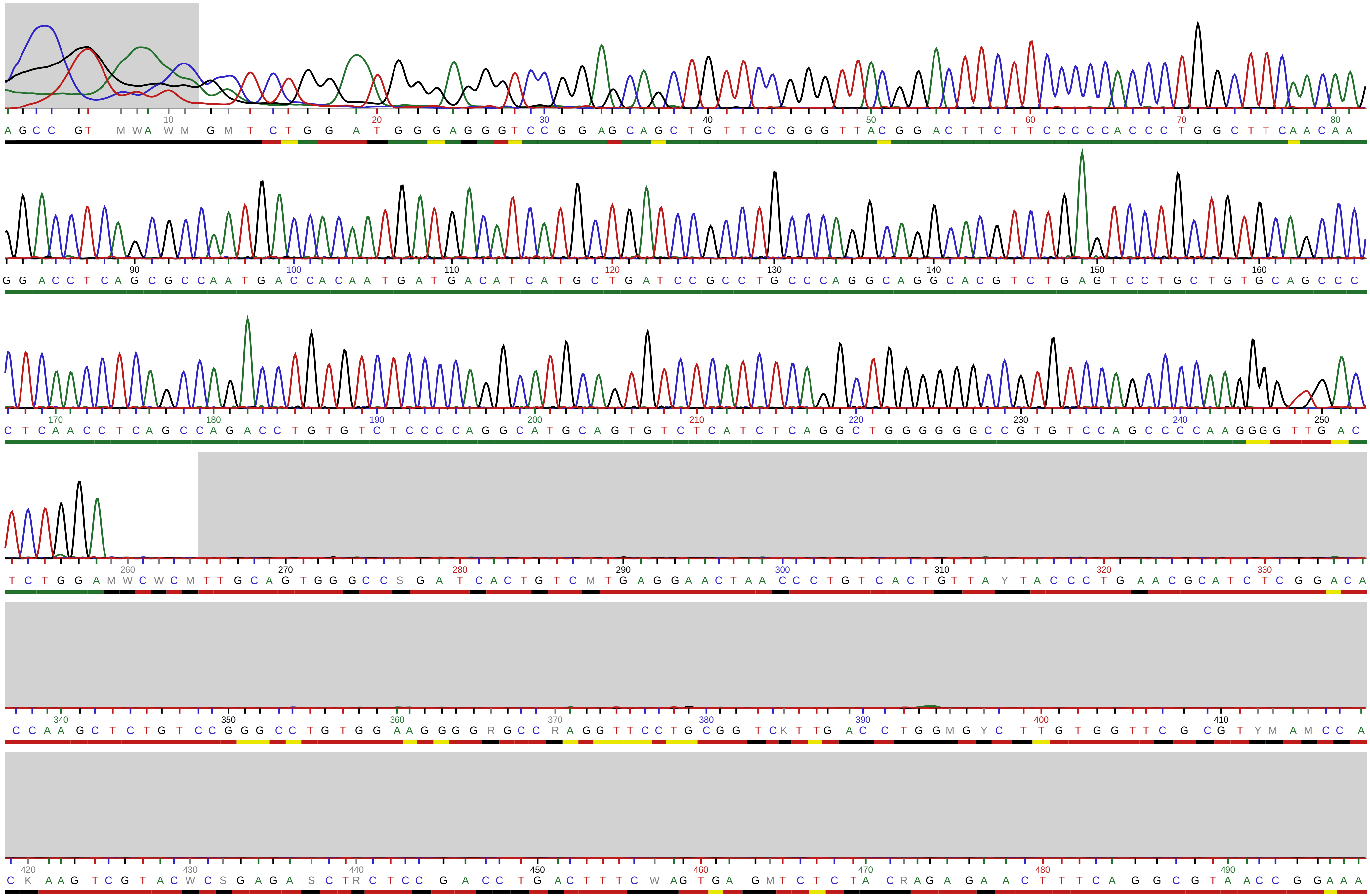

Clip. 1 BQ 20 WL 10 Sequence: KLK9 V4 Reverse

Clipped length:	248	Samples:	12961	Quality:	0 - 9	
Left clip:	25	Bases:	430		10 - 19	
Right clip:	272	Average spacing:	31.0		20 - 29	
Avg. qual. in clip.:	47.57	Average quality >=	10: 137, 20: 13, 30: 219		>= 30	

22.09.2016

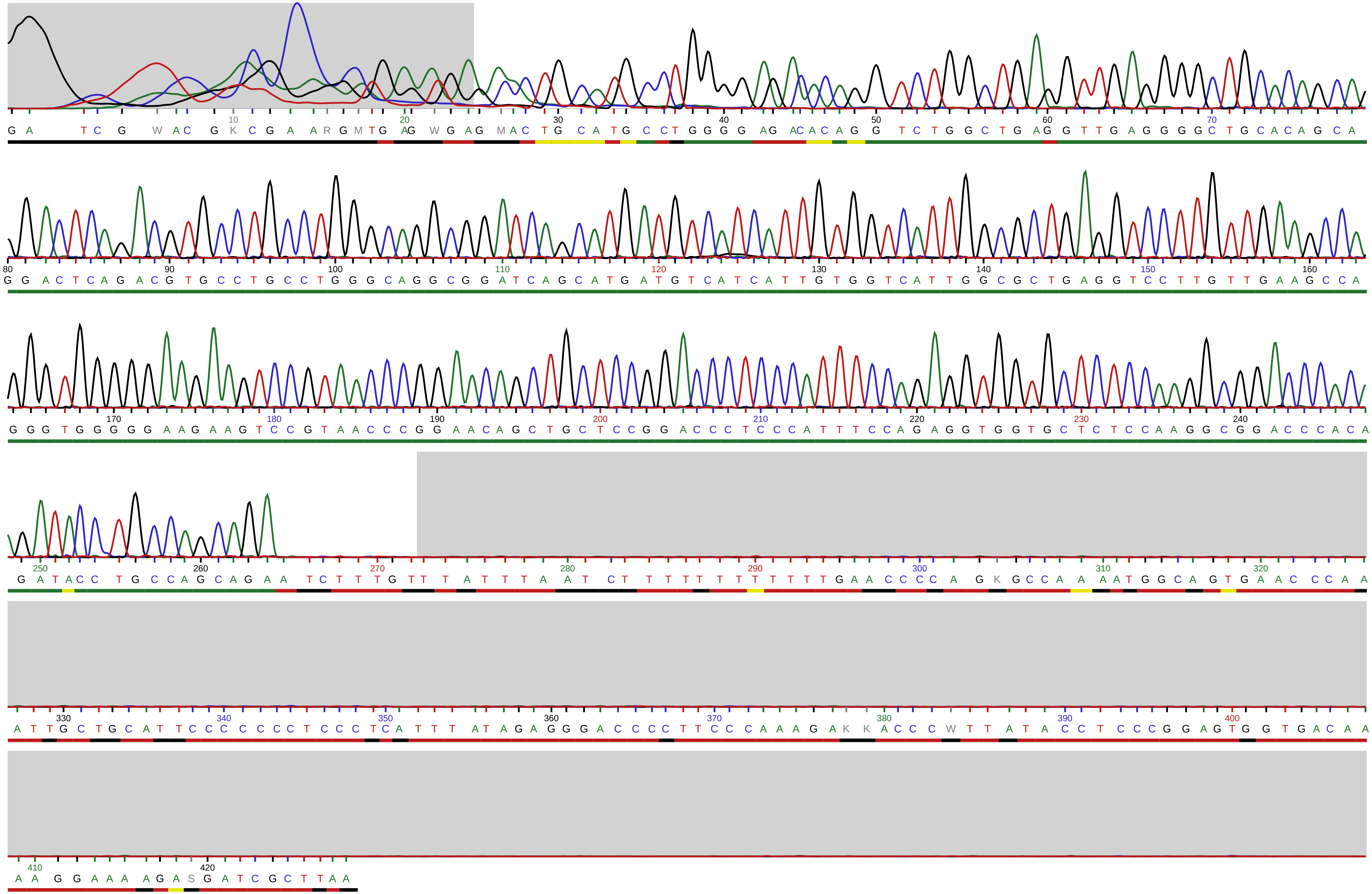

Clip. 1 BQ 20 WL 10 Sequence: KLK9 V5 Forward

Clipped length:	373	Samples:	12964	Quality: 0 - 9	
Left clip:	8	Bases:	966	10 - 19	
Right clip:	380	Average spacing:	14.0	20 - 29	
Avg. qual. in clip.:	51.62	Average quality >=	10: 334, 20: 52, 30: 347	>= 30	

03.06.2017

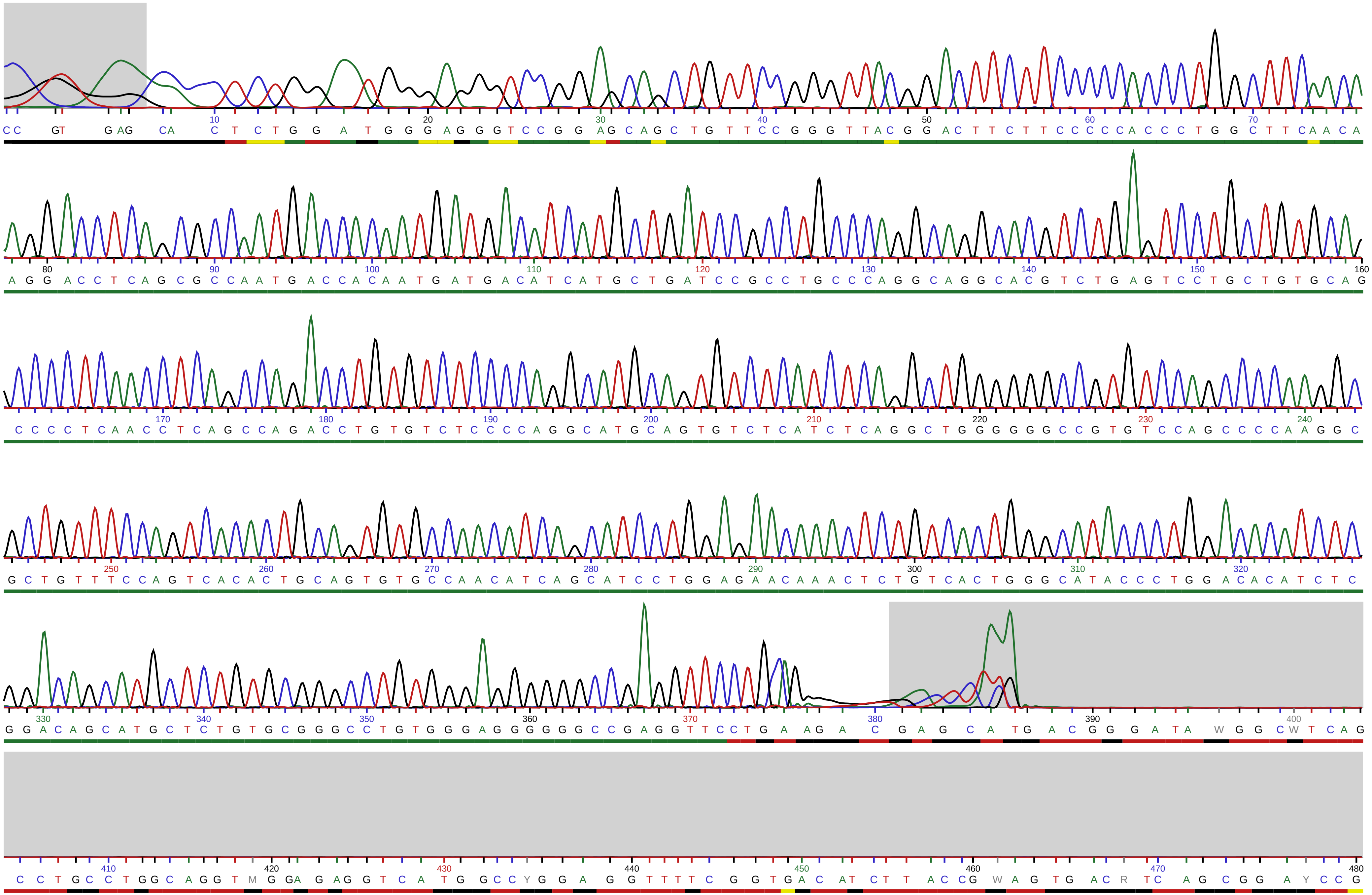

Clip. 1 BQ 20 WL 10

Sequence: KLK9 V5 Reverse

Clipped length: 369
Left clip: 14
Right clip: 382
Avg. qual. in clip.: 49.73

Samples: 12962
Bases: 517
Average spacing: 26.0
Average quality >= 10: 85, 20: 17, 30: 343

Quality: 0 - 9
10 - 19
20 - 29
>= 30

22.09.2016

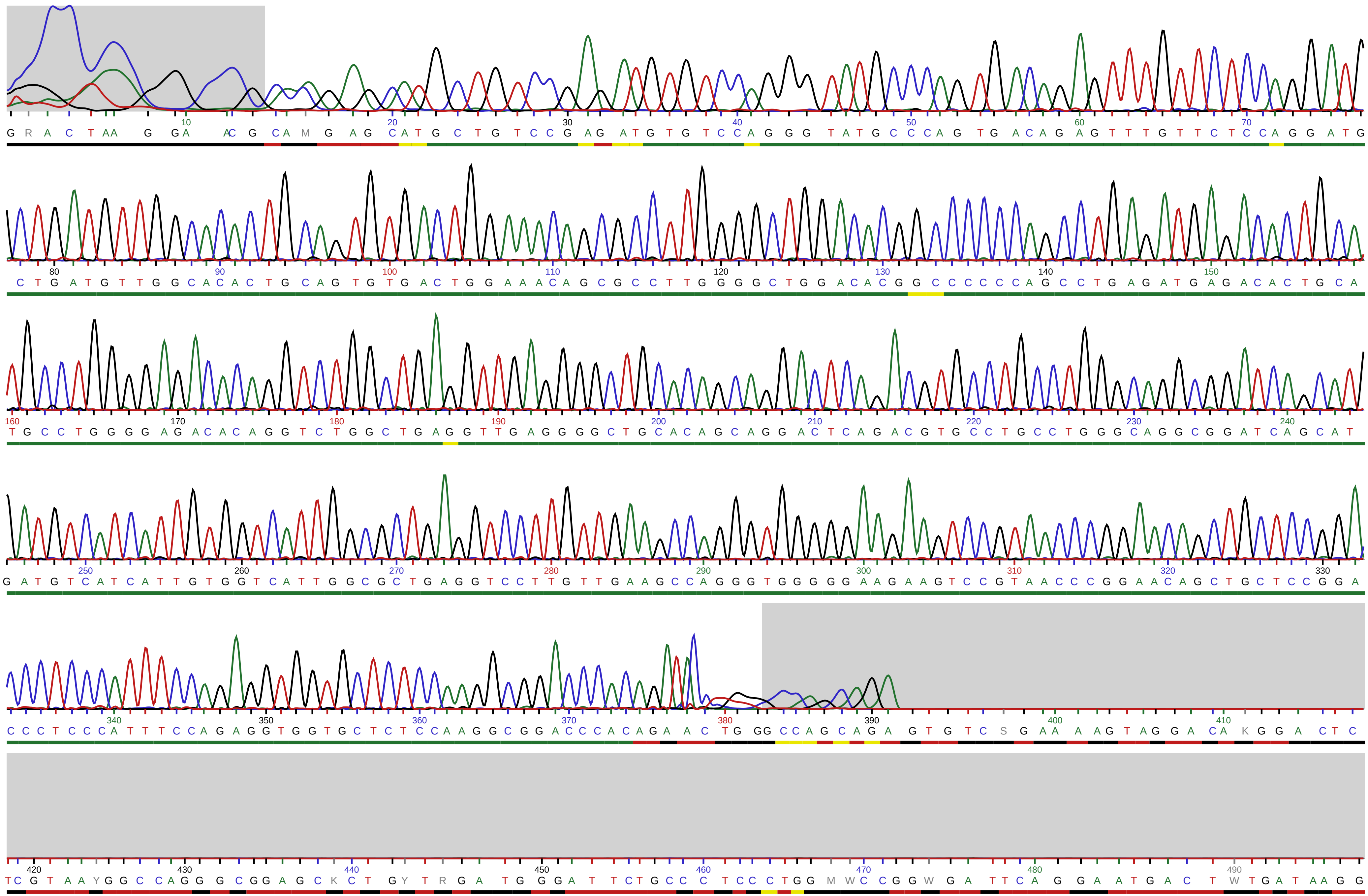

Clip. 1 BQ 20 WL 10 Sequence: KLK9 V6 Forward

Clipped length: 352
Left clip: 19
Right clip: 370
Avg. qual. in clip.: 40.75

Samples: 12964
Bases: 883
Average spacing: 15.0
Average quality >= 10: 261, 20: 97, 30: 303

Quality: 0 - 9
10 - 19
20 - 29
>= 30

03.06.2017

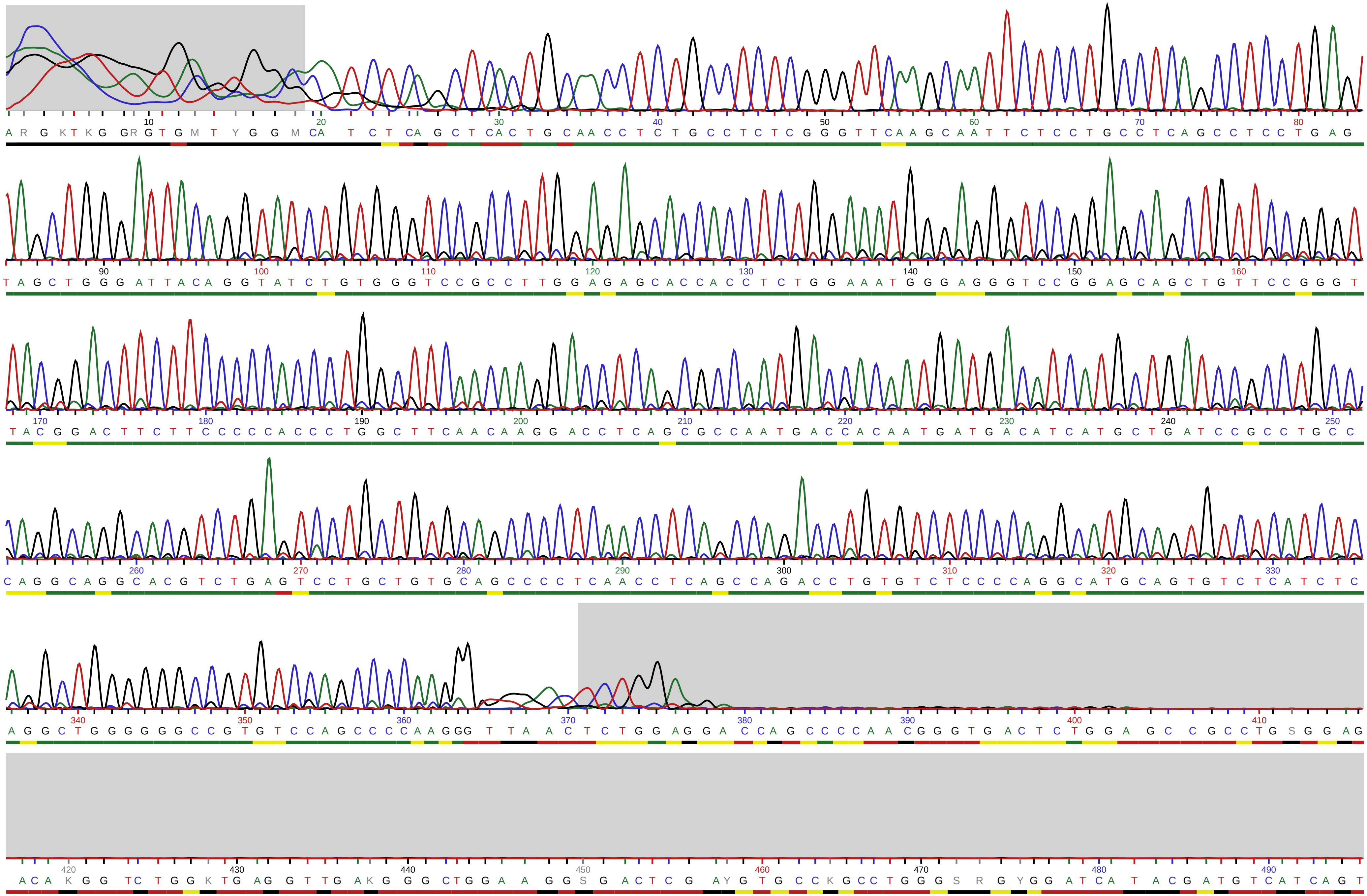

Clip. 1 BQ 20 WL 10 Sequence: KLK9 V6 Reverse

Clipped length:	348	Samples:	12965	Quality:	0 - 9	Page: 1 / 3
Left clip:	24	Bases:	388		10 - 19	22.09.2016
Right clip:	371	Average spacing:	34.0		20 - 29	
Avg. qual. in clip.:	44.62	Average quality >=	10: 21, 20: 46, 30: 288		>= 30	

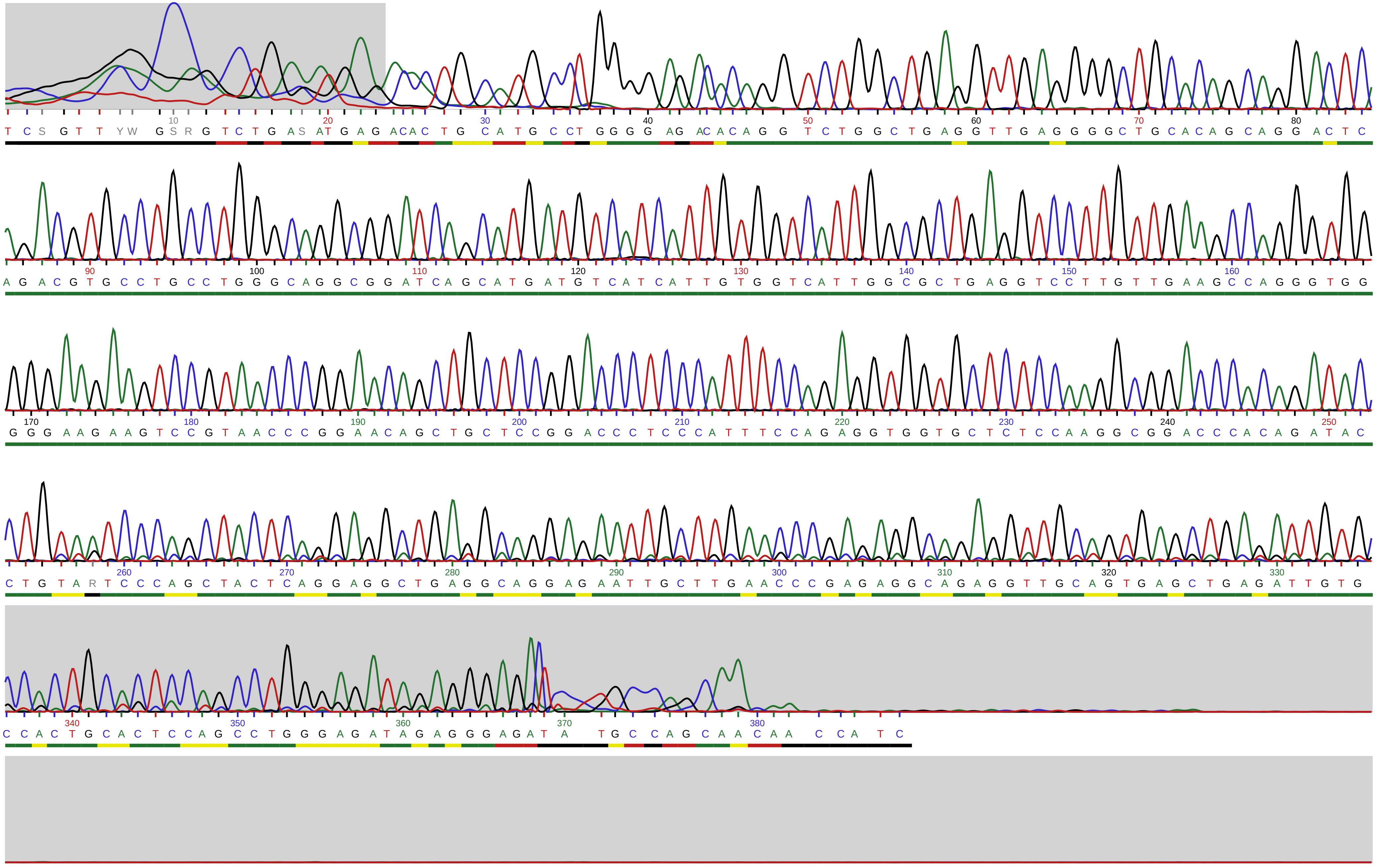

Clip. 1 BQ 20 WL 10 Sequence: KLK9 V7 Forward

Clipped length:	491	Samples:	12967	Quality: 0 - 9	Page: 1 / 3
Left clip:	16	Bases:	854	10 - 19	03.06.2017
Right clip:	506	Average spacing:	16.0	20 - 29	
Avg. qual. in clip.:	52.91	Average quality >=	10: 173, 20: 41, 30: 468	>= 30	

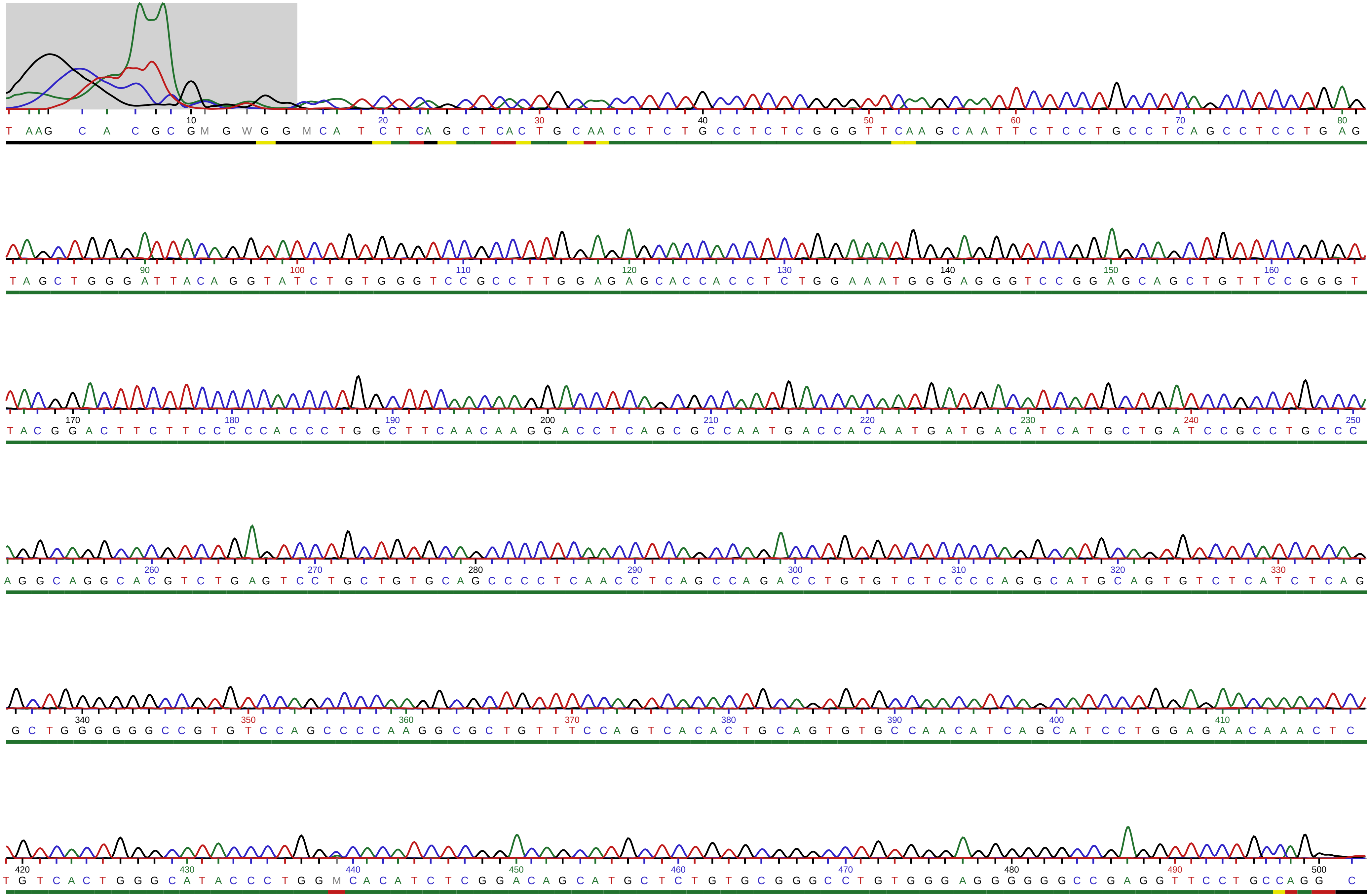

Clip. 1 | BQ 20 WL 10 | Sequence: KLK9 V7 Forward

Clipped length:	491	Samples:	12967	Quality:	0 - 9
Left clip:	16	Bases:	854		10 - 19
Right clip:	506	Average spacing:	16.0		20 - 29
Avg. qual. in clip.:	52.91	Average quality >=	10: 173, 20: 41, 30: 468		>= 30

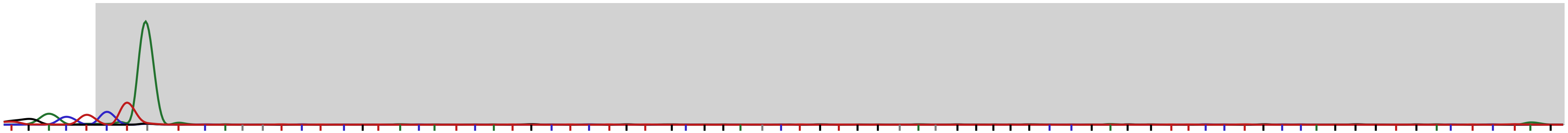

510 520 530 540 550 560 570 580
TGACTCTSTCAYRTCTCGTACATCATGCTCTGTGCGGAMCTGTGGKAKGGGGGCCGAGGTTCCTGCCAGGGTGACTCTAG

590 600 610 620 630 640 650
CTGTCTTTTCTCCTCCTAAGTCCCAGCGGGYCCCGARCTGCYCASGCYTACATASATGGATGTGGACGCTTAGRAAW

660 670 680 690 700 710 720 730
GCATCTTAGCCCATTAGAKCCWCRGGCTAMGTASTCGGATTGATSAGWGTCCTSGATCTATGTTACTCACCRGGCCT

740 750 760 770 780 790 800 810
GCACGTCTCWCKMTGGGTCCTTAAYTCTGTGTTTCTGMCTCWCTGAAATCCGTGGCTCKTKCTCWRCCCACCTGTK

820 830 840 850
GTTACWTMTMATGCCTAGGGGTACAYGCATWCAGGCCTCTTG

Clip. 1 | BQ 20 WL 10 | Sequence: KLK9 V7 Reverse

Clipped length:	476	Samples:	12964	Quality: 0 - 9	
Left clip:	24	Bases:	587	10 - 19	
Right clip:	499	Average spacing:	23.0	20 - 29	
Avg. qual. in clip.:	50.57	Average quality >=	10: 40, 20: 34, 30: 455	>= 30	

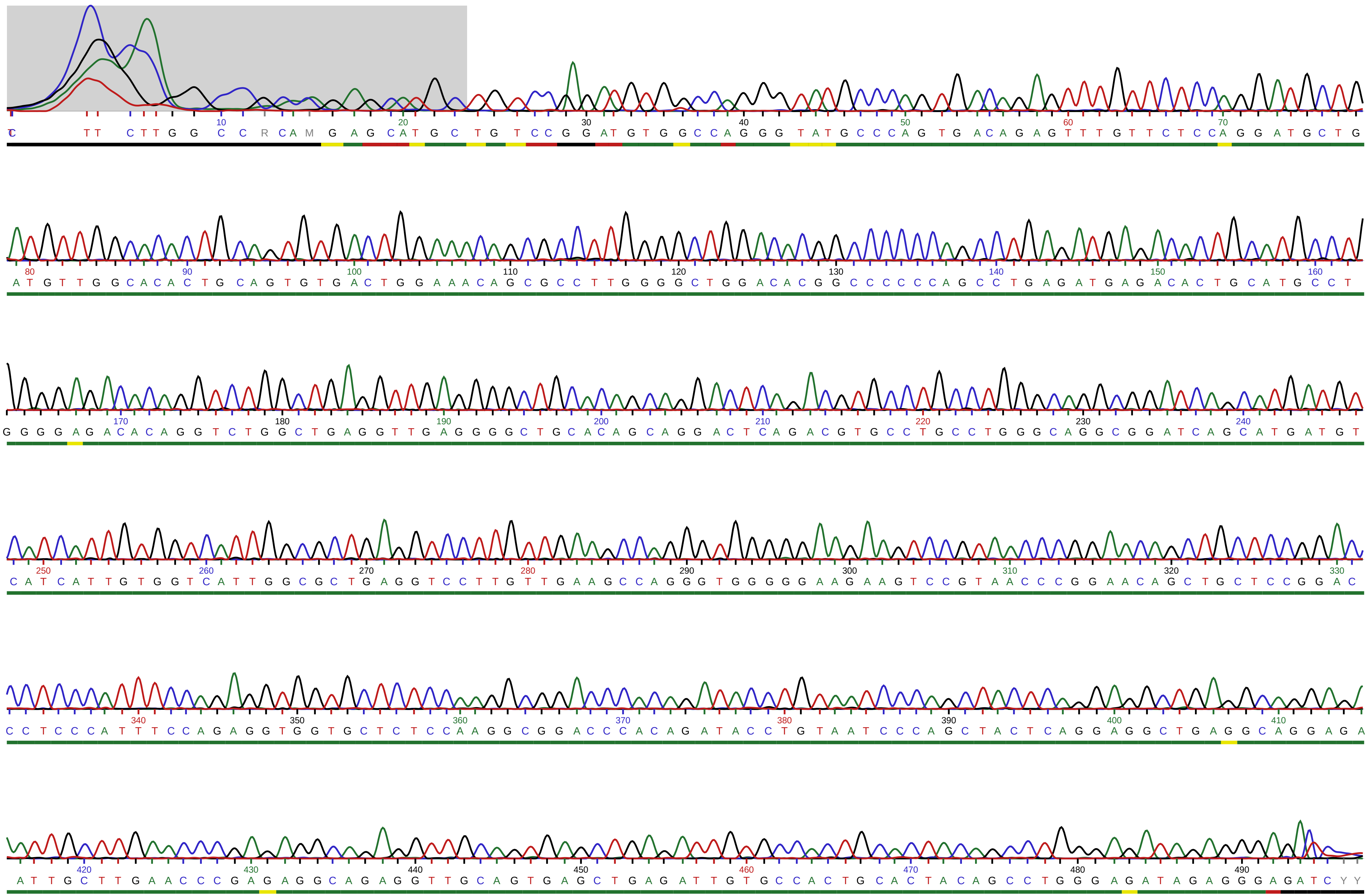

Clip. 1 BQ 20 WL 10 Sequence: KLK9 V7 Reverse

Clipped length: 476
Left clip: 24
Right clip: 499
Avg. qual. in clip.: 50.57

Samples: 12964
Bases: 587
Average spacing: 23.0
Average quality >= 10: 40, 20: 34, 30: 455

Quality: 0 - 9, 10 - 19, 20 - 29, >= 30

22.09.2016

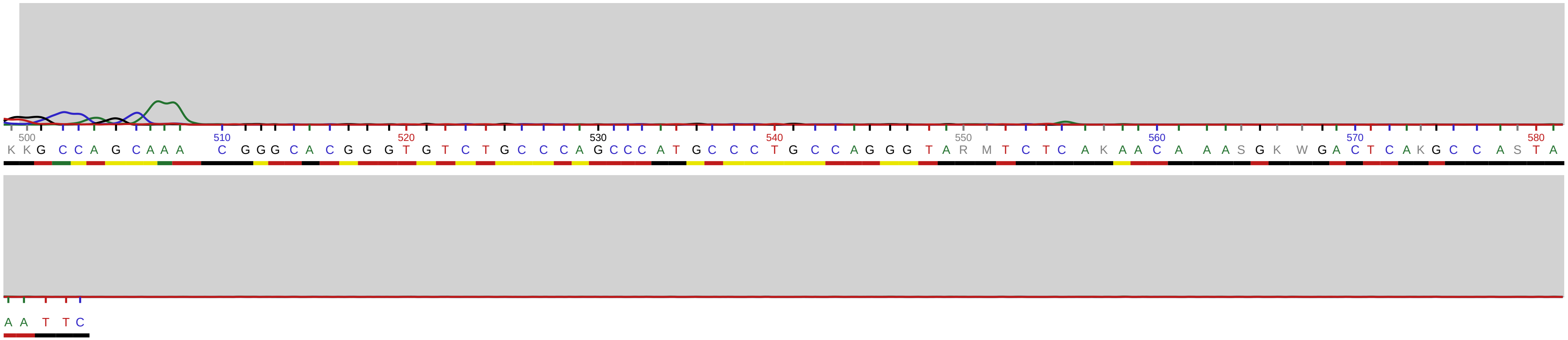

Clip. 1 BQ 20 WL 10 Sequence: KLK9 V8 Forward

Clipped length:	316	Samples:	12964	Quality: 0 - 9	
Left clip:	24	Bases:	690	10 - 19	
Right clip:	339	Average spacing:	19.0	20 - 29	
Avg. qual. in clip.:	37.62	Average quality >=	10: 185, 20: 113, 30: 260	>= 30	

03.06.2017

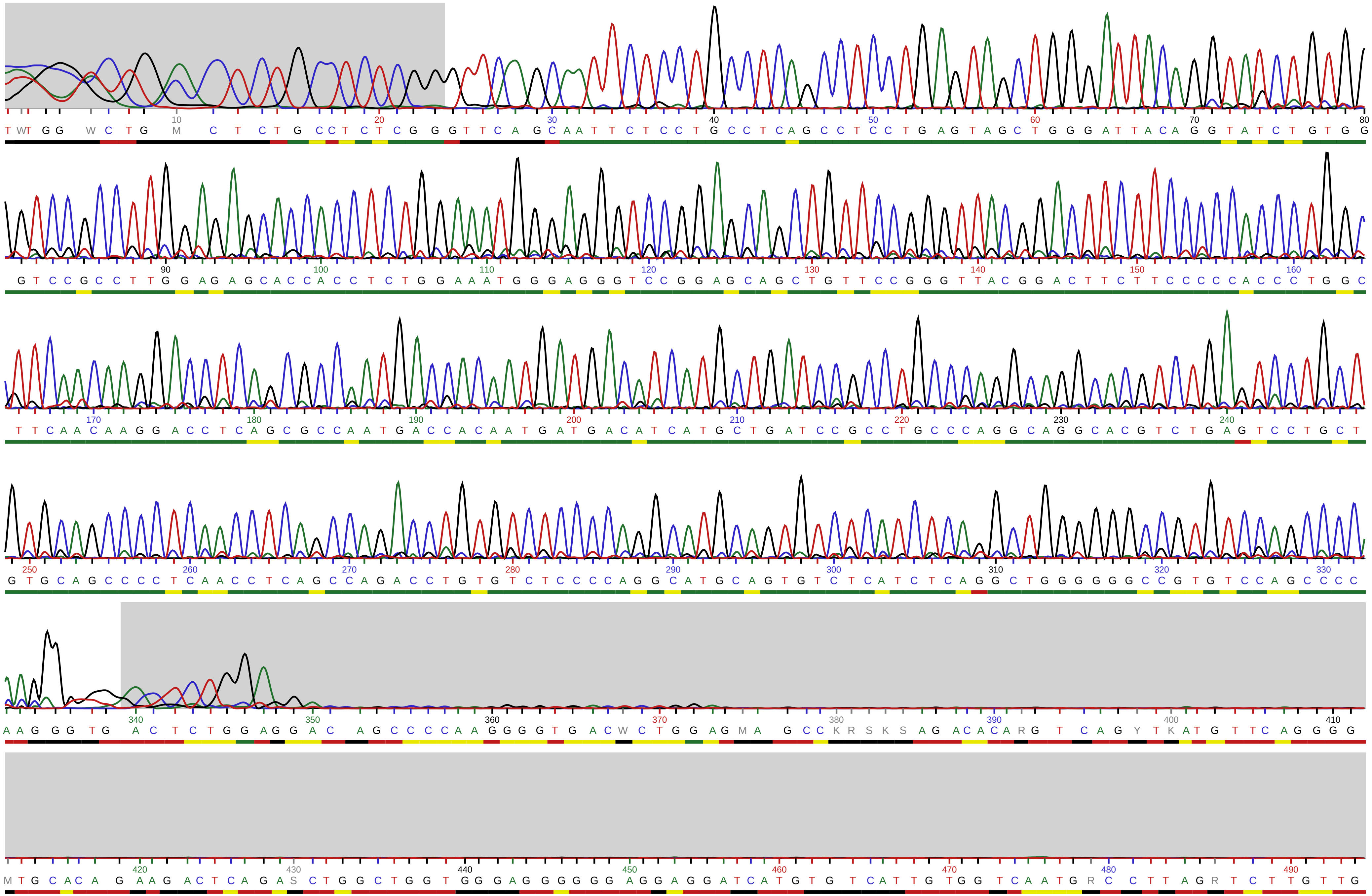

Clip. 1 BQ 20 WL 10 Sequence: KLK9 V8 Reverse

Clipped length:	327	Samples:	12965	Quality: 0 - 9		Page: 1 / 3
Left clip:	17	Bases:	436	10 - 19		22.09.2016
Right clip:	343	Average spacing:	30.0	20 - 29		
Avg. qual. in clip.:	45.02	Average quality >=	10: 48, 20: 43, 30: 275	>= 30		

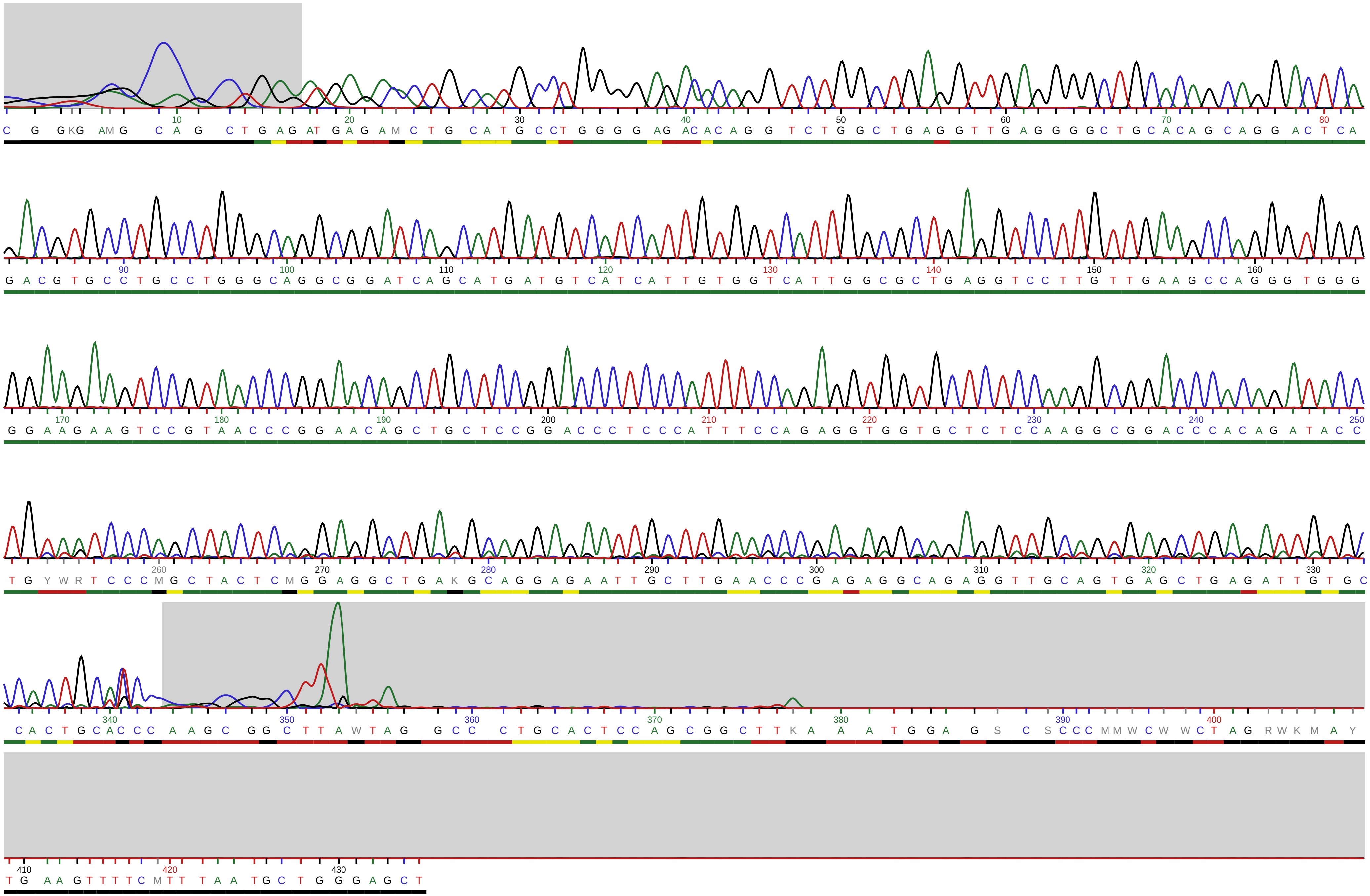

Clip. 1 BQ 20 WL 10 Sequence: KLK9 V9 Forward

Clipped length: 450
Left clip: 24
Right clip: 473
Avg. qual. in clip.: 39.05

Samples: 12966
Bases: 629
Average spacing: 21.0
Average quality >= 10: 87, 20: 78, 30: 385

Quality: 0 - 9; 10 - 19; 20 - 29; >= 30

03.06.2017

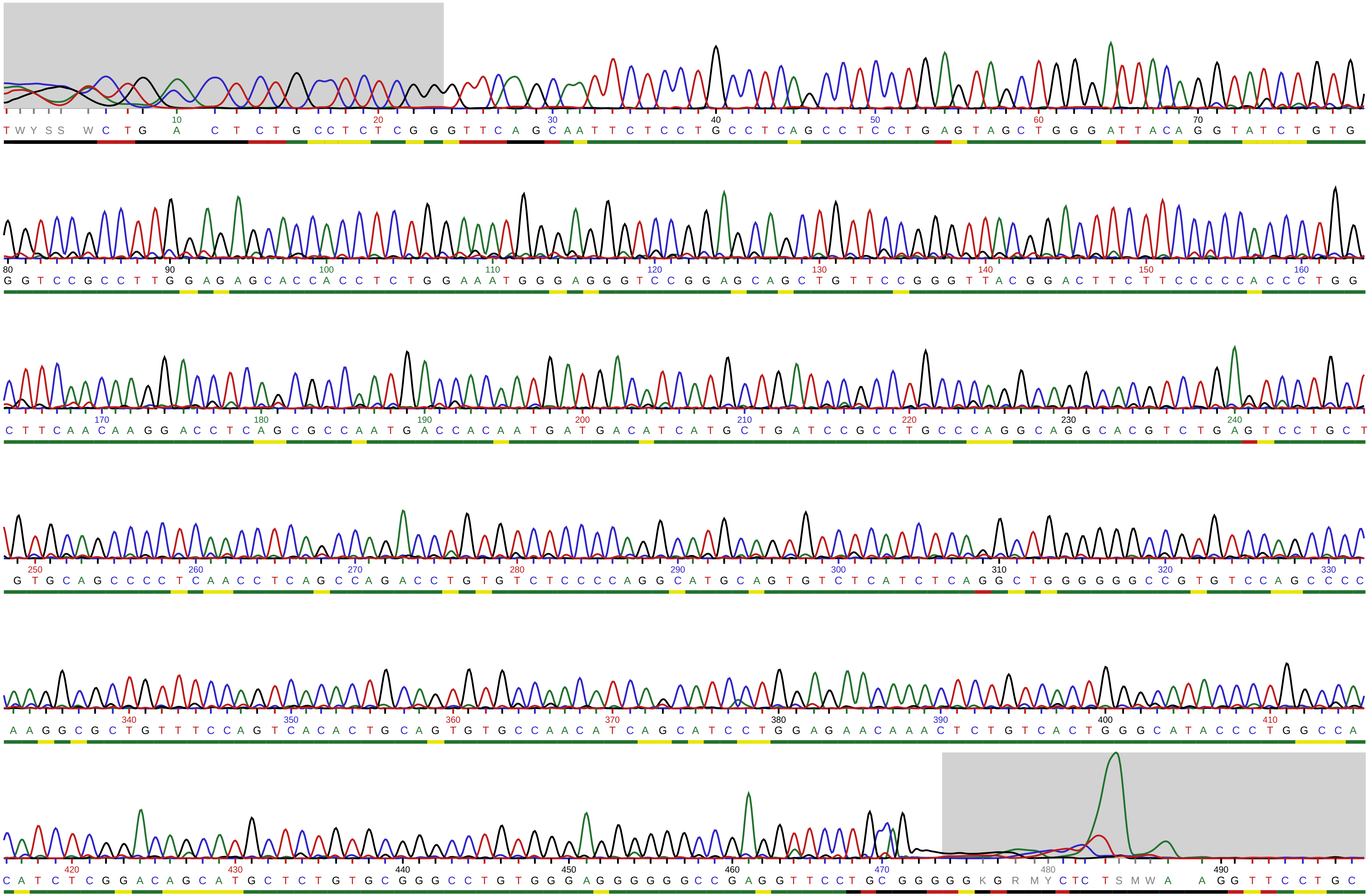

Clip. 1 BQ 20 WL 10 Sequence: KLK9 V9 Reverse

Clipped length:	460	Samples:	12965	Quality:	0 - 9	
Left clip:	14	Bases:	561		10 - 19	
Right clip:	473	Average spacing:	24.0		20 - 29	
Avg. qual. in clip.:	46.91	Average quality >=	10: 20, 20: 38, 30: 403		>= 30	

22.09.2016

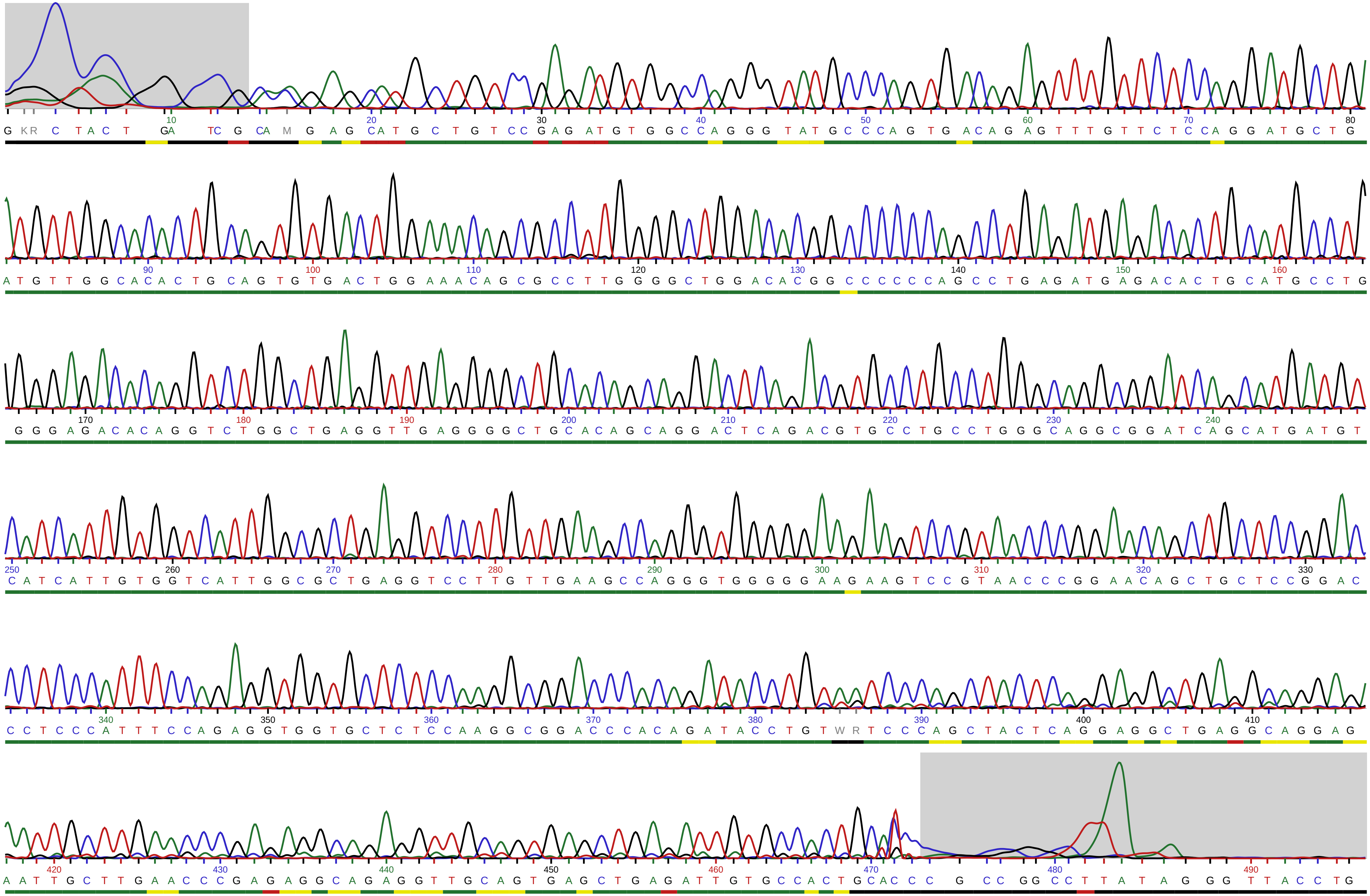

Clip. 1 BQ 20 WL 10 Sequence: KLK9 V10 Forward

Clipped length:	351	Samples:	12966	Quality:	0 - 9	Page: 1 / 3
Left clip:	19	Bases:	752		10 - 19	03.06.2017
Right clip:	369	Average spacing:	18.0		20 - 29	
Avg. qual. in clip.:	39.04	Average quality >=	10: 214, 20: 90, 30: 292		>= 30	

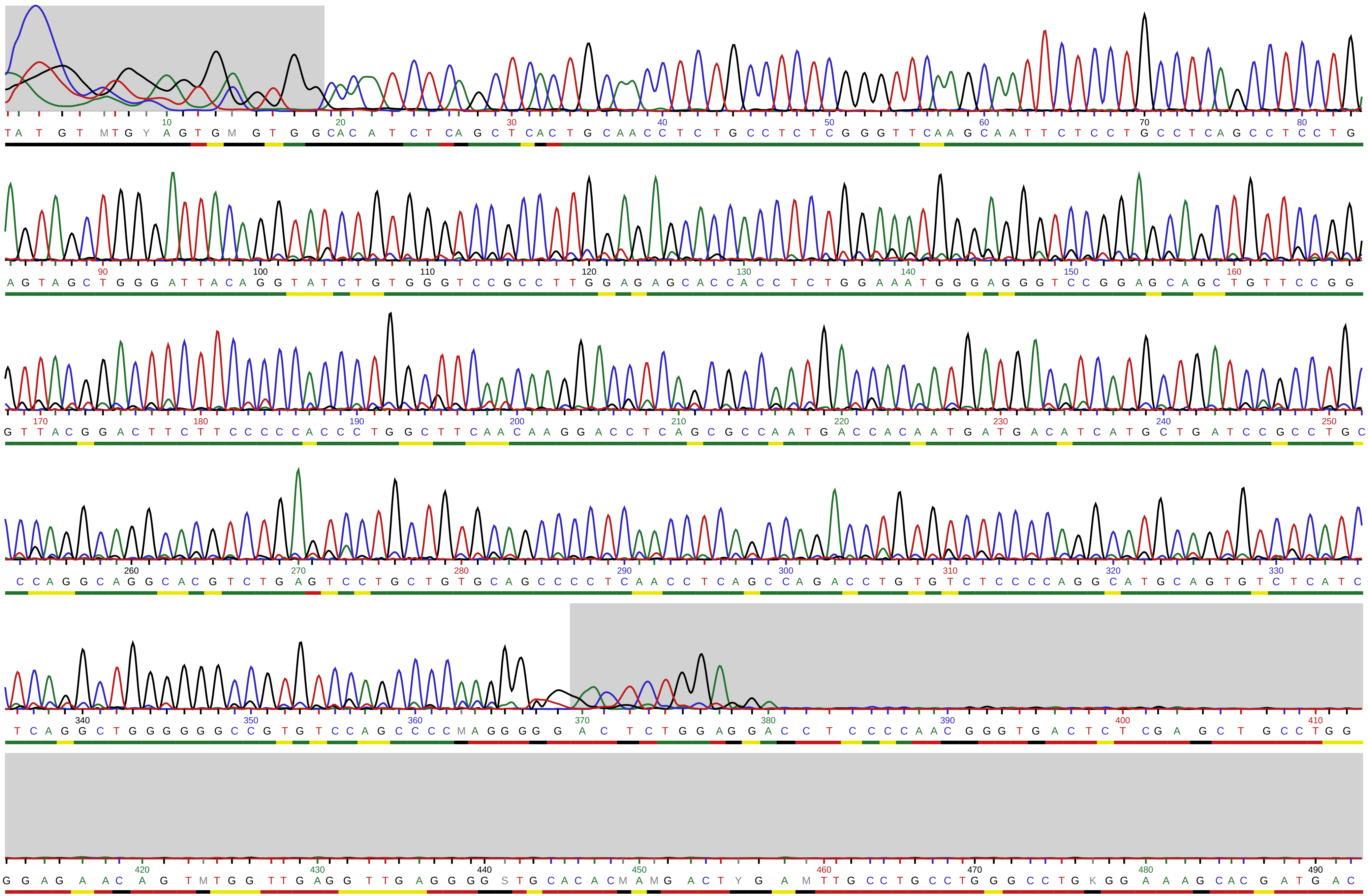

Clip. 1 BQ 20 WL 10 Sequence: KLK9 V10 Reverse

Clipped length: 357
Left clip: 16
Right clip: 372
Avg. qual. in clip.: 43.38

Samples: 12966
Bases: 391
Average spacing: 34.0
Average quality >= 10: 19, 20: 50, 30: 291

Quality: 0 - 9
10 - 19
20 - 29
>= 30

22.09.2016

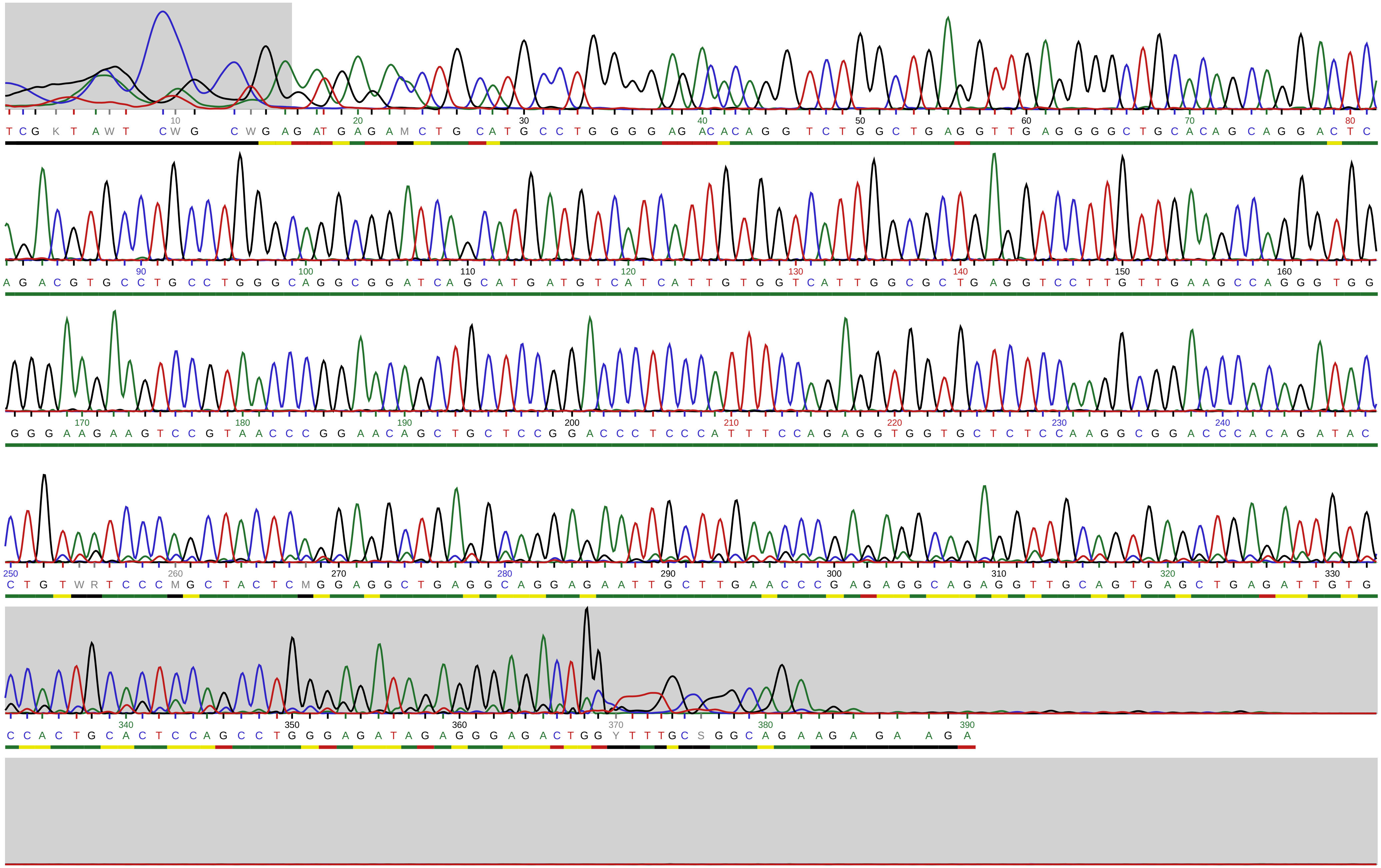

Clip. 1 BQ 20 WL 10 Sequence: KLK9 V11 Forward

Clipped length: 486
Left clip: 20
Right clip: 505
Avg. qual. in clip.: 39.66

Samples: 12964
Bases: 602
Average spacing: 22.0
Average quality >= 10: 35, 20: 74, 30: 406

Quality: 0 - 9 / 10 - 19 / 20 - 29 / >= 30

03.06.2017

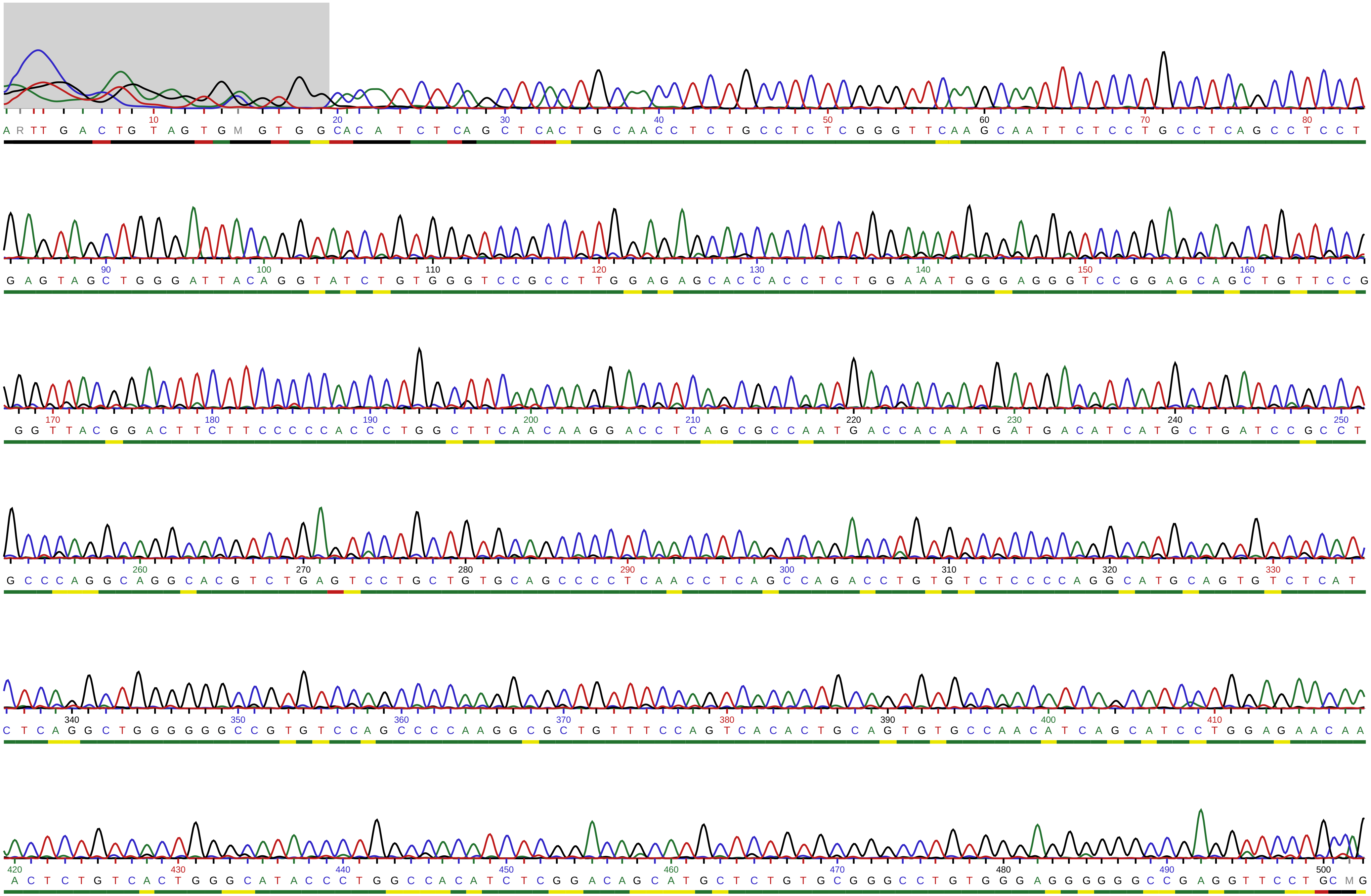

Clip. 1 BQ 20 WL 10 Sequence: KLK9 V11 Reverse

Clipped length:	491	Samples:	6657	Quality: 0 - 9	
Left clip:	14	Bases:	564	10 - 19	
Right clip:	504	Average spacing:	12.0	20 - 29	
Avg. qual. in clip.:	49.74	Average quality >=	10: 12, 20: 16, 30: 461	>= 30	

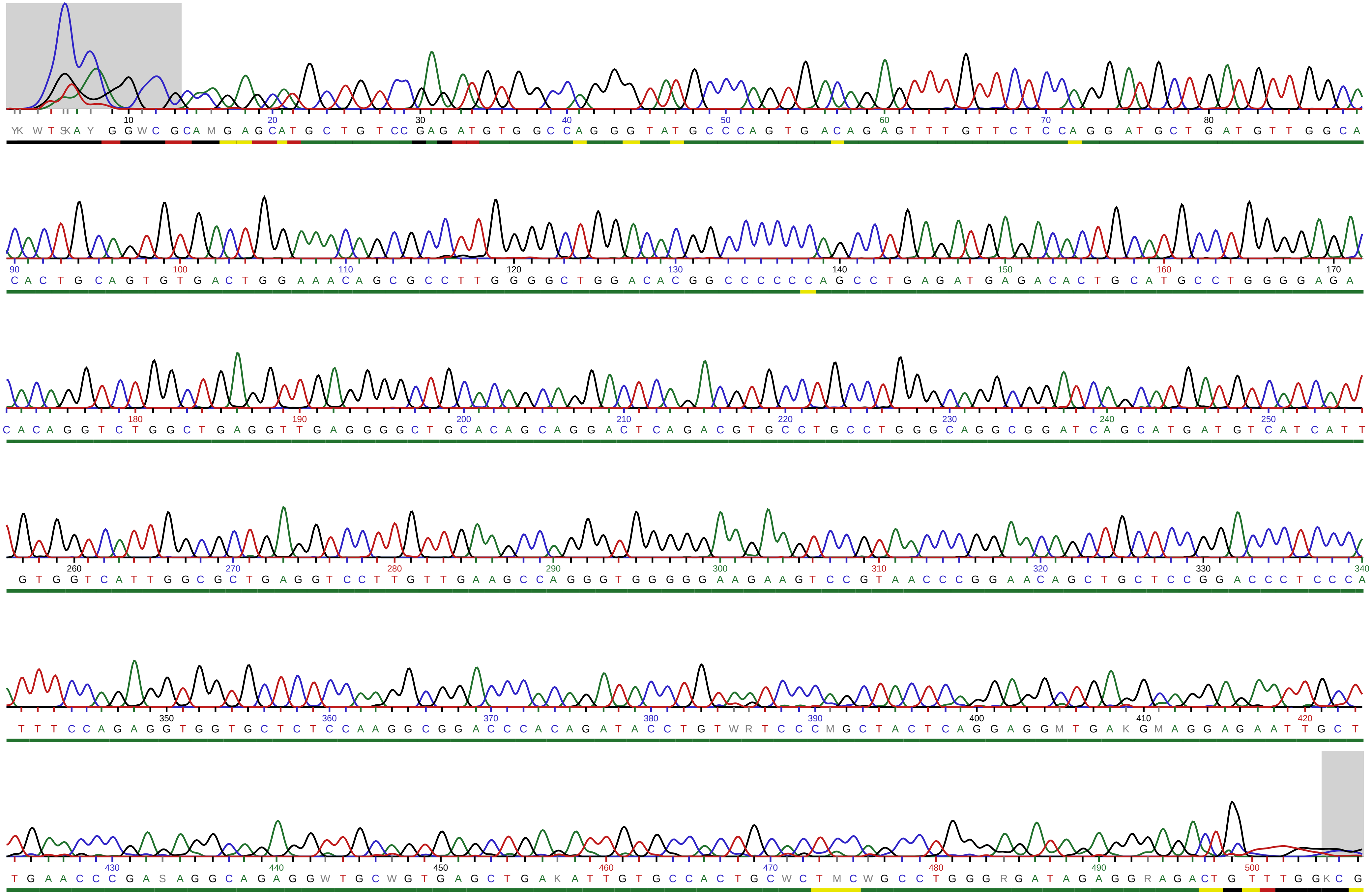

Supplement: Supplementary file 1 — Supplementary Information [file 41598_2017_16269_MOESM1_ESM.pdf]
